# CRISPR/Cas9-mediated Bag-1 knockout increased mesenchymal characteristics of MCF-7 cells via Akt hyperactivation-mediated actin cytoskeleton remodeling

**DOI:** 10.1371/journal.pone.0261062

**Published:** 2022-01-07

**Authors:** Pelin Ozfiliz Kilbas, Nisan Denizce Can, Tugba Kizilboga, Fikret Ezberci, Hamdi Levent Doganay, Elif Damla Arisan, Gizem Dinler Doganay

**Affiliations:** 1 Department of Molecular Biology Genetics and Biotechnology, Istanbul Technical University, Istanbul, Turkey; 2 Department of Molecular Biology and Genetics, Istanbul Kultur University, Istanbul, Turkey; 3 Department of General Surgery, Umraniye Teaching and Research Hospital, Istanbul, Turkey; 4 Genomic Laboratory (GLAB), Umraniye Teaching And Research Hospital, University of Health Sciences, Istanbul, Turkey; 5 Institute of Biotechnology, Gebze Technical University, Kocaeli, Turkey; Duke University School of Medicine, UNITED STATES

## Abstract

Bag-1 protein is a crucial target in cancer to increase the survival and proliferation of cells. The Bag-1 expression is significantly upregulated in primary and metastatic cancer patients compared to normal breast tissue. Overexpression of Bag-1 decreases the efficiency of conventional chemotherapeutic drugs, whereas Bag-1 silencing enhances the apoptotic efficiency of therapeutics, mostly in hormone-positive breast cancer subtypes. In this study, we generated stable Bag-1 knockout (KO) MCF-7 breast cancer cells to monitor stress-mediated cellular alterations in comparison to wild type (wt) and Bag-1 overexpressing (Bag-1 OE) MCF-7 cells. Validation and characterization studies of Bag-1 KO cells showed different cellular morphology with hyperactive Akt signaling, which caused stress-mediated actin reorganization, focal adhesion decrease and led to mesenchymal characteristics in MCF-7 cells. A potent Akt inhibitor, MK-2206, suppressed mesenchymal transition in Bag-1 KO cells. Similar results were obtained following the recovery of Bag-1 isoforms (Bag-1S, M, or L) in Bag-1 KO cells. The findings of this study emphasized that Bag-1 is a mediator of actin-mediated cytoskeleton organization through regulating Akt activation.

## Introduction

Breast cancer is the most frequently diagnosed cancer and the second leading cause of cancer-related death of women worldwide [1]. Although there are improvements in adjuvant/neoadjuvant chemotherapy models, current treatment options have limitations in the treatment of metastatic breast cancer because of the heterogeneous nature of this disease. Genomic and proteomic differences of individuals cause resistance against current therapeutics and prevent successful therapies [2]. Therefore, current knowledge of gene-editing techniques promises to overcome the genomic problems in the diagnosis and treatment of breast cancer [3].

Bag-1 (Bcl-2 associated athanogene-1) protein regulates multifarious signaling pathways involving cell proliferation, survival, transcription, apoptosis, autophagy, and metastasis through its various direct or indirect molecular partners. Bag-1 is alternatively translated from one single mRNA transcript and consists of 3 major isoforms Bag-1S (33 kDa), Bag-1M (46 kDa), and Bag-1L (52 kDa) [4, 5]. High expression profiles of each Bag-1 isoform have been observed mostly in estrogen receptor (ER) positive breast tumors and Bag-1L isoform prevents apoptosis via enhanced Bcl-2 expression in the presence of estrogen and trigger resistance phenotype in MCF-7 breast cancer cells [6]. Thus, the positive association between high Bag-1 expression and cell survival mechanism in breast cancer cells is regarded as a potential predictive marker of the clinical outcome of breast cancer [7]. Bag-1 overexpression (OE) promotes cell survival through activating c-Myc and renders efficiency of apoptotic inducers. On the contrary, our previous studies showed that transient silencing of Bag-1 enhances paclitaxel or cisplatin-mediated apoptotic cell death in MCF-7 breast cancer cells [8, 9]. In addition, overexpression of Bag-1 activates B-Raf, C-Raf and Akt through forming a protein complex with these proteins. Bag-1 led to the phosphorylation and activation of Akt which further phosphorylates Bad at Ser112 and Ser136 to inhibit apoptosis [10]. Besides, we also demonstrated that Bag-1 is a potential early autophagy marker by indirectly interacting with Beclin-1 and regulating macroautophagy [11]. Bag-1 is a co-chaperone of Hsp70 and regulates apoptosis by forming a complex structure with Akt and B-Raf [12]. However, Akt, Bag-1, and B-Raf complex cannot be formed in the neuronal cells of Bag-1-/- knockout mice due to the reduced levels of Bad phosphorylation at Ser 136 residue. The knockout of Bag-1 results in a significant increase in apoptotic neuronal cell populations, whereas high levels of Bag-1 causes chaperone-mediated long- term survival of breast cancer cells [13].

The serine/threonine-protein kinase Akt as one of the critical targets of major receptor tyrosine kinases (RTKs), modulates cell survival, proliferation, migration reactive oxygen species generation, and autophagy/apoptosis signaling pathways [14]. Activation of Akt is associated with the phenotypic transformation of epithelial cells to mesenchymal cells (EMT), which occurs during embryogenesis, and also invasion, migration, and metastasis of cancer cells [15]. Akt-mediated transactivation of β-catenin leads to its dissociation from cell-cell contact and triggers EMT via degradation of extracellular matrix proteins. In addition, loss of E-cadherin, spindle-like cell morphologies appear following cytoskeletal reorganization to improve the migration and invasion of cells. Following EMT induction mesenchymal cell-cell adhesion protein N-cadherin, vimentin, fibronectin, were also increased [16]. The excess Akt phosphorylation due to regulation of upstream receptor tyrosine kinases leads to alterations on cytoskeleton organization [17]. Hyperphosphorylation of Akt causes co-localization with actin and vimentin to increase metastatic motility of cancer cells. β-actin is characterized as a potential nuclear interaction partner of Akt and can be also phosphorylated by Akt [18, 19]. Akt is located in a protein complex consisting of B-Raf, C-Raf, and Bag-1 in breast cancer cells, which leads to inhibition of apoptosis by the phosphorylation and relocalization of Bad to nucleus [10].

Despite the evidence about survival-related functional roles of Bag-1 in breast cancer, there has been limited data about its other cellular functions. In this study, we aimed to generate a Bag-1 knockout (KO) MCF-7 breast cancer cell model using Clustered Regularly Interspaced Short Palindromic Repeats (CRISPR)/ CRISPR-associated 9 (Cas9) technique. We compared the effect of different Bag-1 expression levels (basal, knockout, and overexpressed) on the EMT signaling and actin remodeling. Bag-1 KO MCF-7 cells exhibit a slow proliferation rat compared to wt, Control KO, and Bag-1 OE cells. Moreover, we detected reduced PTEN expression, stress-mediated Akt hyperactivation and increased EMT with severe remodeling of actin cytoskeleton in Bag-1 KO cells. On the contrary, the ectopic expression of each Bag-1 isoform enhances the epithelial properties of MCF-7 cells.

## Materials and methods

### Cell culture

MCF-7 (HTB-22) and MDA-MB-231 (HTB-26) Human breast adenocarcinoma cells (ATCC, USA) were grown in high glucose DMEM (Dulbecco’s Modified Eagle Medium, Pan Biotech, Germany) supplemented with 10% FBS (Fetal Bovine Serum, Pan Biotech, Germany) and 1% 100 units/ml penicillin/ 100 μg/ml streptomycin (Pan Biotech, Germany). Cells were maintained at 37°C in a humidified 5% CO_2_ incubator. Akt inhibition experiments were performed by the treatment of 500 nM MK-2206 for 24h.

### Generation of CRISPR/Cas9-based Bag-1 knockout MCF-7 cells (Bag-1 KO cells)

MCF-7 cells were selected for knockout experiments due to their intermediate level of endogenous Bag-1 expression. Bag-1 CRISPR/Cas9 Knockout (KO) plasmid (sc-417179), Bag-1 Homology Directed Repair (HDR) plasmid (sc-417179-HDR), and Control CRISPR/Cas9 Knockout plasmid (sc-418922) was purchased from Santa Cruz Biotechnology, USA.

The Bag-1 CRISPR/Cas9 KO plasmid consists of three different Bag-1 exon-specific 20 nt guide RNA sequences which are derived from GeCKO (v2) library, with high modification efficiency and low off-target effects. The Bag-1 HDR plasmid integrates puromycin resistance genes into cut sites which are generated by the Bag-1 CRISPR/Cas9 KO plasmid. Bag-1 CRISPR/Cas9 KO and Bag-1 HDR plasmids were co-transfected into MCF-7 breast cancer cells, using UltraCruz Transfection Reagent (Santa Cruz Biotechnology, USA). GFP region was added in Bag-1 CRISPR/Cas9 KO and RFP was added in Bag-1 HDR plasmid to visualize the transfection efficiency. Therefore, both plasmids transfected cells able to have GFP and RFP intensities. Meanwhile, the Control CRISPR/Cas9 Knockout plasmid (sc-418922) which encodes Cas9 nuclease and consists of non-specific 20 nt scrambled guide RNA that can not bind to genomic DNA, was transfected into MCF-7 cells for negative control. The transfection of the Control CRISPR/Cas9 Knockout plasmid was verified through the GFP signal. After transfection, cells were treated with puromycin (Santa Cruz Biotechnology, USA) for 48h, and selected by single-cell dilution in 96-well plates. According to Bag-1 downregulation results, clones 10, 11, and clone 12 were selected. Clone 12 was used in this study to evaluate the mechanism of Bag-1 deficiency.

### Single-cell dilution

Following the transfection of CRISPR/Cas9 plasmids, cells were seeded in 96-well plates through modifying the “A single cell dilution protocol for obtaining CRISPR targeted cell clones”protocol [20]. Briefly, 1x10^4^ cells were diluted in 220 μl media, and 110 μl of the suspension was added into 890 μl fresh media. 5 cell/ml cell culture media was seeded at 100 μl into 96 well plates. Following the attachment of cells, light microscopy was used for colony selection. Expanded monoclonal cells were used for further experiments.

### Generation of stable Bag-1 overexpressed MCF-7 cells (Bag-1 OE cells) and silenced cells (Bag-1 shRNA)

To assess the effect of different Bag-1 expression levels in the cells, Bag-1 overexpressed cells (Bag-1 OE) were used in further experiments. MCF-7 cells were transfected with TAP (tandem affinity purification) tagged Bag-1L with C-terminal DNA insert plasmid as described in our previous study [8]. Bag-1L transfected stable cells were selected using G418, Geneticin (Thermo Fisher, USA), and the overexpression of Bag-1 was confirmed by immunoblotting assay (S2A Fig). In addition, to investigate the knockdown effect of Bag-1, MDA-MB-231 cells were transfected with Bag-1 shRNA plasmid (sc-29211-SH) or control shRNA as mentioned in our previous paper [9].

### Generation of transient Bag-1 isoform-specific plasmid transfections

For the recovery of Bag-1 expression in an isoform-specific manner, we transfected MCF-7 wt cells and Bag-1 KO cells with TAP-tagged Bag-1S, M, and L with N-terminal DNA insert plasmids that were cloned in pEZ-M02-plasmid (CS-L0198-M02; Capital Biosciences, Rockville, MD, USA).

For further studies, the following abbreviations were used to demonstrate selected stable MCF-7 cell lines. wt: wild-type, Control KO: Control Knockout plasmid transfected, Bag-1 KO: Bag-1 Knockout and Bag-1 HDR plasmid co-transfected, Bag-1 OE: Bag-1L C-Tap-tagged plasmid transfected stable Bag-1 overexpressed cells, Bag-1 KO+S: Bag-1S N-Tap-tagged plasmid transfected stable Bag-1 KO cells. Bag-1 KO+M: Bag-1M N-Tap-tagged plasmid transfected stable Bag-1 KO cells. Bag-1 KO+L: Bag-1L N-Tap-tagged plasmid transfected stable Bag-1 KO cells.

### Genomic PCR analyses and sanger sequencing of different exons of MCF-7 Bag-1 KO cells

Genomic DNA of MCF-7 wt, Control KO, and Bag-1 KO cells (1X10^6^) was extracted using Quick DNA/RNA Kit (Zymo Research). Primers that have the binding sites of sgRNAs were designed for exon 2, exon 3, and exon 6 (S1A Table). To detect the off-target effects, 3 different primers were also designed according to the most possible off-target sites. Primers were designed using websites CRISPOR (http://crispor.tefor.net/crispor.py) and Primer3Plus (http://www.bioinformatics.nl/cgi-bin/primer3plus/primer3plus.cgi), and were synthesized by Thermo Scientific, USA. All primers were listed in S1B Table. Specific regions of Bag-1 from MCF-7 cells were amplified by using Taq DNA Polymerase (Thermo Scientific, USA). PCR amplicons were run on %2 agarose gel. PCR products were Sanger sequenced with Applied Biosystems 3500XL Genetic Analyzer for Human Identification (Thermo Scientific, USA).

### GFP/RFP fluorescence detection

MCF-7 wt, Control KO, and Bag-1 KO cells were seeded as 5x10^4^ cells/well in 12-well plates. Following their attachments, cells were stained with 5 mg/ml DAPI for 10 min. GFP intensity of Control KO cells, GFP and RFP intensity of Bag-1 KO cells, and DAPI intensity of wt, Control KO and Bag-1 KO cells were visualized by fluorescent microscopy option of Olympus 1X71 Inverted fluorescent microscope according to excitation and emission wavelengths. (GFP Excitation: 488 nm, and Emission520 nm, RFP Excitation: 635 nm, and Emission: 675 nm, DAPI Excitation: 350 nm, and Emission: 570 nm).

### MTT assay

MTT Cell viability assay was performed to determine the viability of MCF-7 wt, Control KO, Bag-1 KO, and Bag-1 OE cells, and to investigate the cytotoxic efficiencies of Akt inhibitor (MK-2206), and the survival role of Bag-1 isoform-specific plasmid transfected cells. Cells were seeded as 1x10^4^ cells/well in 96-well plates and maintained for their attachments for 48h. Then 5 mg/ml MTT reagent (3-(4,5-Dimethylthiazol-2-yl)-2,5-Diphenyltetrazolium Bromide) was added into the wells and cells were incubated at 37°C for 4 h. After removal of MTT reagent including media from the wells, 100 μl DMSO (dimethylsulfoxide) was added to each well and incubated for 5 min at dark conditions. Absorbance was measured at 570 nM using iMark Microplate Absorbance Reader (Bio-Rad).

### Trypan blue dye exclusion assay

To determine the cell proliferation rate of MCF-7 wt, Control KO, Bag-1 KO, and Bag-1 OE cells, and to investigate the effect of MK-2206, cells were seeded at 5x10^4^ cells/well density in 6-well plates. According to the time points (24 h, 48 h, 72 h), cells trypsinized and counted every 24 h after staining with 0.4% trypan blue dye by using a Neubauer dual-chamber hemocytometer under the light microscope. Trypan blue-stained cells were considered non-viable and were excluded from the calculation.

### Colony formation assay

To investigate the role of Bag-1 expression on colony-forming potentials of wt, Control KO, Bag-1 KO, and Bag-1 OE cells, 500 cells/well were seeded in 6 well plates and maintained for 14 days to form colonies. After cells were trypsinized and fixed with methanol: acetic acid (3:1 ratio) for 5 min, the fixing agent was removed and cells were stained with 0.5% crystal violet in methanol for 15 min. Following the last washing step, morphological images were taken under light microscopy.

### Receptor tyrosine kinase signaling antibody array

To investigate the effect of different Bag-1 expression levels on kinase phosphorylation profiles and downstream signaling nodes of MCF-7 cells, we used PathScan RTK Signaling Antibody Array Kit (#7982, Cell Signaling Technology) according to the manufacturer’s instructions. Briefly, this slide-based antibody array kit allows simultaneous detection of 39 proteins when phosphorylated at tyrosine or other residues. Total protein lysates (1 mg/ml) were incubated on an array slide. LumiGLO^®^ and Peroxide Reagent were used to detect the bound phosphorylated proteins by chemiluminescence. The slides were visualized by ChemiDoc (Bio-Rad) imaging system. The densitometry analysis was performed by ImageJ (https://imagej.nih.gov/ij/). Each spot was selected manually and the average pixel density of two replicate spots was calculated. Background pixel density was subtracted from each spot. The signal intensities were normalized to the pixel density of positive control. Significance was determined using a cut-off point of density signal higher than 0.4.

### Measurement of cellular ROS generation

To measure the cellular Reactive Oxygen Species (ROS), oxidation of a ROS indicator 2’,7’-dichlorodihydrofluorescein (H2-DCFHDA) was followed. The non-fluorescent H_2_-DCFHDA is oxidized and converted into 2’,7’-dichlorofluorescein (DCF) by intracellular ROS generation, and the DCF intensity is evaluated by FACS flow. Cells were seeded at 3x10^5^ cells/well density in 6-well plates and were treated with MK-2206 (500 nM) for 24 h. The positive control was treated with H_2_O_2_ (0.1 mM) for 20 min, instead of MK-2206. Cells were trypsinized and stained with 0.1 μM H_2_-DCFHDA for 30 min at 37°C. The intensity was measured using a 488 nm excitation filter and detected at 535 nm.

### Immunofluorescence experiment

To compare the organization of actin cytoskeleton in MCF-7 wt, Bag-1 KO, Bag-1 OE, and Bag-1 isoform-specific plasmid transfected wt and Bag-1 KO cells, Actin Cytoskeleton and Focal Adhesion Staining Kit (FAK100, Merck, USA) were used according to the manufacturers’ instructions. Briefly, cells were seeded at 50% confluency in 6-well plates and fixed with 4% paraformaldehyde. Following permeabilization with 0.1% Triton X-100, cells were blocked with 1% BSA and incubated with fluorescent-labeled Phalloidin (TRITC-conjugated Phalloidin) to observe the filamentous F-actins, which are the major constituent of microfilaments. After incubation, cells were stained with DAPI for 5 min and fluorescent images were captured with a fluorescent microscopy option of Olympus 1X71 inverted fluorescent microscope. F-actin was detected using TRITC-conjugated Phalloidin which was observed in red staining, and the nuclear staning was shown in blue which was detected by DAPI.

### Total protein extraction from tissues

The tissue samples were collected from 8 female breast cancer patients registered in Umraniye Training and Research Hospital (UEAH) between 2016 and 2018. Informed consent was obtained from all subjects involved in the study, which was conducted according to the guidelines of the Declaration of Helsinki, and approved by the Ethics Committee of University of Health Sciences Istanbul Umraniye Teaching and Research Hospital (UEAH), protocol code BD8082998622/4864. After surgery (mastectomy and lumpectomy) of the patients, fresh primary tumor and neighboring normal breast tissues were taken from the dissection material. Tissue samples were stored into RNA Later Tissue Stabilization Solution (Ambion) at -20°C. Frozen tissue samples were homogenized by grinding with liquid nitrogen and lysed in Tissue Protein Extraction Reagent, T-PER^™^(Thermo Scientific, USA) augmented with 1x PhosSTOP (Roche) and 1x cOmplete Protease Inhibitor Cocktail (Roche). Then, the homogenates were centrifuged at 10000 g at 4°C for 5 min, and the supernatants were collected. Total protein concentration was measured by Bradford assay.

### Total protein extraction from cells

MCF-7 wt, Control KO, Bag-1 KO, Bag-1 OE, MDA-MB-231 wt, Control KO, Bag-1 KO, Control shRNA, and Bag-1 shRNA cells were seeded as 1x10^6^/well in 60 mm petri dishes. Then, cells were washed with ice-cold 1x PBS (Phosphate-buffered saline, PAN Biotech, Germany) and lysed using M-PER^™^ Mammalian Protein Extraction Reagent (Thermo Scientific, UK) supplemented with 1x protease inhibitor cocktail (cOmplete, Mini, Sigma Aldrich, USA) according to the manufacturer’s instructions. The protein concentrations were measured by Bradford assay.

### Immunoblotting assay

30 μg total cell lysates and 20 μg total breast tissue lysates were boiled for 5 min with 5x Laemmli Sample Buffer at 95°C, fractionated on a 12% SDS-PAGE, and transferred to PVDF membranes (Thermo Scientific, USA). After blocking with 5% BSA in TBS-Tween 20 for 1h at room temperature, membranes were incubated with primary antibodies (Cell Signaling Technologies, USA and Abcam, UK) overnight at 4°C, and anti-rabbit or anti-mouse IgG-horseradish peroxidase-conjugated secondary antibodies (Cell Signaling Technologies, USA) overnight at 4°C. Following a gentle washing step with TBS-Tween 20, the membranes were treated with enhanced chemiluminescence detection reagent and analyzed using Chemidoc MP Imaging System (Bio-Rad, USA). Densitometric analyses were performed using Image Lab Software (Bio-Rad, USA). All proteins were quantified relative to the GAPDH internal loading control. The information about the primary antibodies used in this study was as the following: Bag-1 (3.10G3E2) Mouse mAb #3920, GAPDH (D16H11) XP^®^ Rabbit mAb #5174, phospho-Akt (Ser473) (D9E) XP^®^ Rabbit mAb #4060, phospho-Akt (Thr308) (D25E6) XP^®^ Rabbit mAb #13038, pan-Actin Antibody #4968, β-Actin (D6A8) Rabbit mAb #8457, PI3 Kinase p85 Antibody #4292, PTEN (138G6) Rabbit mAb #9559, phospho-PTEN (Ser380) Antibody #9551, Akt Antibody #9272, p38 MAPK (D13E1) XP^®^ Rabbit mAb #8690, phospho-p38 MAPK (Thr180/Tyr182) (D3F9) XP^®^ Rabbit mAb #4511, E-Cadherin (24E10) Rabbit mAb #3195, N-Cadherin (D4R1H) XP^®^ Rabbit mAb #13116, ZEB1 (D80D3) Rabbit mAb #3396, ZO-1 (D6L1E) Rabbit mAb #13663, TWIST1 Anti-Twist [Twist2C1a] (ab50887), FAK Antibody #3285, phospho-FAK (Tyr397) Antibody #3283, Paxillin #2542, CD133 (D2V8Q) XP^®^ Rabbit mAb #64326, α-Actinin (D6F6) XP^®^ Rabbit mAb #6487, Anti-Cofilin (ab54532), phospho-cofilin (ab12866), Vimentin (D21H3) XP^®^ Rabbit mAb #5741, Vinculin Antibody #4650.

### Real-time quantitative reverse transcriptase PCR

Total RNA of wt, Bag-1 KO, Bag-1 OE cells and S. M and L isoform transfected Bag-1 KO cells were extracted with PureZOL RNA Isolation Reagent (Bio-Rad) according to the manufacturer’s instructions. Total RNA samples were converted to cDNA by using iScript Reverse Transcription Supermix for RT (Bio-Rad). Quantitative real-time PCR was performed using SYBR Green Master Mix (Bio-Rad) and analyzed on a CFX Connect Real-Time System (Bio-Rad). Relative target mRNA expressions were normalized to the level of GAPDH mRNA as an internal control. All experiments were performed in three biological and two technical replicates.

### Transwell cell migration assay/Boyden chamber

Transwell chamber: 24-well, 8 μm pore membranes (Corning) were used to investigate the migration potential of wt, Bag-1 KO, and Bag-1 OE cells. 2.5x10^5^ cells were seeded in the upper part of the Transwell chamber with 200μl serum-free media. 750 μl complete medium was added into the lower chamber in a 24-well plate. Cells were incubated at 37°C in a humidified 5% CO2 incubator for 24h. Cells on the upper surface were washed with 1xPBS, and were fixed with 3.7% formaldehyde. Permeabilization of cells was maintained by incubation with 100% methanol for 20 min. After washing with 1xPBS cells were stained with %0.5 crystal violet for 15 min. Following the last washing step, the non-migratory cells on the upper part of the chamber were scraped with a cotton swab. The number of cells that invaded to the lower part of the filter membrane were visualized at 100x magnification under a light microscope Olympus 1X71.

### Wound healing/Scratch assay

The wound healing assay was performed to investigate the cell migration of wt, Control KO, Bag-1 KO and Bag-1 OE cells in vitro. Basically, 6x10^5^ cells/well were seeded into a 6-well plate and maintained until their at least 80% confluency. Plates were straightly scratched using a 200 μl sterile pipette tip. The wound closure was measured in time dependent manner to quantify the migration rate of cells and imaged by light and fluorescence microscopy (Olympus IX70).

### Data analysis

All the statistical analyses of experiments were performed using GraphPad Prism version 8, https://www.graphpad.com/. MTT Cell viability assay (Fig 2A) was performed as three replicates and statistically analyzed using Two-Way ANOVA Sidak’s multiple comparison tests. Trypan Blue Dye Exclusion Assay (Fig 2B) analyzed by Two-Way ANOVA Dunnett’s multiple comparison test. Path Scan analysis (Fig 3) was performed by Two-Way ANOVA, Tukey’s multiple comparison tests. The densitometric calculations of three replicates of immunoblotting images were performed using Image Lab Software (Bio-Rad) and analyzed by Two-Way ANOVA, Tukey’s multiple comparison test. Differences were considered statistically significant when *p < 0.05; **p < 0.01; ***p < 0.001; and ****p < 0.0001. Error bars represent ± standard deviation values.

## Results

### Generation and validation of Bag-1 knockout MCF-7 cell line

**Fig 1:**
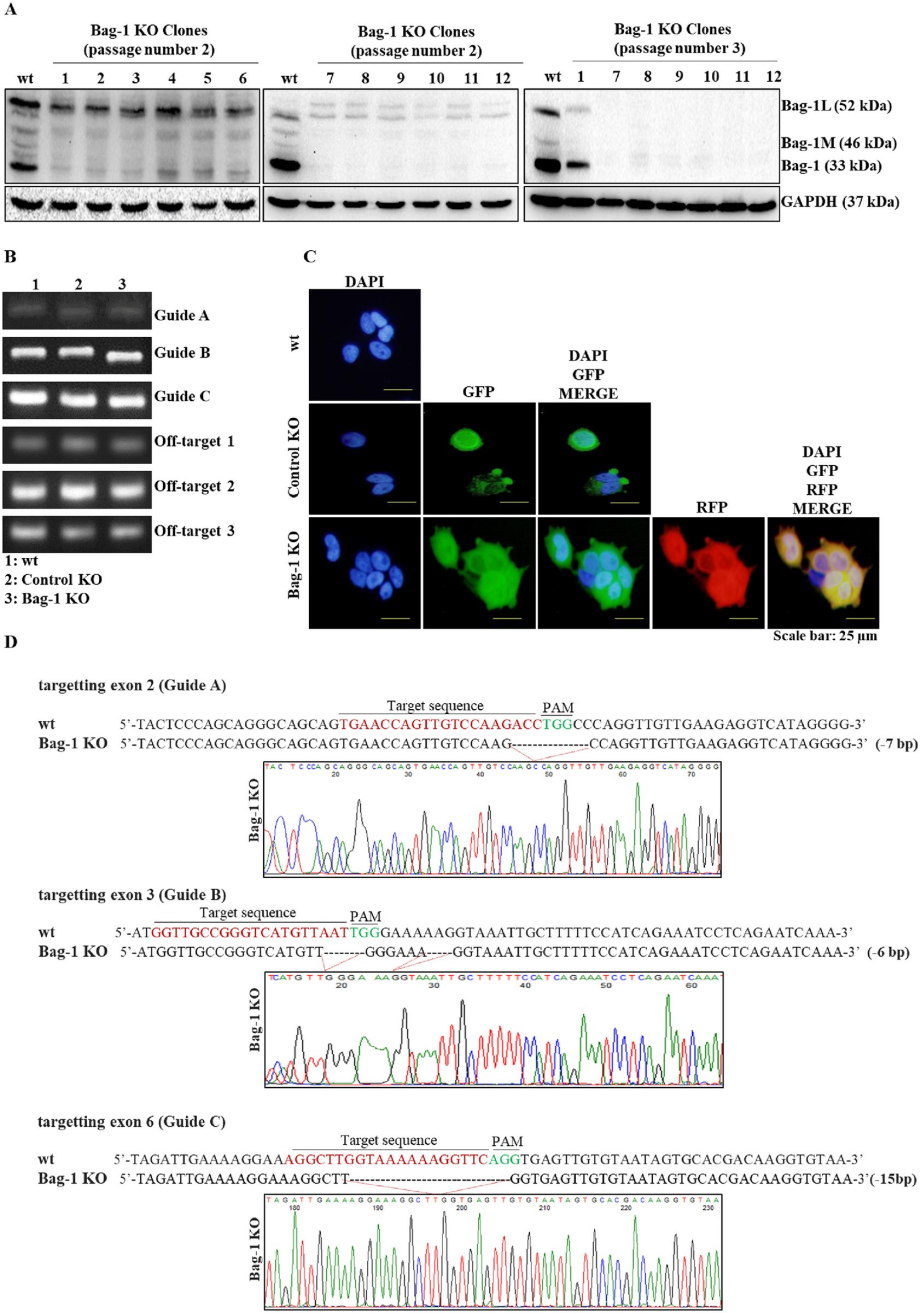
Generation and validation of Bag-1 KO MCF-7 cells. A) Identification of selected clones that exhibit loss of Bag-1 expression by immunoblot analysis of cellular Bag-1 KO cell lysates. GAPDH was used as a loading control. The relative densitometry analysis represented the mean ± SD of three independent experiments. (** p = 0.0044, *** p = 0.0005, **** p< 00001 by Two-way ANOVA, Tukey’s multiple comparison test, S1E Fig). B) Characterization of base pair differences in selected Bag-1 KO and Control KO cells by genomic PCR analysis. C) Verification of selected Control KO and Bag-1 KO clones by GFP and RFP fluorescent protein intensity. DAPI was used for the nuclear formation of cells. Scale bar is 25 μm and magnification 40x. D) Detailed deletion analysis of Bag-1 KO cells by Sanger DNA sequencing of genomic DNA PCR products targeting different sgRNAs.

Bag-1 KO MCF-7 cells were generated by co-transfection of Bag-1 KO plasmid (sc-417179) and Bag-1 HDR Plasmid (sc-417179-HDR). Three sgRNAs on Bag-1 KO plasmid target exon 2 (sgRNA A), exon 3 (sgRNA B) and exon 6 (sgRNA C) (S1A and S1B Table). Transfected cells were selected with puromycin (1–10 μg/ml), which allows the assortment of cells that were transfected successfully. Then, cells were further cloned through a single-cell dilution process, and 12 clones from generated Bag-1 KO cells were selected with respect to plasmid transfection. The efficiency of generated Bag-1 KO MCF-7 cell clones validated by immunoblotting assay, genomic PCR and DNA sequencing analysis (Fig 1). Standard PCR analysis showed that the majority of Bag-1 KO clones produced smaller PCR products in comparison to wt cells (S1A Fig). Confirmation of selected Bag-1 KO clones that exhibit loss of Bag-1 expression in different cell lysates from further passages of cells was performed by immunoblotting analysis. Bag-1 expression was not diminished in selected 1–6 Bag-1 KO clones; whereas clones 7–12 showed significantly lower Bag-1 expression profile in comparison to wt cells in passage number 2. In further passage 3, the complete loss of Bag-1 expression was observed in clone 7–12 (Fig 1A). One individual clone (Clone 12) with completely none Bag-1 expression was selected as a Bag-1 KO cell for further experiments. Then, we performed standard PCR assay targeting specific exons in the Bag-1 gene and off-target regions with wt, selected Control KO and Bag-1 KO clone to compare PCR product bp lengths. The size of the PCR products from selected Bag-1 KO clones was found to be smaller than that of wt and Control KO cells. No size differences were determined at off-target regions (Fig 1B). Bag-1 KO MCF-7 cells were co-transfected with Bag-1 KO CRISPR/Cas9 plasmid, which has a GFP region, and Bag-1 HDR plasmid, which includes the RFP region. Therefore, the transfection efficiency of both plasmids into MCF-7 cells was verified by the intensities of both proteins. The Control KO MCF-7 cells were generated by the transfection of Control CRISPR/Cas9 Knockout plasmid that have only the GFP region. Thus, in transfected Control KO cells only GFP intensities were determined. Nuclear formations of cells were visualized by DAPI staining (Fig 1C). To confirm the gene-editing efficiency for sgRNA A, B, and C, DNA sequencing results were aligned to the wt DNA sequence of exon 2, 3 and 6, respectively. The target sequences, PAM sequences, and deleted regions were highlighted to show the deletions on three targeted sites. Guide A primer showed 7 bp deletions, Guide B primer showed 6 bp deletions and Guide C-used PCR product showed 15 bp deletions in forwarding strand comparing original Bag-1 gene sequences (Fig 1D). Each of three different sgRNAs also caused deletions in reverse strands at the same sites as expected (S1B, S1C and S1D Fig).

### Knockout of Bag-1 caused cell proliferation delay and colony formation inhibition of MCF-7 cells

**Fig 2:**
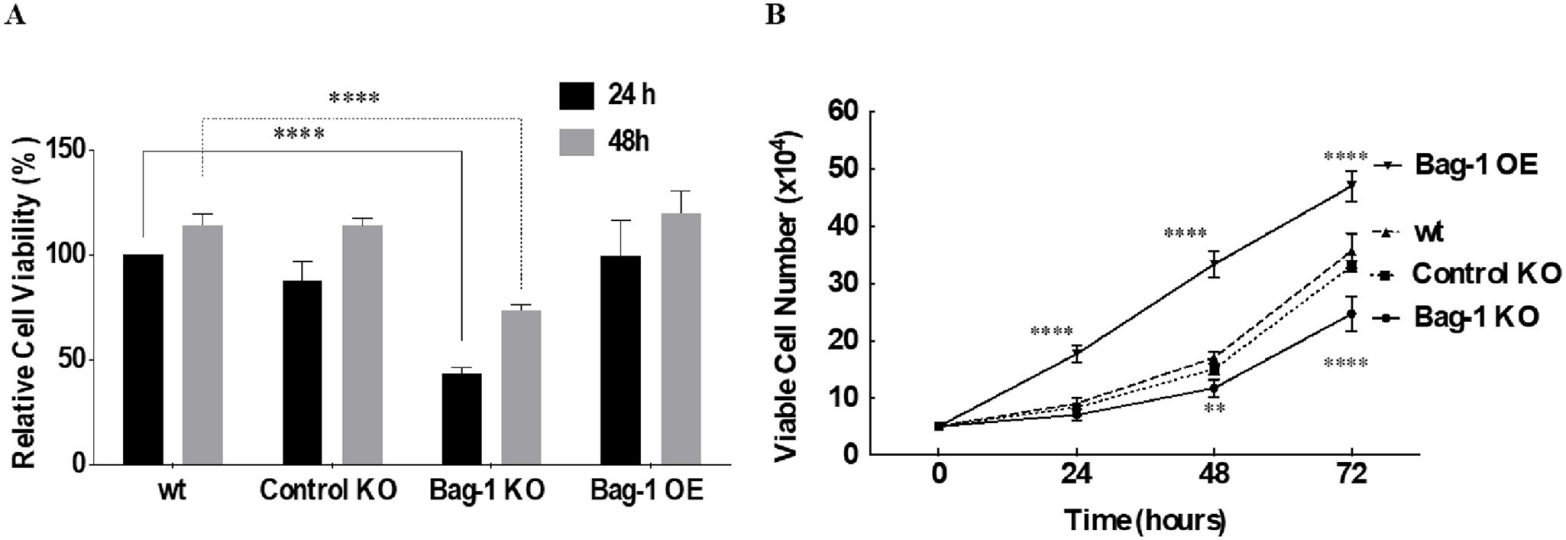
The knockout of Bag-1 suppressed the cell survival and proliferation of MCF-7 cells. A) Cell viability of wt, Control KO, Bag-1 KO, and Bag-1 OE cells was performed by MTT assay. The bar histograms represented the mean ± SD of three independent experiments with at least four replicates (**** p< 00001 by Two-way ANOVA, Sidak’s multiple comparison test). B) Cell proliferation of wt, Control KO, Bag-1 KO and Bag-1 OE was determined by Trypan Blue Dye Exclusion assay in a time-dependent manner (0–72 h). The mean ± SD of three repetitive experiments were analyzed by Two-way ANOVA, Tukey’s multiple comparison test. (**, p = 0.0020, **** p<0.0001).

To investigate the potential biological effects of Bag-1 loss on MCF-7 breast cancer cells, we utilized cell sensitivity assays such as MTT and trypan-blue dye exclusion assay. We examined the relative cell viability of each condition at 24 h and 48 h in comparison to wt cells. Bag-1 KO cells showed less cell density with the lower cell viability ratio compared to wt cells (**** p<0.0001) in 24 h. The longer seeding of cells for 48 h increased the density of cells in wt, Control KO, Bag-1 KO and Bag-1 OE cells (Fig 2A). We concluded that maintaining Bag-1 KO cells for 24 h was not sufficient for the adherence of cells to petri dishes. Then, we checked the proliferation ratie of wt MCF-7, Control KO, Bag-1 KO and Bag-1 OE cells in a time-dependent manner (0–72 h). Our results showed that the viable cell number of wt and Control KO cells increased in each time period with no statistically meaningful difference. Bag-1 KO showed a cytostatic effect within 24 h, but longer plating of Bag-1 KO cells showed an increased proliferation rate (** p = 0.0020, **** p<0.0001), which was slower than wt or Control KO cells. On the contrary, the proliferation rate of Bag-1 OE cells was the highest compared to that of wt MCF-7 cells (**** p<0.0001) (Fig 2B). The overexpression profile of Bag-1 OE cells confirmed by immunoblotting S2A Fig. Loss of Bag-1 expression reduced colony formation of cells and their morphologies were changed with getting smaller and rounded to the expected size and elongated form of Bag-1 OE cells (S2B and S2C Fig).

### Knockout of Bag-1 altered RTKs in MCF-7 cells

**Fig 3:**
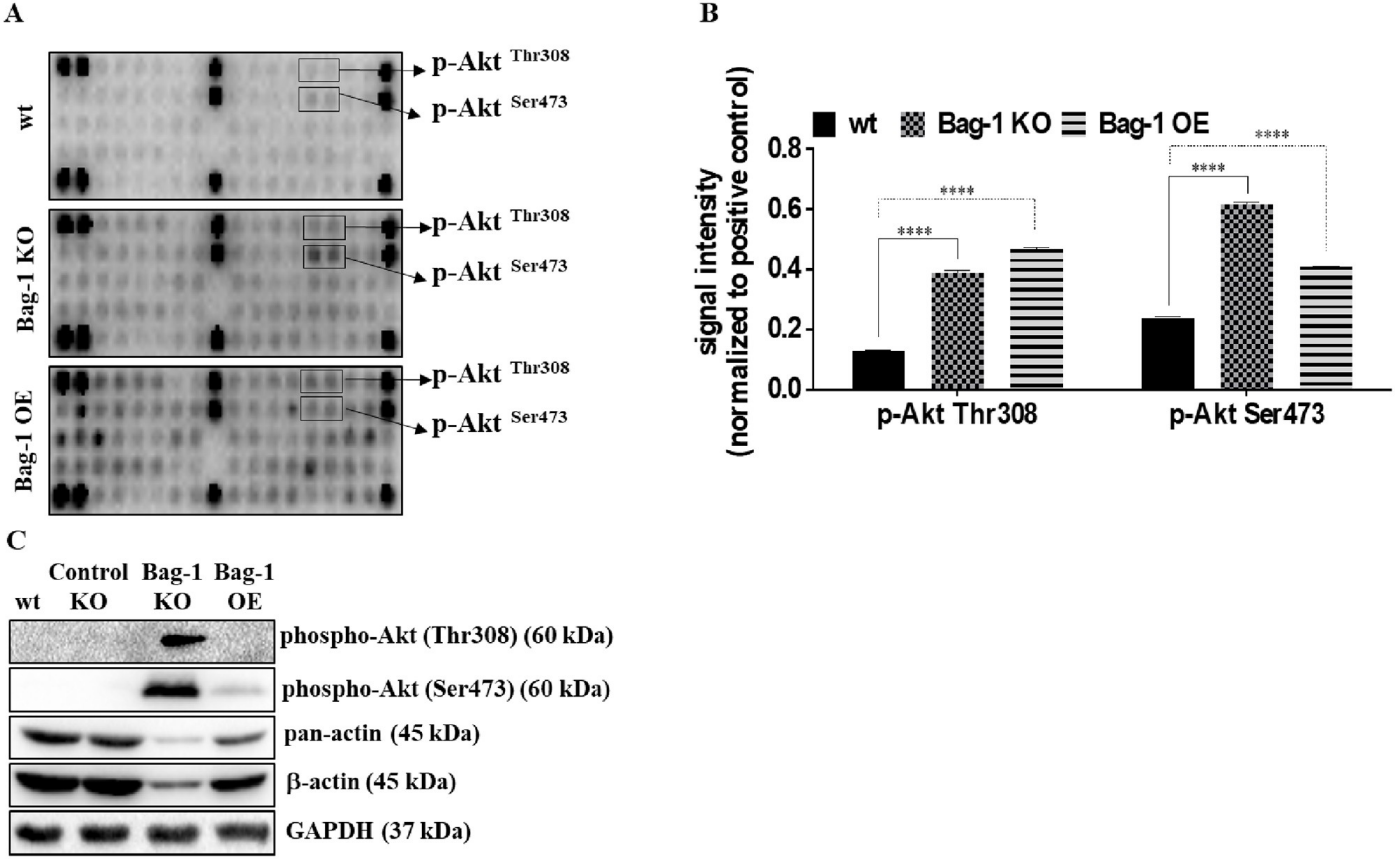
Expression profiles of phosphorylated RTKs and signaling routes in MCF-7 wt, Bag-1 KO and Bag-1 OE cells. A) The representative image of PathScan^®^ RTK Signaling Antibody Array panel that showing the expression of activated RTKs in wt, Bag-1 KO and Bag-1 OE MCF-7 cells. B) The bar graphs showed the quantification of the signal intensity of phospho-Akt Ser 473 and phospho-Akt Thr308 proteins. The mean ± SD of three repetitive experiments were analyzed by Two-way ANOVA, Tukey’s multiple comparison tests (**** p<0.0001). C) Confirmation of selected phosphorylated protein expression by immunoblotting assay. GAPDH was used as a loading control. The relative densitometry analysis represented the mean ± SD of three independent experiments. (*** p = 0.0003, **** p< 00001 by Two-way ANOVA, Tukey’s multiple comparison test, S3B Fig).

Considering the important roles of RTKs in breast cancer development, we investigated the effect of Bag-1 expression on the phosphorylation status of RTKs in MCF-7 cells by using Pathscan RTK Signaling Antibody Array kit (Fig 3A). Various RTKs and related signaling pathway members were increased in both Bag-1 KO and Bag-1 OE cells compared to that of wt MCF-7 cells (S3A Fig). Normalization of signals showed that phospho-Akt Thr308, phospho-Akt Ser473 were the most altered targets in Bag-1 KO cells compared to that of wt MCF-7 cells (**** p<0.0001) (Fig 3B). The expression profile of phospho-Akt Ser473 and phospho-Akt Thr308 were highly increased (**** p<0.0001) in Bag-1 KO cells compared to that of wt MCF-7 cells. Due to the β-actin (*** p = 0.0003) and pan-actin (**** p<0.0001) downregulation in Bag-1 KO cells, GAPDH was selected as a loading control (Fig 3C).

### Knockout of Bag-1 caused stress-mediated Akt activation and mesenchymal transition through dysregulated actin network

**Fig 4:**
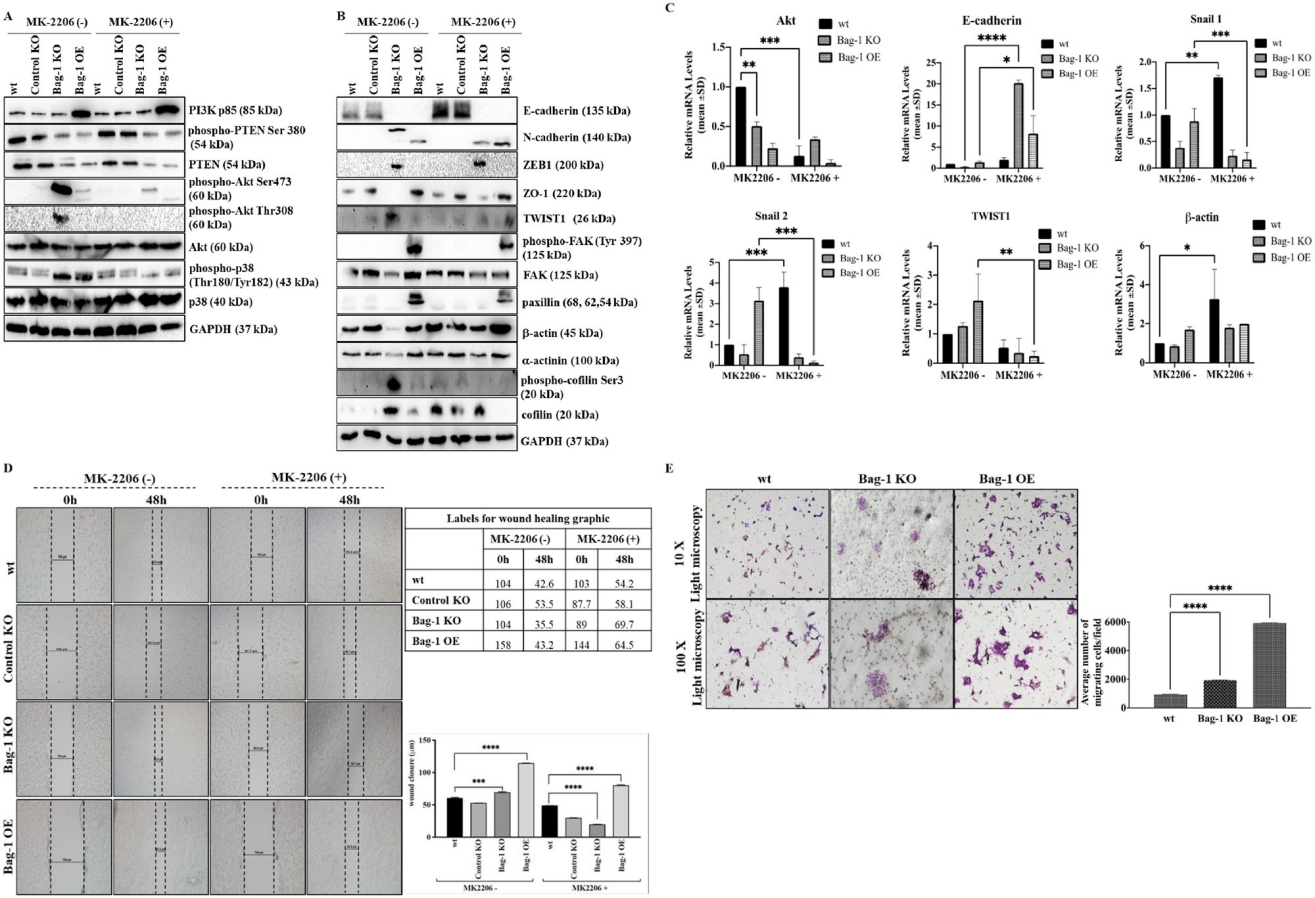
Investigation of the role of different Bag-1 expression levels on Akt, EMT, and focal adhesion pathways in MCF-7 cells, in association with their migration and invasion potential. Expression profiles of Akt signaling pathway members (A) and EMT, focal adhesion kinase pathway members (B) were investigated after MK-2206 treatment in MCF-7 wt, Control KO, Bag-1 KO and Bag-1 OE cells determined by immunoblotting assay. Densitometry analyses were shown in S4D and S4E Fig. The relative densitometry analysis represented the mean ± SD of three independent experiments. GAPDH was used as a loading control. (*p = 0.0114, **p = 0.0013, ***p = 0.0006, ****p<0.0001 by Two-way ANOVA, Tukey’s multiple comparison test). C) The transcriptional level of Akt, E-cadherin, Snail1, Snail2, TWIST1, and β-actin were detected in qRT-PCR analysis. All data collected from three independent experiments (Akt ** = 0.0040, ***p = 0.0002, E-cadherin ****p<0.0001, *p = 0.0317, Snail1 **p = 0.0021, ***p = 0.0007, Snail2 ***p = 0.0002, TWIST1 **p = 0.0089, β-actin *p = 0.0114. The designed primer sequences were shown in S2 Table. D) The effect of Bag-1 expression on cell migration of MCF-7 cells was assessed by wound healing assay, which was repeated by two-independent assays. Wound closure was imaged by light microscopy (magnification 4x, scale 10 μm), and analyzed by Two-way ANOVA, Tukey’s multiple comparison test, **** p< 00001. E) The invasion capacities of wt, Bag-1 KO and Bag-1 OE cells were determined by transwell migration assay. Left panel, invading cells (magnification x10 and x100); right panel, quantification on average number of migrating wt, Bag-1 KO and Bag-1 OE cells per well (****p<0.0001), counted by Image J and analyzed by One-way ANOVA, Dunnet’s multiple comparison test. The counted cell population was shown in S4H Fig as red stained dots.

Considering the significant Akt phosphorylation of both Ser473 and Thr308 residues in Bag-1 KO cells, we assessed the expression profile of upstream and downstream members of Akt, EMT and cytoskeletal proteins through MK-2206, a potent Akt inhibitor (Fig 4). The cell viability assay in wt, Control KO, Bag-1 KO and Bag-1 OE MCF-7 cells showed slight decrease in the presence of MK-2206 (S4A Fig). These results were confirmed by clonogenic cell survival analysis (S4B Fig). Considering the expression profiles of upstream and downstream members of Akt, PI3K expression was significantly higher in Bag-1 OE cells (****p<0.0001) compared to wt, Control KO and Bag-1 KO cells. Akt inhibition did not significantly alter Bag-1-mediated PI3K expression. phosphorylated PTEN and total PTEN and expression levels were downregulated in Bag-1 KO and Bag-1 OE cells (**** p<0.0001, ** p = 0.0028) and Akt inhibitor treatment did not change functional PTEN activity in all cell models. Similarly, Akt inhibition did not lead to expressional alterations at the total Akt level in the cells (** p = 0.0056), but MK-2206 prevented hyperphosphorylation of Akt at Ser473 and Thr308 in Bag-1 KO cells (**** p<0.0001). We confirmed that Akt inhibitor treatment led to significant Akt dephosphorylation in all cell lines. To examine the relationship between Bag-1 expression profile and cellular stress-response in MCF-7 cells, we investigated the p38 expression and found that phospho-p38 Thr180/Tyr182 was significantly increased in Bag-1 KO and Bag-1 OE (**** p< 0.0001) cells compared to that of Control KO and wt MCF-7 cells, which was suppressed by Akt inhibition (Fig 4A). We also confirmed that the Bag-1 KO cells are viable in increased stress conditions through detecting the cellular Reactive Oxygen Species (ROS) levels, which is one of the main stress indicators in the cells (S4C Fig).

We also investigated the Akt-mediated EMT and actin regulation of Bag-1 KO cells by immunoblotting assay (Fig 4B). The epithelial marker E-cadherin expression was upregulated in MK-2206 treated wt and Control KO cells (**** p<0.0001), whereas dramatically downregulated in both Bag-1 KO and Bag-1 OE cells regardless of Akt activation. The mesenchymal marker N-cadherin expression was upregulated in Bag-1 KO and Bag-1 OE cells (**** p<0.0001). Although the N-cadherin 140 kDa fragment was detected in Bag-1 OE cells, the N-cadherin profile was above normal band profiles in Bag-1 KO cells. This band profile indicated an inactive form of N-cadherin due to its pro-domain [21]. Following MK-2206 treatment, N-cadherin expression was downregulated in Bag-1 KO cells. The EMT signaling transcription factor ZEB1 expression was significantly enhanced in Bag-1 KO cells (**** p<0.0001) compared to Control KO cells. On the contrary, the tight junction protein ZO-1 was downregulated in Bag-1 KO cells, which was upregulated further by MK-2206 treatment (**** p<0.0001). The transcription factor TWIST1 was upregulated in untreated Bag-1 KO cells, whereas was downregulated by MK-2206 treatment (**** p<0.0001). phospo-FAK at Tyr397 expression was observed in Bag-1 OE cells and downregulated with MK-2206 treatment (**** p<0.0001). FAK expression level was significantly downregulated in Bag-1 KO cells (**** p<0.0001), yet Akt inhibitor treatment slightly altered FAK downregulation (* p = 0.0264). In contrast, MK-2206 treatment decreased FAK expression in Bag-1 OE cells compared to untreated Bag-1 OE (**** p<0.0001). Similar expression pattern was determined in paxillin expression (**** p<0.0001). Related to the cell cytoskeleton and EMT progression network, Akt activation dependent downregulation of β-actin and α-actinin (**** p<0.0001) were detected in Bag-1 KO cells, in which their expressions were enhanced through Akt inhibition. Phosphorylated cofilin level at Ser3 was significantly higher in Bag-1 KO cells compared to other cell models (**** p<0.0001) (Fig 4B). Moreover, we examined the relative mRNA expression of E-cadherin, TWIST1, Snail1, Snail2, β-actin and Akt by qRT-PCR. MK-2206 treatment decreased the Akt mRNA expression in all cell models. E-cadherin mRNA expression increased in Bag-1KO and Bag-1 OE cells (* p = 0.0126, **** p<0.0001). The relative Snail 1 and Snail 2 mRNA expressions were lower in Bag-1 KO cells compared to wt and Bag-1 OE cells, and Akt inhibition further reduced their expressions (** p = 0.0021, *** p = 0.0002). TWIST1 mRNA expression was higher in Bag-1 KO and Bag-1 OE cells, whereas significantly decreased by inhibition of Akt (** p = 0.0089). β-actin mRNA level was increased through Akt inhibition in Bag-1 KO cells (* p = 0.0114) (Fig 4C). Considering the high activation of Akt and increased expression profiles of EMT markers in Bag-1 KO cells, the potential association of Bag-1 expression on cell migration was determined by performing a wound healing assay. Bag-1 KO was more effective to repair *in vitro* wound closure compared to wt and Control KO MCF-7 cells after 48 h growth of cells. Bag-1 OE cells showed significant wound closure in 48 h. Akt inhibition suppressed the migration of cells into wound area significantly in Bag-1 KO cells and other cell models (Fig 4D). Time-dependent (0–24 and 48 h) wound healing images were captured under light microscopy and fluorescence microscopy after staining with DiOC6 (S4F and S4G Fig). We suggest that knockout of Bag-1 enhanced the migration potential of MCF-7 cells. Therefore, we also investigated the invasion potential of MCF-7 cells with different Bag-1 expression levels using Boyden Chamber assay. Both wt, Bag-1 KO and Bag-1 OE cells were migrated into lower chamber, but Bag-1 OE cells showed higher migration capability compared to wt and Bag-1 KO cells (**** p<0.0001). It was noticeable that Bag-1 KO cells showed distinct morphology, smaller cell size, and colony-formed cell population compared to wt and Bag-1 OE cells (Fig 4E, S4H Fig). Considering our results we obtained that Bag-1 KO and Bag-1 OE cells showed mesenchymal phenotype, but Bag-1 KO condition caused the focal adhesion decrease and actin remodeling compared to the other cell models. We also confirmed in normal (N) and tumor (T) tissues from 8 breast cancer patients that the expression of α-actinin which is a focal adhesion and actin cytoskeleton marker did not change in the presence of Bag-1 expression, even though Akt is still upregulated and phosphorylated in breast cancer (S4I and S4J Fig).

### Re-gain of each Bag-1 isoform prevented mesenchymal transition-related to Akt activation in Bag-1 KO cells

**Fig 5:**
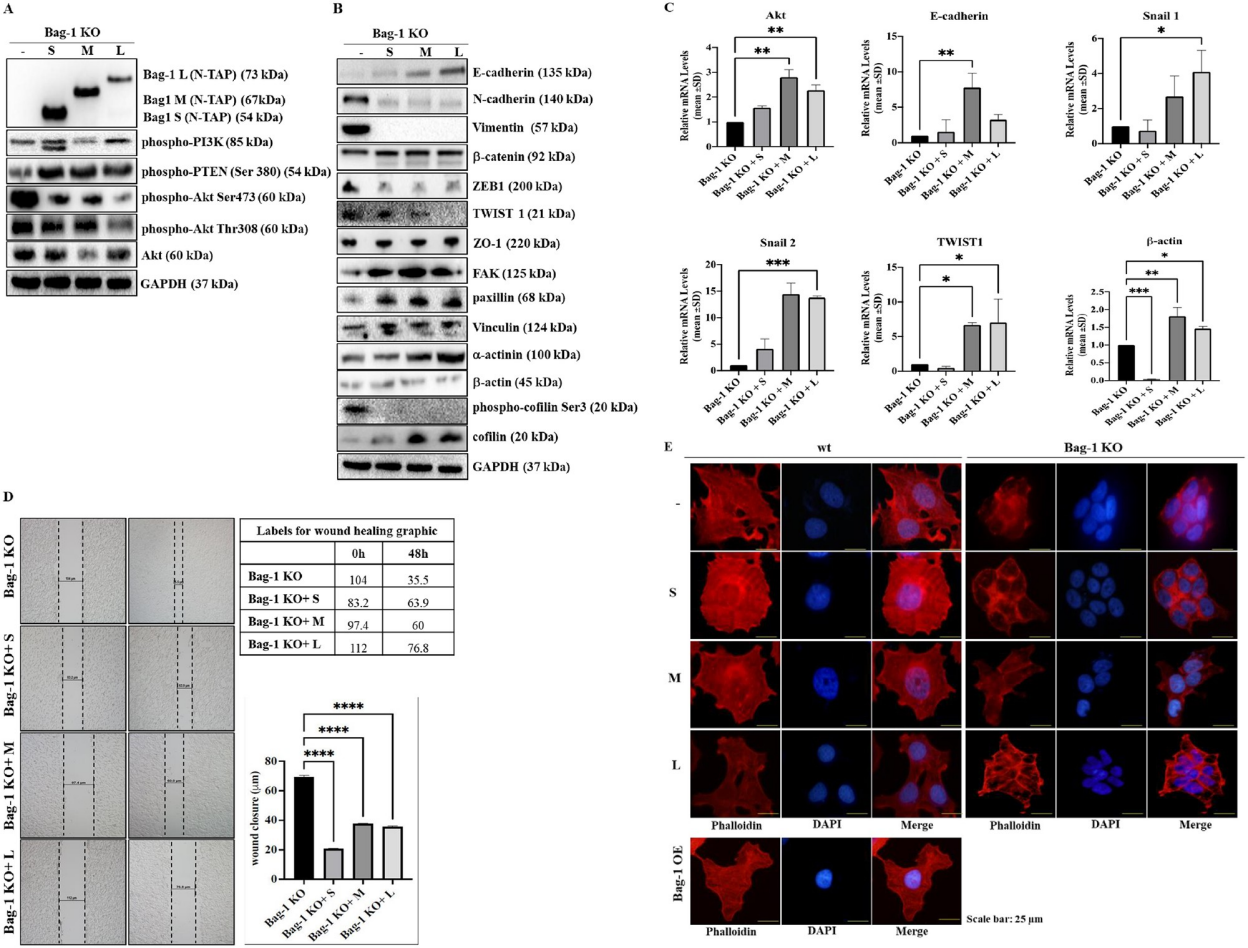
Bag-1 isoform-specific transfection of Bag-1 KO cells rescued cells from mesenchymal transition and supressed migratory roles. Expression profiles of Bag-1, and Akt signaling pathway members (A) and EMT, focal adhesion, actin cytoskeleton members (B) after Bag-1 isoform-specific transfections were analyzed by immunoblotting assay. Densitometry analyses were shown in S5B and S5C Fig. The relative densitometry analysis represented the mean ± SD of three independent experiments. **** p< 00001 by Two-way ANOVA, Tukey’s multiple comparison test. C) The mRNA levels of Akt, E-cadherin, Snail1, Snail2, TWIST1 and β-actin were detected in qRT-PCR analysis. (Akt ** = 0.0015, E-cadherin **p = 0.0031, Snail1 *p = 0.0207, Snail2 ***p = 0.0003, TWIST1 *p = 0.0187, β-actin *p = 0.0175, **p = 0.0015, ***p = 0.0007.D) The effect of Bag-1 isoform transfection into Bag-1 KO cells on cell migration of MCF-7 cells was assessed by wound healing assay, which was repeated by two-independent assays. Wound closure was imaged by light microscopy (magnification 4x, scale 10 μm), and analyzed by Two-way ANOVA, Tukey’s multiple comparison test, **** p< 00001. E) Visualization of the actin cytoskeleton in Bag-1 isoform transfected MCF-7 wt, Bag-1 KO and Bag-1 OE cells through fluorescent-labeled Phalloidin incubation by immunofluorescence experiment described in “Material and methods”. Scale bar is 25 μm and magnification is 64x.

In order to further investigate the potential role of Bag-1 in the EMT and cytoskeleton network, we transiently transfected Bag-1 KO cells with isoform-specific Bag-1 constructs. Then, we compared the relative cell viability of Bag-1 KO cells following Bag-1 S, M or L isoform transfection (S5A Fig). Immunoblotting results showed that the transfection of specific Bag-1 isoforms was successfully performed (**** p<0.0001). Phospho-PI3K expression was higher in Bag-1 KO+S cells (**** p<0.0001) but decreased in Bag-1 KO+M cells. Phospho-PTEN expression increased following the regain of Bag-1 isoforms and the highest expression was observed in Bag-1L isoform transfected cells (**** p<0.0001). In contrast, phospho-Akt Ser473 levels was downregulated after Bag-1 S, M, or L isoform transfections (**** p<0.0001). Similar expression profile was observed in phospho-Akt Thr308 (**** p<0.0001). Total Akt was downregulated in Bag-1M isoform transfected cells (*p = 0.0165) (Fig 5A). In Fig 5B, we investigated the expression profiles of EMT and actin-mediated cytoskeleton proteins. However, epithelial marker E-cadherin was significantly upregulated, mesenchymal marker N-cadherin and Vimentin was downregulated in Bag-1S, M or L transfection (**** p<0.0001). β-catenin expression did not show a significant change after S and M isoform transfection, but increased through Bag-1L transfection (*p = 0.0206). Transfection factors TWIST1 and ZEB1 were also downregulated after S, M or L isoform transfection in Bag-1 KO cells (**** p<0.0001). The tight junction protein ZO-1 expression did not show any significant change. Actin cytoskeleton-related focal adhesion proteins FAK, paxillin and Vinculin expressions were upregulated (**** p<0.0001) associated with α-actinin expression (**** p<0.0001) but the β-actin expression did not show significant change. Actin-binding protein cofilin phosphorylation level was decreased (**** p<0.0001), whereas total cofilin expression was upregulated following S, M and L isoforms transfection (**** p<0.0001) (Fig 5B). We also performed qRT-PCR assay, which revealed that the relative mRNA expression levels of Akt, E-cadherin, Snail1, Snail2, TWIST1 and β-actin was increased following S, M and L isoforms transfection (Fig 5C). Considering the role of re-gaining of Bag-1 isoforms into Bag-1 KO cells decreased the mesenchymal properties of cells, we investigated the role of isoform transfection on migration in Bag-1 KO cells. The wound-healing assay showed that isoform-specific transfection of Bag-1 limited the migratory roles of Bag-1 KO cells (Fig 5D). Time-dependent (0–24 and 48 h) wound healing images were captured under light microscopy and fluorescence microscopy after staining with DiOC6 (S5D Fig).

Considering the increased expression profile on focal adhesion and actin cytoskeleton proteins in Bag-1 KO cells through S, M and L isoform transfection, we investigated the actin organization in all cell models (untransfected wt, Bag-1 KO and Bag-1 S, M and L isoform transfected wt, Bag-1 KO and Bag-1 OE cells). To examine the effect of different Bag-1 expression levels on actin cytoskeleton regulation, generated MCF-7 cell models were stained with FITC-Phalloidin to mark F-actin and were visualized by immunofluorescence imaging. Untransfected wt, isoform-specific Bag-1 transfected wt and Bag-1 OE cells showed increased F-actin accumulation in the cell, whereas knockout of Bag-1 caused cytoplasmic condensation with the decrease in F-actin stress fiber. Bag-1 S, M and, L isoform transfected Bag-1 KO cells showed slightly more phalloidin stain compared to untransfected Bag-1 KO cells. Nuclear formation of cells was visualized by DAPI staining (Fig 5E- S5E Fig).

## Discussion

The multifunctional Bag-1 protein interacts with several molecular targets to regulate cell survival, proliferation and apoptosis. Several studies explained the anti-apoptotic potential and survival-targeted role of Bag-1 expression in cancer, whereas the effect of complete knockout of Bag-1 in breast cancer has not been revealed in depth yet. In the previous studies, complete loss or ablation of Bag-1 expression was found to be associated with the cell death of neuronal precursor cells and overexpression of Bag-1 was found to associated with the cell death of neural cells [22]. In a recent study, CRISPR/Cas9-mediated double Bag-1 KO mouse embryonic stem cells (Bag-1 KO-mES), which were generated by the deletion of exon 2 in Bag-1 gene showed high level of alkaline phosphatase with ES-specific dome shape morphology and maintenance of their pluripotent stemless properties with expressing Oct4, Sox2 and Nanog stem cell markers [23]. In a similar way, we generated Bag-1 KO MCF-7 (Fig 1) cells via editing of exon 2, 3 and 6 in the Bag-1 gene to elucidate the molecular consequences of the survival function of Bag-1. The proliferation experiments showed that Bag-1 KO MCF-7 cells were not capable of adhering within 24 h and their known 29 h doubling time, which was delayed to 48 h (Fig 2). Although we observed in our previos study that transient Bag-1 silencing attenuates the survival-related PI3K/Akt/mTOR signaling axis [9], quite interestingly in this study, Bag-1 knockout induced phosphorylation of Akt on Ser473 and Thr308 and led to its activation in MCF-7 cells (Fig 3). Akt has been indicated to be hyperactivated as an energy producer for the survival of cancer cells under increased ROS-mediated metabolic stress conditions [24]. Bag-1 was also found to prevent cellular ROS generation and stress-mediated growth inhibition in breast cancer cells [13]. In our study, it was observed that p38 phosphorylation and ROS generation was significantly increased in Bag-1 KO cells (Fig 4A). Thus, we have suggested that the significant Akt activation in Bag-1 KO cells is due to ROS generation caused by Bag-1 deprivation and may be a cause of survival of Bag-1 KO cells. To overcome Akt-mediated survival of Bag-1 KO MCF-7 cells, wt, Control KO, Bag-1 KO and Bag-1 OE cells were exposed to Akt inhibitor (MK-2206).

Bag-1 KO and Bag-1 OE MCF-7 cells showed a significant reduction in both total and phosphorylated PTEN levels independent from Akt inhibition (Fig 4A). Active PTEN co-localizes with β-catenin/E-cadherin at cell-cell junctions for the maintenance of tissue integrity, and the loss of E-cadherin expression reduces PTEN, and caused the loss of growth control through inhibiting cell-cell contact [25]. We suggested that decreased expression levels of epithelial marker E-cadherin and PTEN in Bag-1 KO and Bag-1 OE cells were associated with motility potential of Bag-1 KO and Bag-1 OE MCF-7 cells. Upregulation of N-cadherin in Bag-1 KO and Bag-1 OE cells compared to wt and Control KO confirmed increased mesenchymal profile of these cells. The band pattern differences in N-cadherin between Bag-1 KO and Bag-1 OE were noticeable, which is related to the proteolytic processing of N-cadherin. N-cadherin with pro-region is synthesized in the endoplasmic reticulum where p120^ctn^ binds to its cytoplasmic domain. Then, the cytoplasmic domain is phosphorylated by casein kinase II which leads to its binding to β-catenin, α-catenin and α-actinin. During the maturation process, the pro-region is cleaved by furin protease, and N-cadherin-catenin complex transported to the plasma membrane to link with actin cytoskeleton which regulates the cell-cell adhesion [21]. Thus, we conclude that hyperactivation of Akt led to immature form of N-Cadherin in Bag-1 KO cells via inhibiting its pro-region cleavage, whereas Akt inhibition was associated with the cleavage of N-cadherin from its pro-region, leading to an active N-cadherin. EMT is regulated by several transcription factors: Snail, Slug, TWIST, ZEB1 [26]. Increased ZEB-1 and TWIST expression confirmed the mesenchymal characteristics of Bag-1 KO cells. Bag-1 KO and Bag-1 OE cells differ in ZO-1 expression which is tight junction protein in EMT. Loss of ZO-1 expression deregulates actin cytoskeleton remodeling and suppresses the E-cadherin-mediated cell-cell adhesion [27, 28]. Therefore, in the induction of EMT in Bag-1 KO and Bag-1 OE cells, cell adherence function differs from Bag-1 OE cells in that Bag-1 KO cells, which tends to lose cell adherence. Focal adhesion proteins have a role in the E-cadherin-mediated cell-cell adherence junctions through linking to actin cytoskeleton [29]. In addition to the significant downregulation of focal adhesion protein FAK, β-actin, and α-actinin in Bag-1 KO cells compared to Bag-1 OE cells, the occurrence of FAK phosphorylation on Try 397 and paxillin expression in only Bag-1 OE cells confirmed our suggestion that Bag-1 KO cells lose their cell adherence characteristics. In Bag-1 KO cells, we also suggested that the E-cadherin/β-catenin complex was dissociated and therefore the adhesome complex did not form and link to the actin cytoskeleton due to the significant FAK, β-actin and α-actinin ablation in Bag-1 KO cells [30]. Inhibition of Akt in Bag-1 KO MCF-7 cells caused an increase in FAK, β-actin, and α-actinin expression levels. Therefore, Akt-mediated rearrangement of actin cytoskeleton network forms its functional structure in the presence of Akt inhibitor in Bag-1 KO MCF-7 cells. Reorganization of the cytoskeleton through Akt inhibition in Bag-1 KO cells is also confirmed by the alternation of phosphorylation of cofilin at Ser3. It is indicated that phosphorylation of cofilin at Ser3 inactivates its function by severing F-actin and promotes actin polymerization [31]. Akt inhibitor decreased the phospho-cofilin level at Ser3 in Bag-1 KO cells which led to the polymerization of F-actin (Fig 4B). Recovery of Bag-1 isoforms (Bag-1S, M, and L) one by one led to upregulation in PTEN, E-cadherin, FAK, paxillin, Vinculin, α-actinin and total cofilin expression in Bag-1 KO MCF-7 cells, allowed the suppression of EMT and induced the cell-cell adhesion potential of cells Besides these results, downregulation of N-cadherin, TWIST1, and ZEB1 were remarkable signatures of the relation between Bag-1 expression and EMT suppression in rescued MCF-7 cells. We found that regain of each Bag-1 isoform reduced phospho-Akt Ser473 levels compared to Bag-1 KO MCF-7 cells. Bag-1L isoform transfection showed significant decrease in phospho-Akt levels at both Ser473 and Thr308 residues (Fig 5A and 5B). ls. Accordingly, we proposed that Bag-1 overexpression enhances the tumorigenic potential, whereas knockout of Bag-1 causes a mesenchymal phenotype for breast cancer cells.

Due to the morphological changes of Bag-1 KO MCF-7 cells compared to wt, Control KO and Bag-1 KO cells, Bag-1 KO caused the redistribution of actin filaments (Fig 5E). The F-actin staining in Bag-1 KO cells was around the edges of cells and might be the outcome of colocalization of phospho-Akt, which is triggered by PIP3 signaling to increase cytoskeleton construction of cells [32]. Although the recovery of Bag-1 isoforms respectively resulted in the reorganization of F-actin tension fibers, it was not efficient for either wt or Bag-1 OE MCF-7 cells (Fig 5E) [33]. Loss of Bag-1 is substantial in Akt-mediated survival of the cells and causes remodelling of stress fibers and reducing of focal adhesions. Overexpression of Bag-1 was found to be associated with more metastatic character than knockout of Bag-1 with improved migration and wound healing properties (Fig 4D). Bag-1 loss showed higher migration rate compared to wt MCF-7 with their smaller cell size and colony-forming capabilities for survival resilience (Fig 4D). Regaining of each Bag-1 isoform limited the migration profile of Bag-1 KO cells (Fig 5D). All of these results highlighted that Bag-1 expression dependent alterations in MCF-7 breast cancer cells sharply changed the expression profile of cytoskeleton proteins, and affected EMT signalling. Therefore, Akt-dependent cytoskeletal regulatory potential is a novel understanding of the molecular role of Bag-1 in cell physiology.

## Conclusions

**Fig 6:**
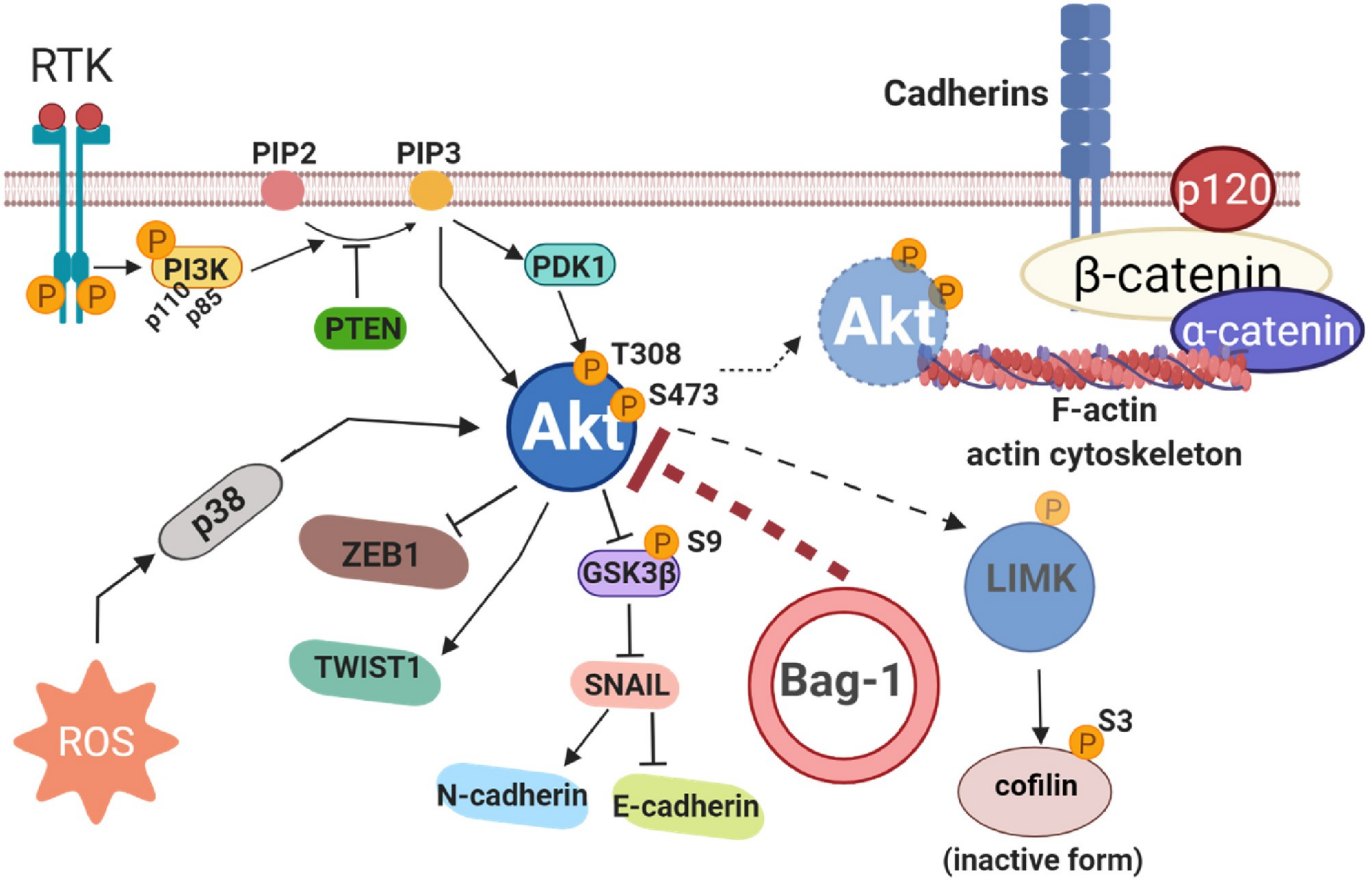
The schematic overview of Akt-mediated actin reorganization in Bag-1 deficiency. Akt is the main regulator of this signaling route, which was activated by ROS generation in Bag-1 KO MCF-7 cells and triggered several downstream mediators to enhance mesenchymal characteristics and cytoskeleton rearrangement. The expression profile of Bag-1 is important for the Akt-mediated cytoskeleton reorganization. This figure was created with BioRender.com.

Multifunctional Bag-1 possesses a central role in the survival-related signaling axis to maintain normal cell physiology. The enhanced stress conditions due to homozygous deletion of Bag-1 gene hyperactivated Akt with reduced PTEN expression to maintain cell survival with dysregulated mesenchymal cellular characteristics. The findings in this study highlighted the functional role of Bag-1 on the PI3K/Akt signaling axis, which orchestrates cell cytoskeleton targets in MCF-7 breast cancer cells (Fig 6). Stress-mediated Akt activation and reduced PTEN levels resulted in the reorganization of actin cytoskeleton and increased mesenchymal phenotype in Bag-1 KO MCF-7 breast cancer cells. Further studies are required to understand the molecular mediators of Akt hyperactivation in Bag-1 deficiency, which prevent pro-region processing of N-Cadherin. Exploring different Akt phosphorylation sites and their subcellular localizations related to cell survival and motility may be critical to understand Bag-1 deficiency problems in the cells. In conclusion, the generation of CRISPR/Cas9 mediated Bag-1 KO MCF-7 breast cancer cells provides deeper understanding of the possible regulatory role of Bag-1 for Akt in cellular dynamics related to cytoskeleton organization. Ectopic Bag-1 isoforms may enlighten the biological importance of each isoform in stress-mediated cellular responses.

## Supporting information

S1 Table **A**. Schematic representation of target sgRNA sequences for specific Bag-1 gene loci. Different sgRNA sequences of Bag-1 KO plasmid (sc-417179) were obtained from Santa Cruz and the regions were found from comparing the human nucleotides sequences through BLAST and also checked at Bag-1 genomic sequence (NG_029018.1). **B)** Designed primers to validate Bag-1 deficiency in MCF-7 cells.
(PDF)

S2 Table The sequences of PCR primer pairs used for E-cadherin, Snail1, Snail2, Twist1 and β-actin. (PDF)

S1 Fig A) The genomic PCR analysis of exon 2, exon 3, and exon 6 in Bag-1 KO clones and wt cells following standard PCR reaction with designed Guide A, B, and C primers. B) Sanger sequencing results of forward and reverse reads targeting exon 2 of Bag-1 in Bag-1 KO cells by Guide A PCR product. C) Sanger sequencing results of forward and reverse reads targeting exon 3 of Bag-1 in Bag-1 KO cells by Guide B PCR product. D) Sanger sequencing results of forward and reverse reads targeting exon 6 of Bag-1 in Bag-1 KO cells by Guide C PCR product. E) Densitometry analysis of Fig 1A. The relative densitometry analysis represented the mean ± SD of three independent experiments. (** p = 0.0044, *** p = 0.0005, **** p< 00001 by Two-way ANOVA, Tukey’s multiple comparison test.
(PDF)

S2 Fig A) The overexpression of Bag-1 was confirmed by the immunoblotting assay. Bag-1 OE cells were generated by the transfection of the TAP-tag Bag-1 expression vector into MCF-7 cells and the upper band profile showed the TAP-tag Bag-1L isoform. β-actin was used as the loading control. B) Morphological images of wt, Control KO, Bag-1 KO, and Bag-1 OE cells were obtained by light microscopy in a time-dependent manner. Magnification 40x, scale 10 μm. C) Colony-forming potentials of wt, Control KO, Bag-1 KO, and Bag-1 OE cells determined by Colony formation assay. The number of colonies represented the mean ± SD of three repetitive experiments and measured using Image J based on their densities and analyzed by Two-way ANOVA, Tukey’s multiple comparison test. Scale bar is 10 μm and magnification is 10x. (**, p = 0.0012, *** p = 0.0002).
(PDF)

S3 Fig A) The expression profiles of 39 different RTKs that were obtained by PathScan RTK assay. The signal intensity of these proteins was normalized to positive control and analyzed GraphPad Prism version 8, https://www.graphpad.com/. B) Densitımetry analysis of immunoblotting results of Fig 3C. The relative densitometry analysis represented the mean ± SD of three independent experiments. (*** p = 0.0003, **** p< 00001 by Two-way ANOVA, Tukey’s multiple comparison test).
(PDF)

S4 Fig A) The effect of 24 h treatment of 500 nM MK-2206 on cells was determined by MTT Cell Viability assay. The bar histograms represent the mean ± SD of three independent experiments with at least four replicates. (*** p = 0.0003, **** p< 00001 by Two-way ANOVA, Sidak’s multiple comparison test). B) The effect of MK-2206 on colony formation potentials of wt, Control KO, Bag-1 KO, and Bag-1 OE cells was determined by colony formation assay. The number of colonies represented the mean ± SD of three repetitive experiments, measured by Image J based on their densities and were analyzed by Two-way ANOVA, Tukey’s multiple comparison test. (**** p< 0.0001). C) Cellular ROS generation of wt, Control KO, Bag-1 KO, and Bag-1 OE cells was measured by DCFH-DA staining and analyzed by BD Accuri C6 flow cytometer (* p< 0.05 and ** p< 0.01, **** p< 0.0001). D) Densitometry analysis of Fig 4A. E) Densitometry analysis of Fig 4B. The relative densitometry analysis represented the mean ± SD of three independent experiments. GAPDH was used as a loading control. (*p = 0.0114, **p = 0.0013, ***p = 0.0006, ****p<0.0001 by Two-way ANOVA, Tukey’s multiple comparison test). F-G) Wound healing images of wt, Control KO, Bag-1 KO and Bag-1 OE cells with MK-2206 treatment in time dependent manner. H) Cell images of Boyden Chamber assay that was analyzed by ImageJ. Red dots showed the cells that counted as migrated cells. I-J) Immunoblotting (I) and densitometry analysis (J) of the expression profiles of phospho-Akt Ser473, total Akt and α-actinin in different breast tissue subtypes.
(PDF)

S5 Fig A) The cell viability of Bag-1 KO cells and transfected with Bag-1S, M and L isoforms, respectively was determined by MTT Cell Viability assay. B-C) Densitometry analyses of immunoblotting results of Bag-1 KO cells and transfected with Bag-1S, M and L isoforms. **** p< 00001 by Two-way ANOVA, Tukey’s multiple comparison test). D) Time-dependent wound healing images of Bag-1 KO cells and transfected with Bag-1S, M and L isoforms. E) Visualization of the actin cytoskeleton in Bag-1 isoform plasmid transfected MCF-7 wt and Bag-1 KO cells through fluorescent-labeled Phalloidin incubation by immunofluorescence experiment described in “Material and methods”.
(PDF)

S1 Raw images (PDF)
