# Supplementary material for: CRISPR/Cas9-mediated Bag-1 knockout increased mesenchymal characteristics of MCF-7 cells via Akt hyperactivation-mediated actin cytoskeleton remodeling
Source: PLoS One. 2022 Jan 7;17(1):e0261062. doi: 10.1371/journal.pone.0261062 (PMC8741009; doi:10.1371/journal.pone.0261062)
Supplement: S1 Table — A. Schematic representation of target sgRNA sequences for specific Bag-1 gene loci. Different sgRNA sequences of Bag-1 KO plasmid (sc-417179) were obtained from Santa Cruz and the regions were found from comparing the human nucleotides sequences through BLAST and also checked at Bag-1 genomic sequence (NG_029018.1). B) Designed primers to validate Bag-1 deficiency in MCF-7 cells. (PDF) [file pone.0261062.s001.pdf]

Table S1A

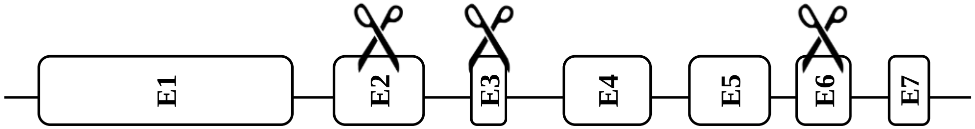

Table S1 B

**Primers for Bag-1 KO**				
**Name**	**Target region**	**gRNA sequences**	**Primer sequences**	**Expected PCR product size**
Guide A	Exon 2	TGAACCAGTTGTCCAAGACC	**F:** TTTCTTTCTCCCCAGGCAAT<br>**R:** TGTGGAACCCCTATGACCTC	118 bp
Guide B	Exon 3	GGTTGCCGGGTCATGTTAAT	**F:** GGAAACACCGTTGTCAGCAC<br>**R:** ACAGGAGCAGGAGGGTATGA	252 bp
Guide C	Exon 6	AGGCTTGGTAAAAAAGGTTC	**F:** ACAGGATACAGACCCAGGGA<br>**R:** ACCACGCTCCTACACTACCT	337 bp

**Primers for off-targets**			
**Name**	**Target region**	**Target gRNA**	**Primer sequences**
Off-target 1	CTD-2183H9.7|F8_chrX<br>chrX:154126403-154126425	gRNA A<br>Exon 2	F: TCGTCGGCAGCGTCAACACTGCAGCTGCGTATCT<br>R: GTCTCGTGGGCTCGGAGAAGGCCTGAAGAGTGCAC
Off-target 2	AC007682.1|AC097463.2_chr2<br>Chr 2: 52130030-52130052	gRNA B<br>Exon 3	F: TCGTCGGCAGCGTCCAGTCAGTCATCTCAGAGAAGACA<br>R: GTCTCGTGGGCTCGGTGTGCTCTGCCAACTTTGTT
Off-target 3	RPL31P12|RNU6-1246P_chr1 –<br>Chr1:73056651-73056673	gRNA C<br>Exon 6	F: TCGTCGGCAGCGTCACCTGGCAATATTCTCAGCCA<br>R: GTCTCGTGGGCTCGGACATGAACTCTTCTGTGGCA
