# Supplementary material for: CRISPR/Cas9-mediated Bag-1 knockout increased mesenchymal characteristics of MCF-7 cells via Akt hyperactivation-mediated actin cytoskeleton remodeling
Source: PLoS One. 2022 Jan 7;17(1):e0261062. doi: 10.1371/journal.pone.0261062 (PMC8741009; doi:10.1371/journal.pone.0261062)
Supplement: S2 Table — (PDF) [file pone.0261062.s002.pdf]

Table S2

Target	Sequence
E-cadherin Forward	ATTTTTCCCTCGACACCCGAT
E-cadherin Reverse	TCCCAGGCGTAGACCAAGA
Snail1 Forward	ACTGCAACAAGGAATACCTCAG
Snail1 Reverse	GCACTGGTACTTCTTGACATCTG
Snail2 Forward	TGTGACAAGGAATATGTGAGCC
Snail2 Reverse	TGAGCCCTCAGATTTGACCTG
Twist1 Forward	GTCCGCAGTCTTACGAGGAG
Twist1 Reverse	GCTTGAGGGTCTGAATCTTGCT
β-actin Forward	AGAGCTACGAGCTGCCTGAC
β-actin Reverse	AGCACTGTGTTGGCGTACAG
