# Supplementary material for: CRISPR/Cas9-mediated Bag-1 knockout increased mesenchymal characteristics of MCF-7 cells via Akt hyperactivation-mediated actin cytoskeleton remodeling
Source: PLoS One. 2022 Jan 7;17(1):e0261062. doi: 10.1371/journal.pone.0261062 (PMC8741009; doi:10.1371/journal.pone.0261062)
Supplement: S1 Fig — A) The genomic PCR analysis of exon 2, exon 3, and exon 6 in Bag-1 KO clones and wt cells following standard PCR reaction with designed Guide A, B, and C primers. B) Sanger sequencing results of forward and reverse reads targeting exon 2 of Bag-1 in Bag-1 KO cells by Guide A PCR product. C) Sanger sequencing results of forward and reverse reads targeting exon 3 of Bag-1 in Bag-1 KO cells by Guide B PCR product. D) Sanger sequencing results of forward and reverse reads targeting exon 6 of Bag-1 in Bag-1 KO cells by Guide C PCR product. E) Densitometry analysis of Fig 1A. The relative densitometry analysis represented the mean ± SD of three independent experiments. (** p = 0.0044, *** p = 0.0005, **** p< 00001 by Two-way ANOVA, Tukey’s multiple comparison test. (PDF) [file pone.0261062.s003.pdf]

Fig S1 A

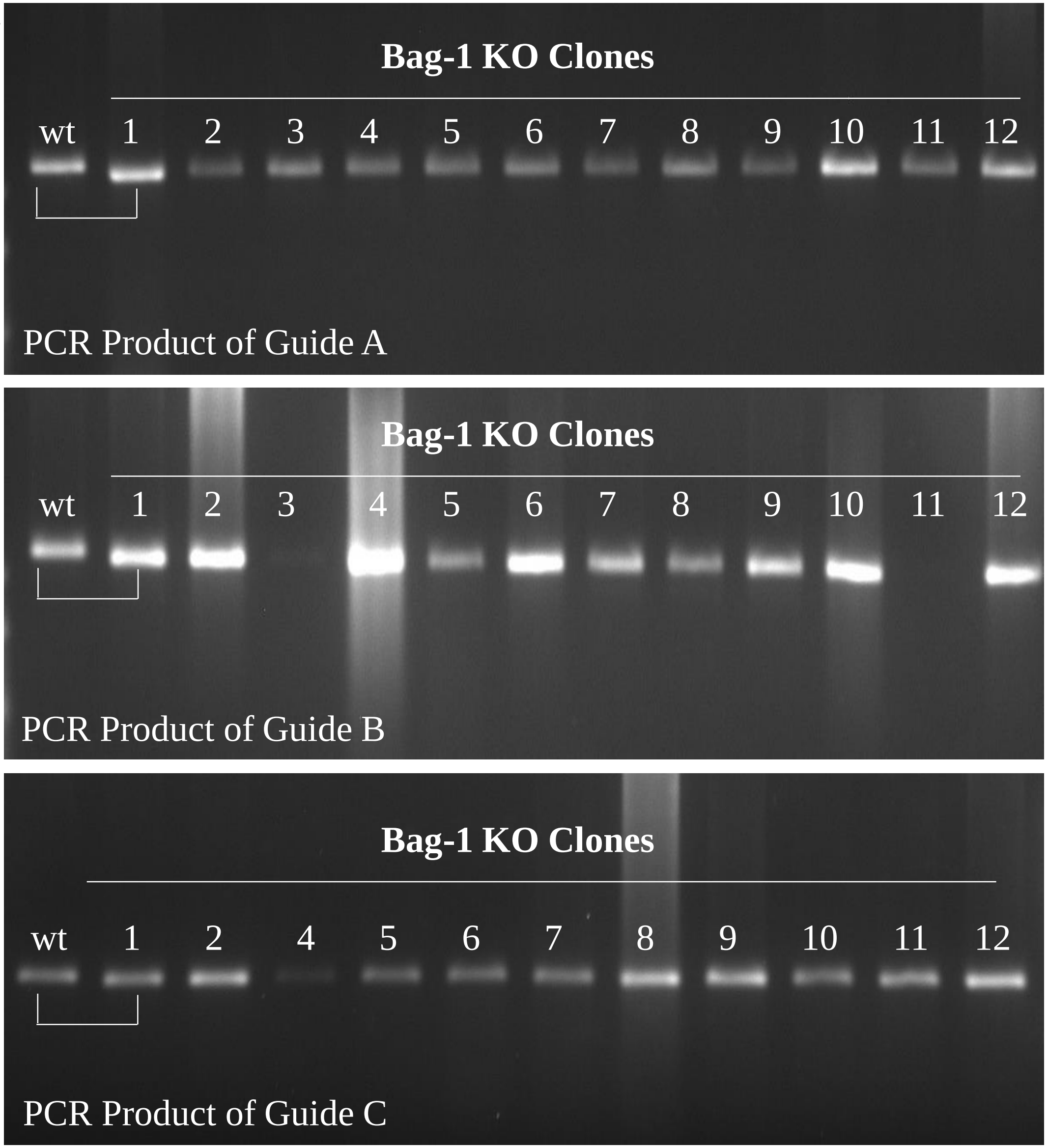
Bag-1 KO Clones
wt 1 2 3 4 5 6 7 8 9 10 11 12
PCR Product of Guide A
Bag-1 KO Clones
wt 1 2 3 4 5 6 7 8 9 10 11 12
PCR Product of Guide B
Bag-1 KO Clones
wt 1 2 4 5 6 7 8 9 10 11 12
PCR Product of Guide C

Fig S1 B

Bag-1 KO Guide A Forward Sequencing (targeting exon 2)

Target sequence PAM

wt 5’-TACCTCCCAGCAGGGCAGCAGTGAACCAGTTGTCCAAGACCTGGCCCAGGTTGTTGAAGAGGTCATAGGGG-3’

Bag-1 KO 5’-TAC--TCCCAGCAGGGCAGCAGTGAACCAGTTGTCCAAG--------------CCAGGTTGTTGAAGAGGTCATAGGGG-3’ **(-7 bp)**

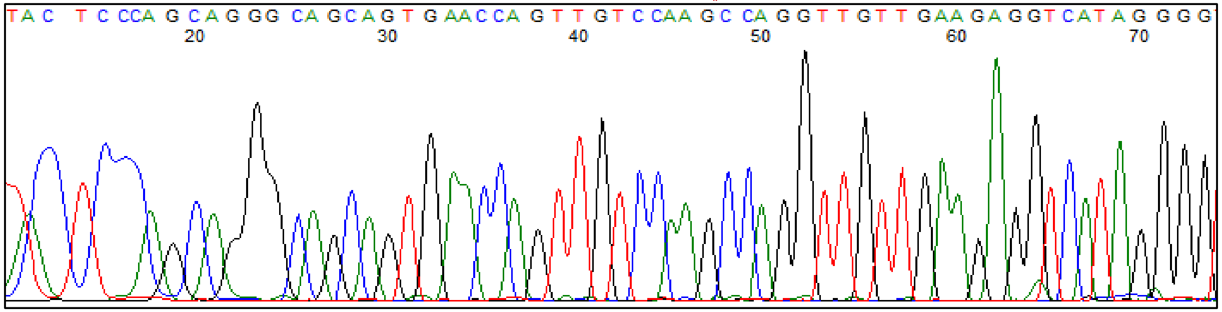

Bag-1 KO Guide A Reverse Sequencing (targeting exon 2)

Target sequence PAM

wt 5’-TACCTCCCAGCAGGGCAGCAGTGAACCAGTTGTCCAAGACCTGGCCCAGGTTGTTGAAGAGGTCATAGGGG-3’

Bag-1 KO 5’-TACCTCCCAGCAGGGCAGCAGTGAACCAGTT------C—AGACCTGGCC-AGGTTGTTGAAGAGGTCATAGGGG-3’ **(-6 bp)**

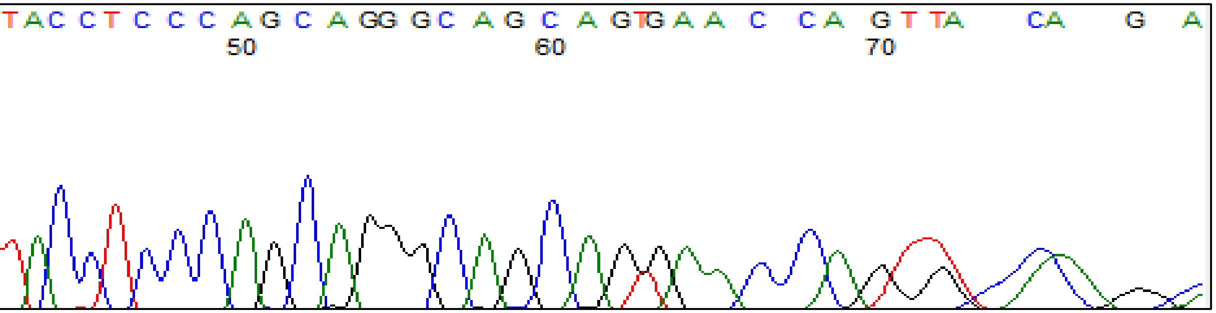

Fig S1 C

Bag-1 KO Guide B Forward Sequencing (targeting exon 3)

Target sequence PAM

wt 5’-ATGGTTGCCGGGTCATGTTAATTGGGAAAAAGGTAAATTGCTTTTTCCATCAGAAATCCTCAGAATCAAA-3’

Bag-1 KO 5’-ATGGTTGCCGGGTCATGTT--------GGGAAA-----GGTAAATTGCTTTTTCCATCAGAAATCCTCAGAATCAAA-3’ **(-6 bp)**

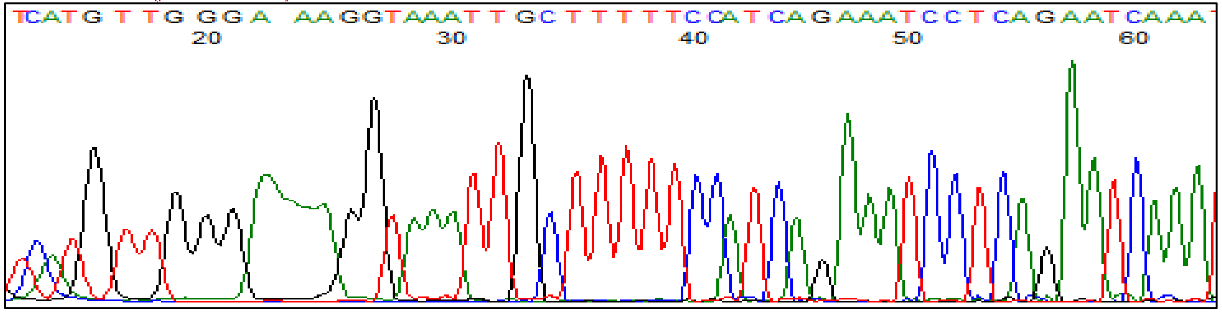

Bag-1 KO Guide B Reverse Sequencing (targeting exon 3)

Target sequence PAM

wt 5’-ATGGTTGCCGGGTCATGTTAATTGGGAAAAAGGTAAATTGCTTTTTCCATCAGAAATCCTCAGAATCAAA-3’

Bag-1 KO 5’-ATGGTTGCCGGGTCATGTT--------GGGAAAA---GGTAAATTGCTTTTTCCATCAGAAATCCTCAGAATCAAA-3’ **(-5 bp)**

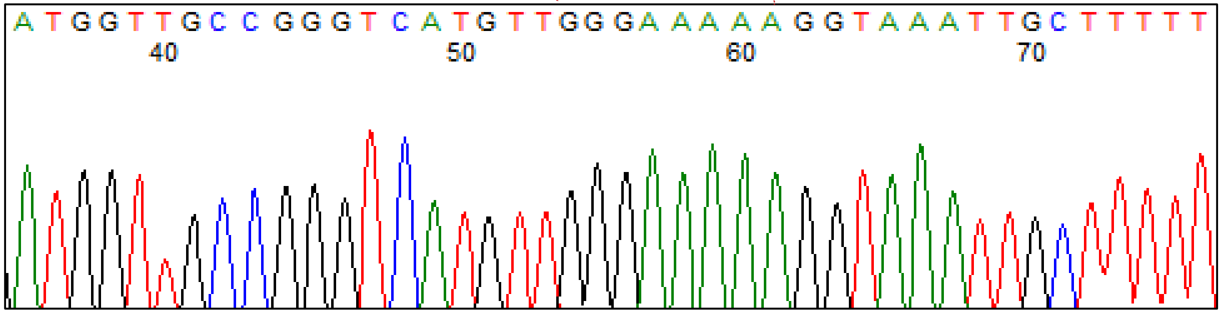

Fig S1 D

Bag-1 KO Guide C Forward Sequencing (targeting exon 6)

Target sequence PAM

wt 5’-TAGATTGAAAAGGAAAGGCTTGGTAAAAAAGGTTCAGGTGAGTTGTGTAATAGTGCACGACAAGGTGTAA-3’

Bag-1 KO 5’-TAGATTGAAAAGGAAAGGCTT--------------------------------GGTGAGTTGTGTAATAGTGCACGACAAGGTGTAA-3’**(-15bp)**

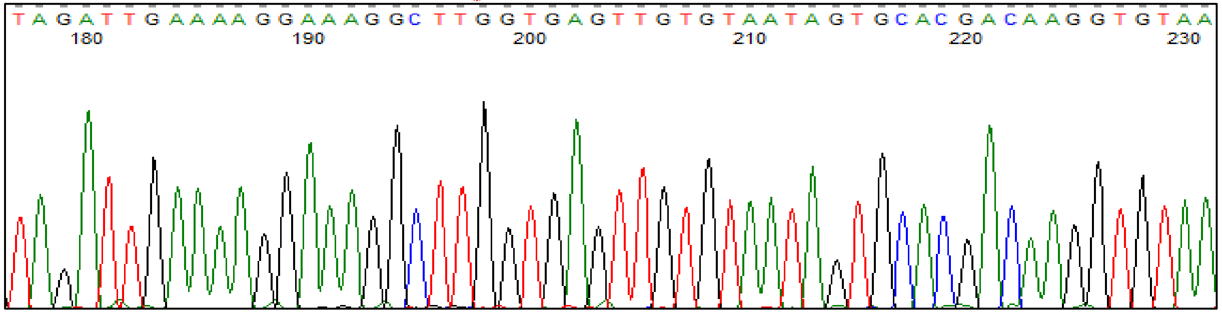

Bag-1 KO Guide C Reverse Sequencing (targeting exon 6)

Target sequence PAM

wt 5’-CAGTAGATTGAAAAGGAAAGGCTTGTTAAAAAAGGTTCAGGTGAGTTGTGTAATAGTGCACGACAAGGTG-3’

Bag-1 KO 5’-CAGTAGATTGAAAAGGAAAGGCTTGTT--------------------------------GAGTTGTGTAATAGTGCACGACAAGGTG-3’**(-15bp)**

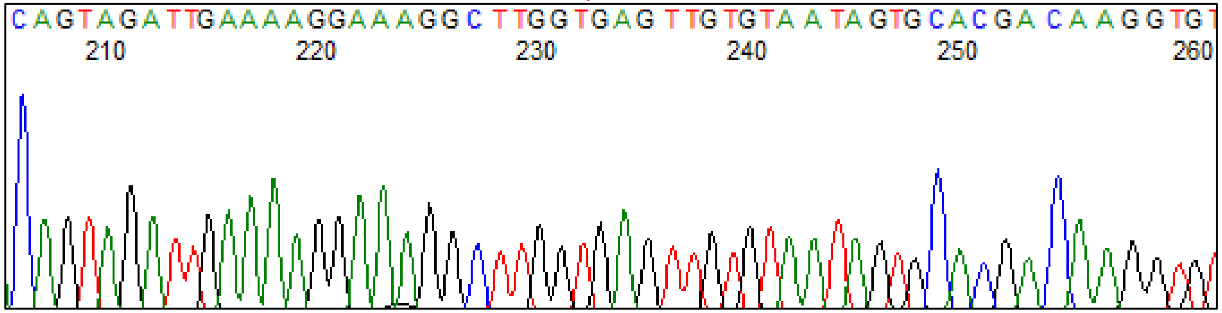

Fig S1 E

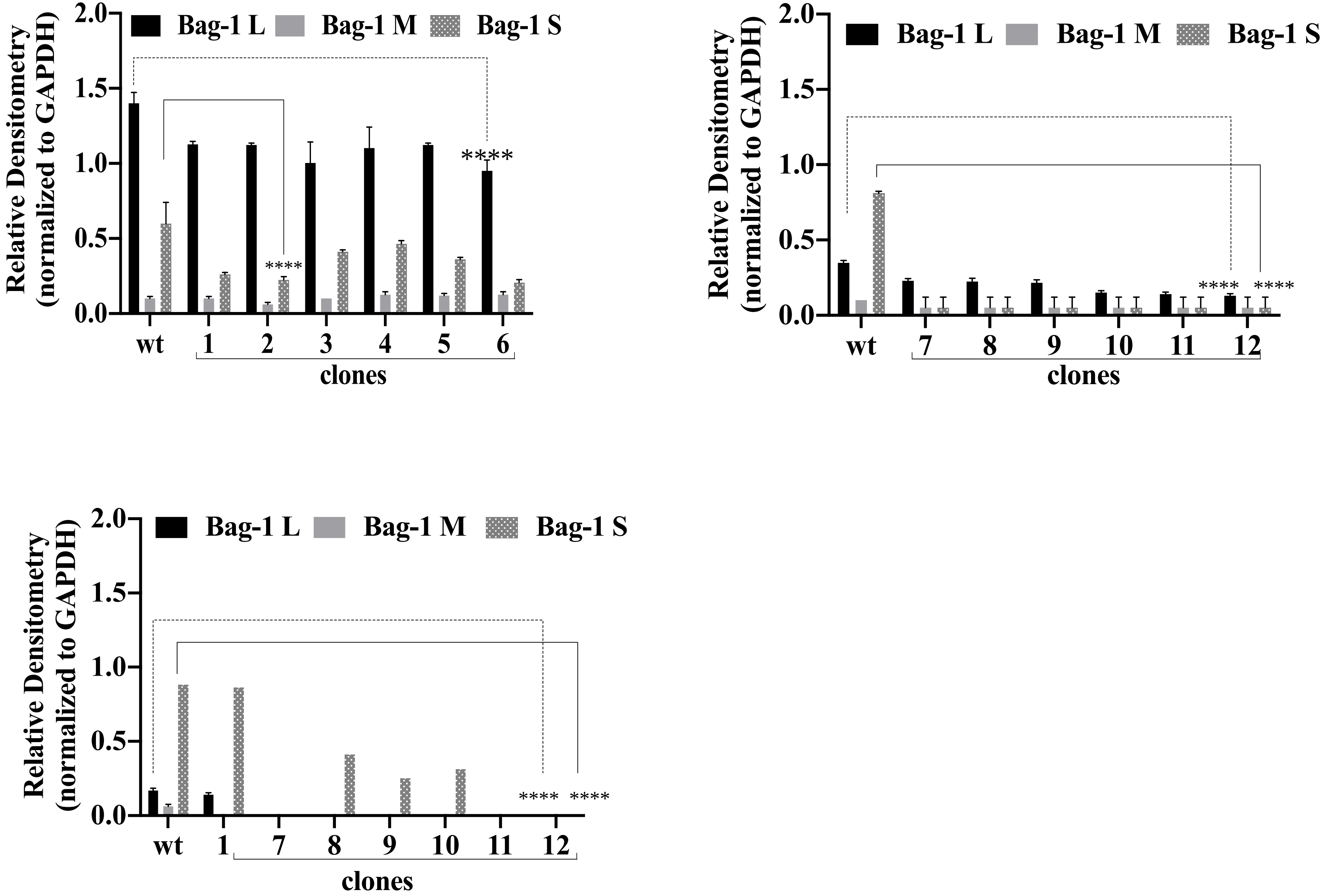
Bag-1 L
Bag-1 M
Bag-1 S
Relative Densitometry (normalized to GAPDH)
2.0
1.5
1.0
0.5
0.0
****
****
wt
1
2
3
4
5
6
clones
Bag-1 L
Bag-1 M
Bag-1 S
Relative Densitometry (normalized to GAPDH)
****
****
wt
7
8
9
10
11
12
clones
Bag-1 L
Bag-1 M
Bag-1 S
Relative Densitometry (normalized to GAPDH)
****
****
wt
1
7
8
9
10
11
12
clones
