# Supplementary figures and images for: CRISPR/Cas9-mediated Bag-1 knockout increased mesenchymal characteristics of MCF-7 cells via Akt hyperactivation-mediated actin cytoskeleton remodeling
Source: PLoS One. 2022 Jan 7;17(1):e0261062. doi: 10.1371/journal.pone.0261062 (PMC8741009; doi:10.1371/journal.pone.0261062)

**Fig S2 A**

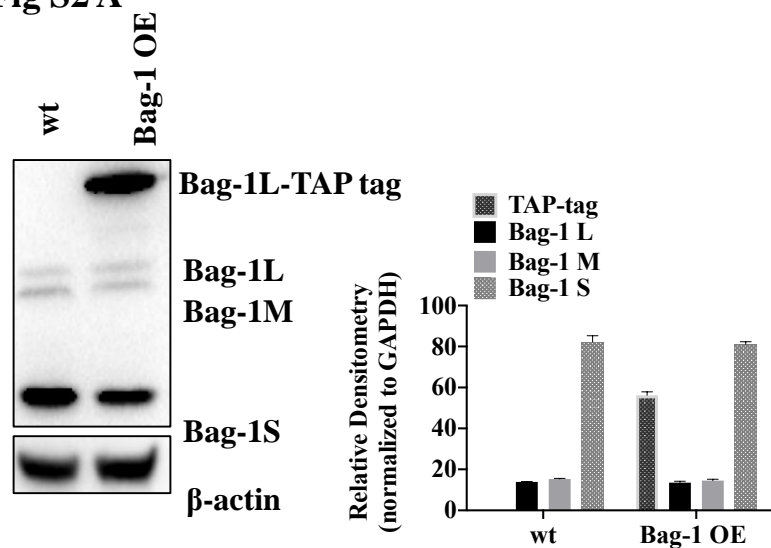

**B**

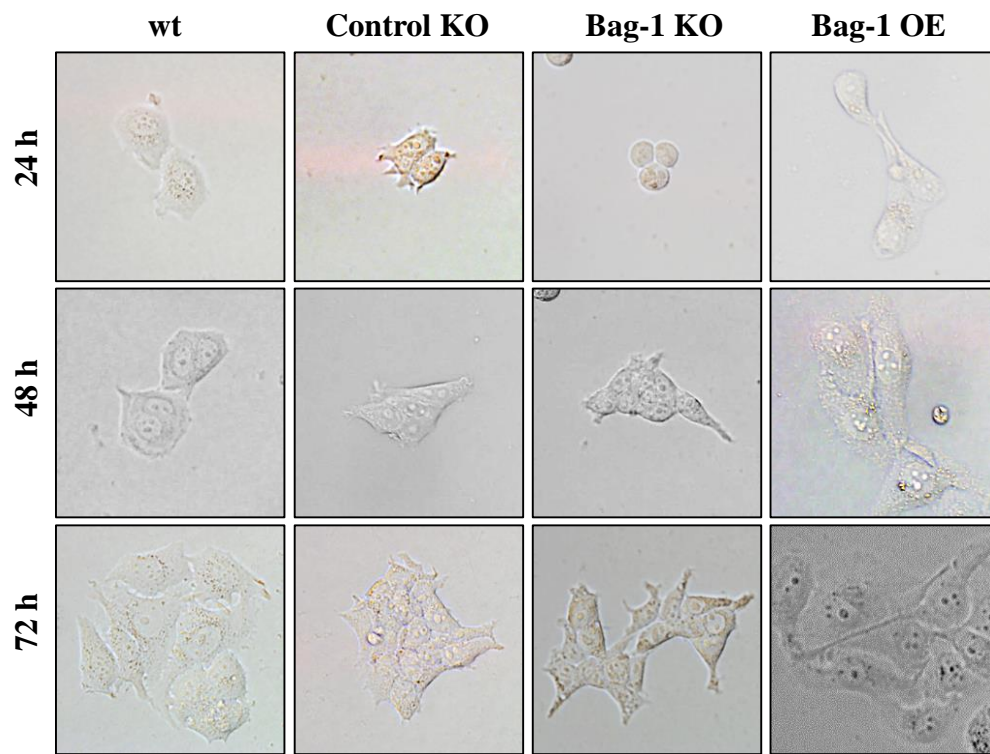

**C**

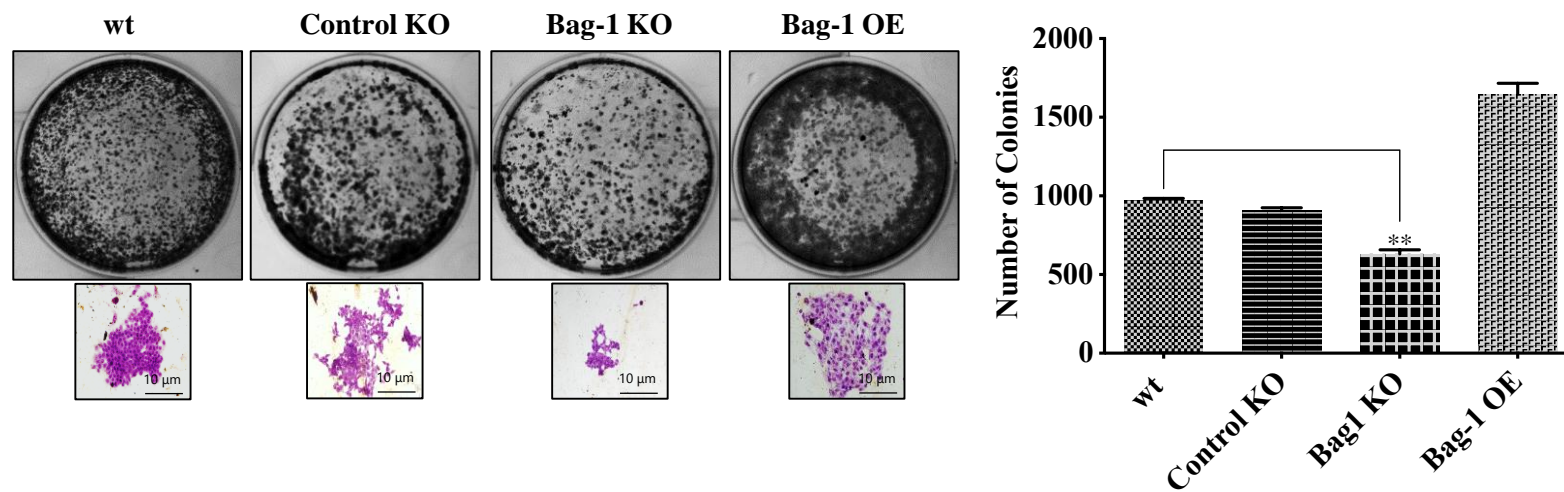

Supplement: S2 Fig — A) The overexpression of Bag-1 was confirmed by the immunoblotting assay. Bag-1 OE cells were generated by the transfection of the TAP-tag Bag-1 expression vector into MCF-7 cells and the upper band profile showed the TAP-tag Bag-1L isoform. β-actin was used as the loading control. B) Morphological images of wt, Control KO, Bag-1 KO, and Bag-1 OE cells were obtained by light microscopy in a time-dependent manner. Magnification 40x, scale 10 μm. C) Colony-forming potentials of wt, Control KO, Bag-1 KO, and Bag-1 OE cells determined by Colony formation assay. The number of colonies represented the mean ± SD of three repetitive experiments and measured using Image J based on their densities and analyzed by Two-way ANOVA, Tukey’s multiple comparison test. Scale bar is 10 μm and magnification is 10x. (**, p = 0.0012, *** p = 0.0002). (PDF) [file pone.0261062.s004.pdf]

Fig S3 A

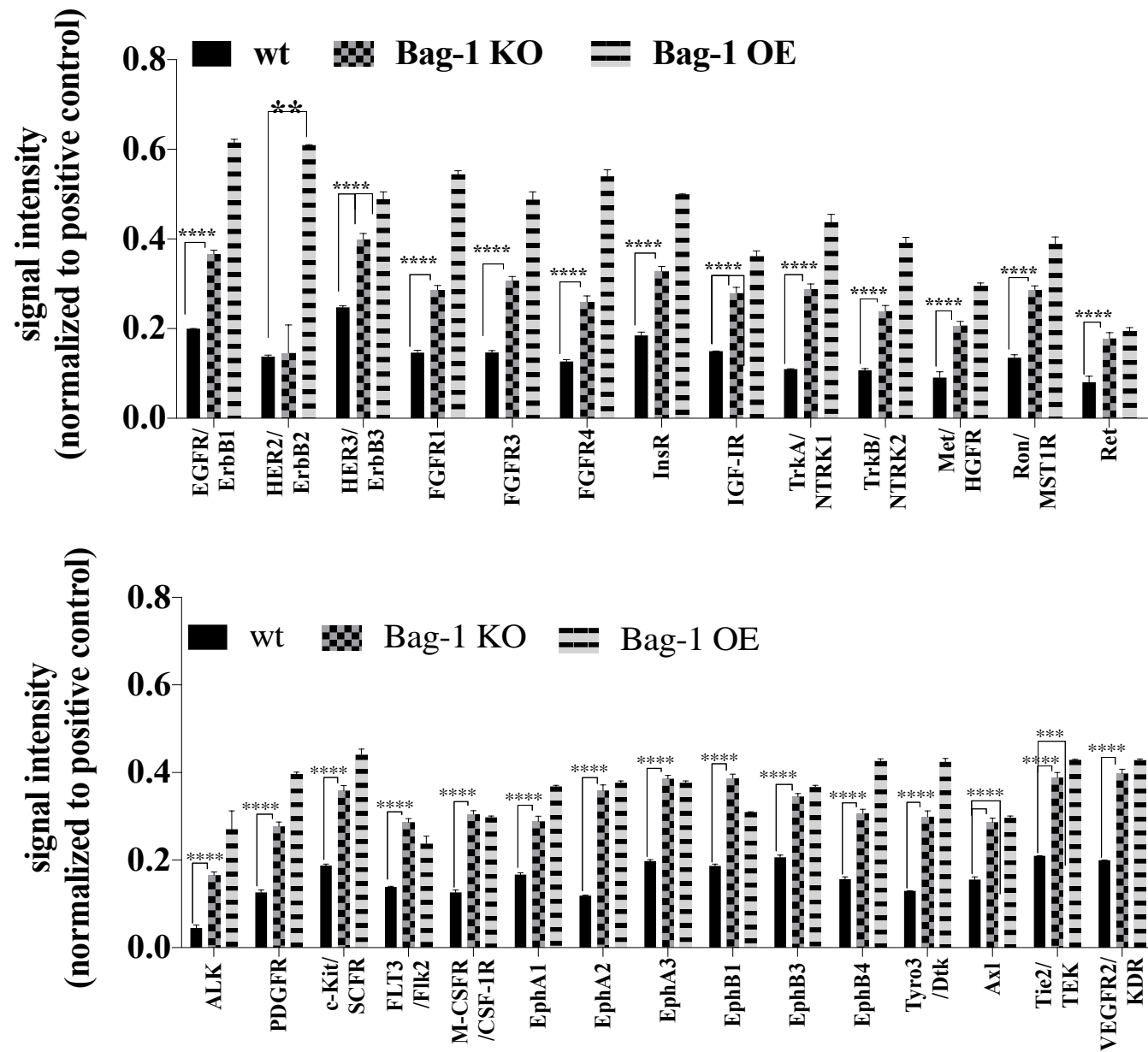

Fig S3 A (continued)

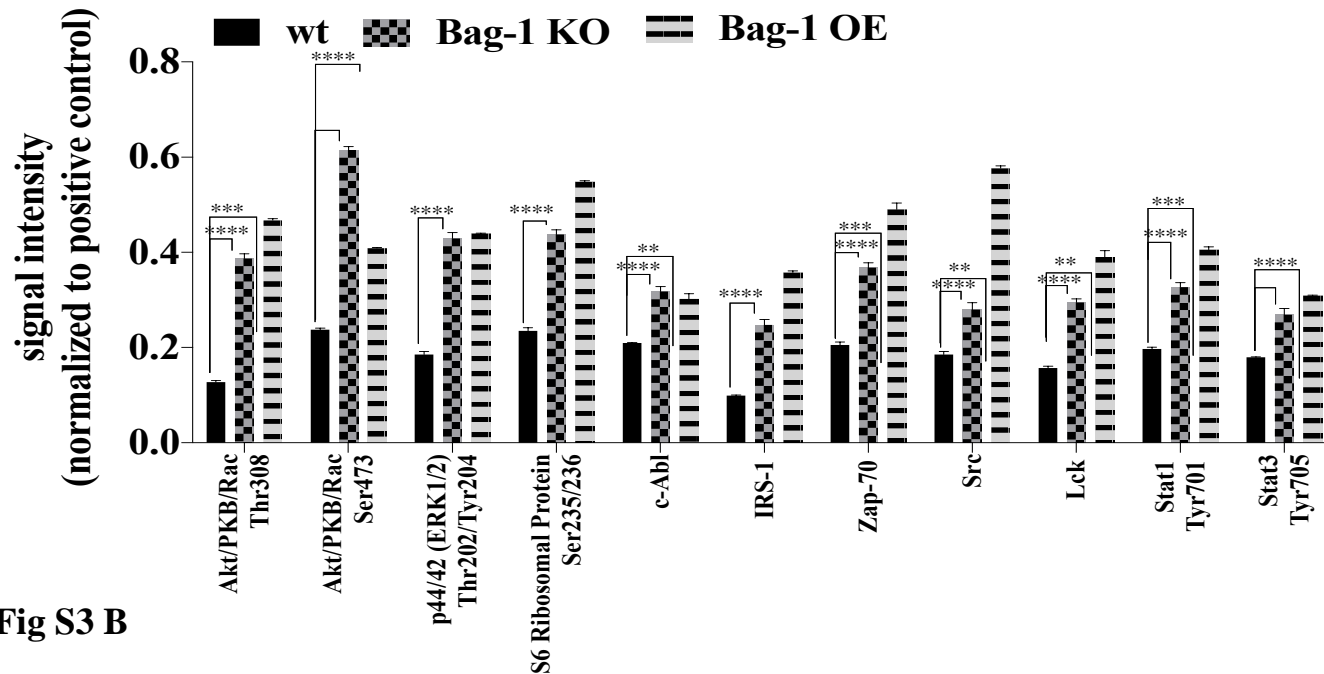

Fig S3 B

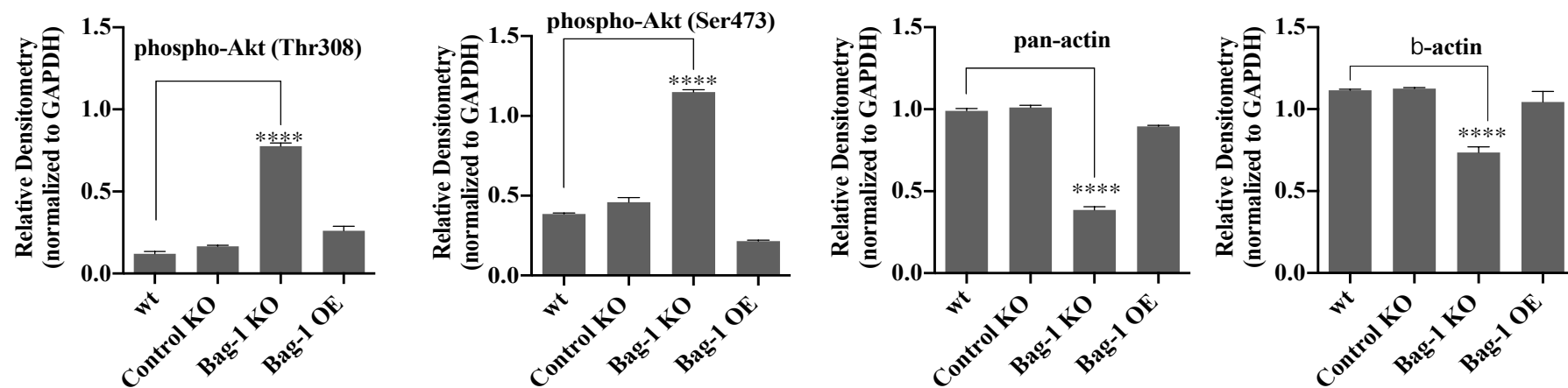

Supplement: S3 Fig — A) The expression profiles of 39 different RTKs that were obtained by PathScan RTK assay. The signal intensity of these proteins was normalized to positive control and analyzed GraphPad Prism version 8, https://www.graphpad.com/. B) Densitımetry analysis of immunoblotting results of Fig 3C. The relative densitometry analysis represented the mean ± SD of three independent experiments. (*** p = 0.0003, **** p< 00001 by Two-way ANOVA, Tukey’s multiple comparison test). (PDF) [file pone.0261062.s005.pdf]

**Fig S4 A**

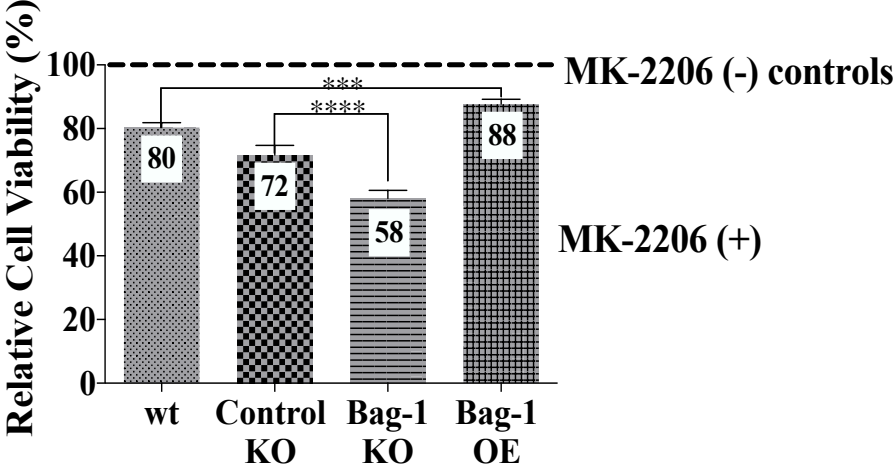

**Fig S4 B**

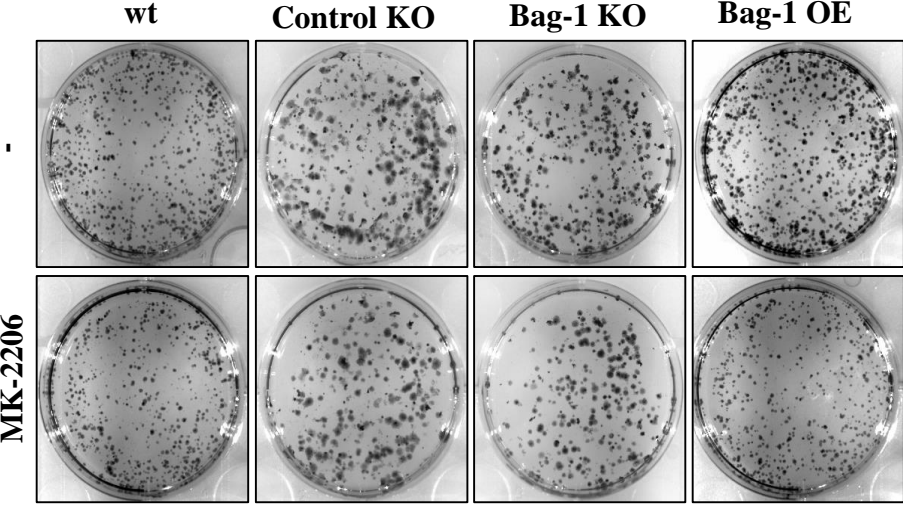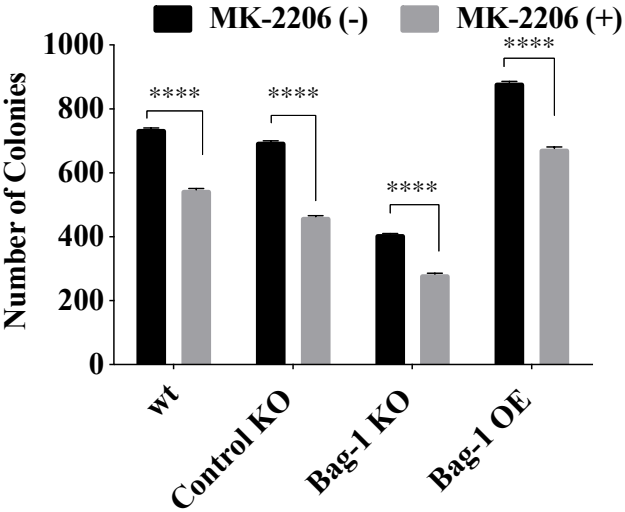

**Fig S4 C**

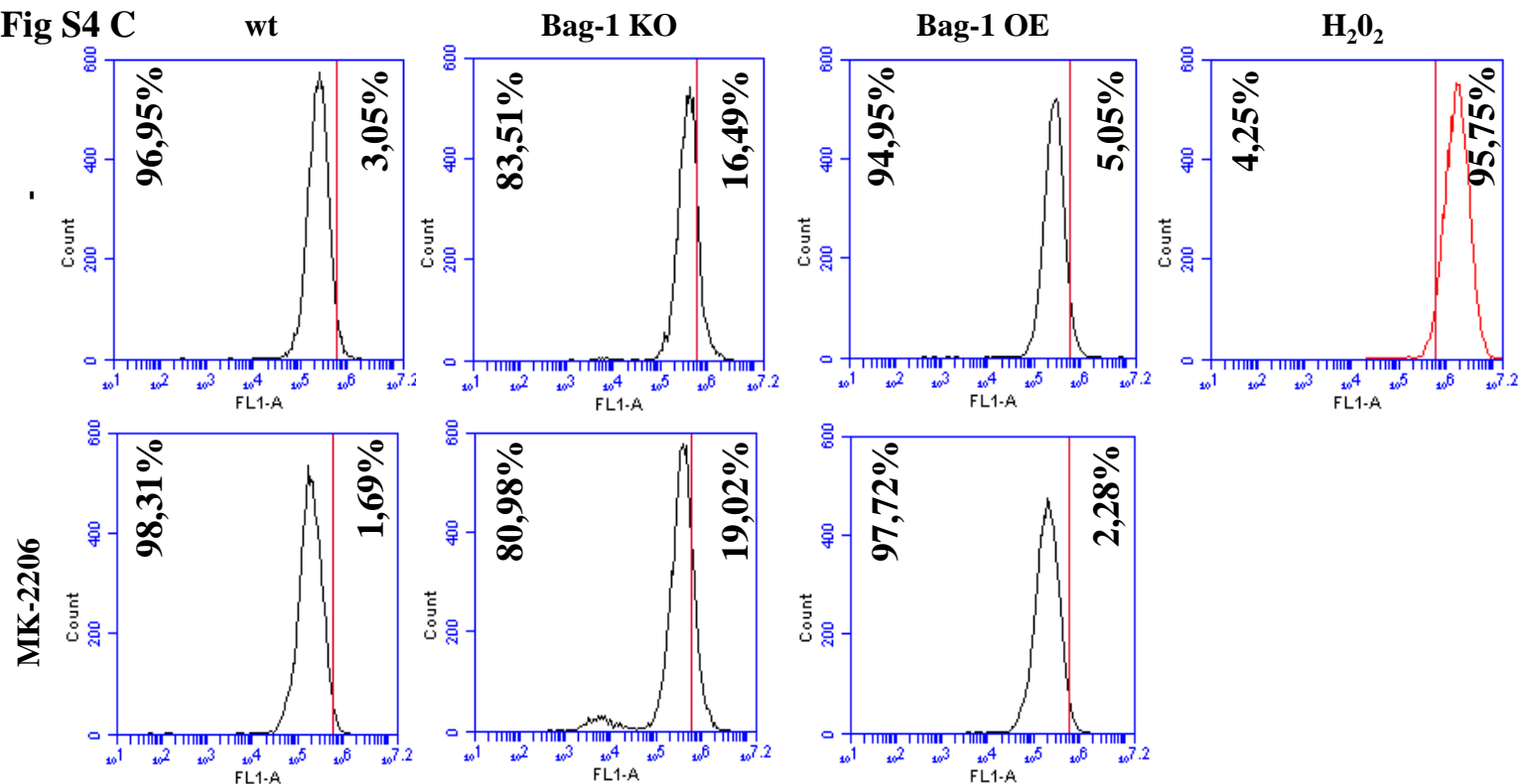

■ MK-2206 (+)  
■ MK-2206 (-)

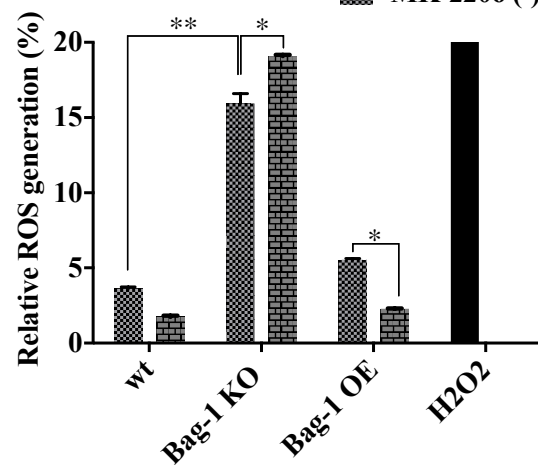

**Fig S4 D**

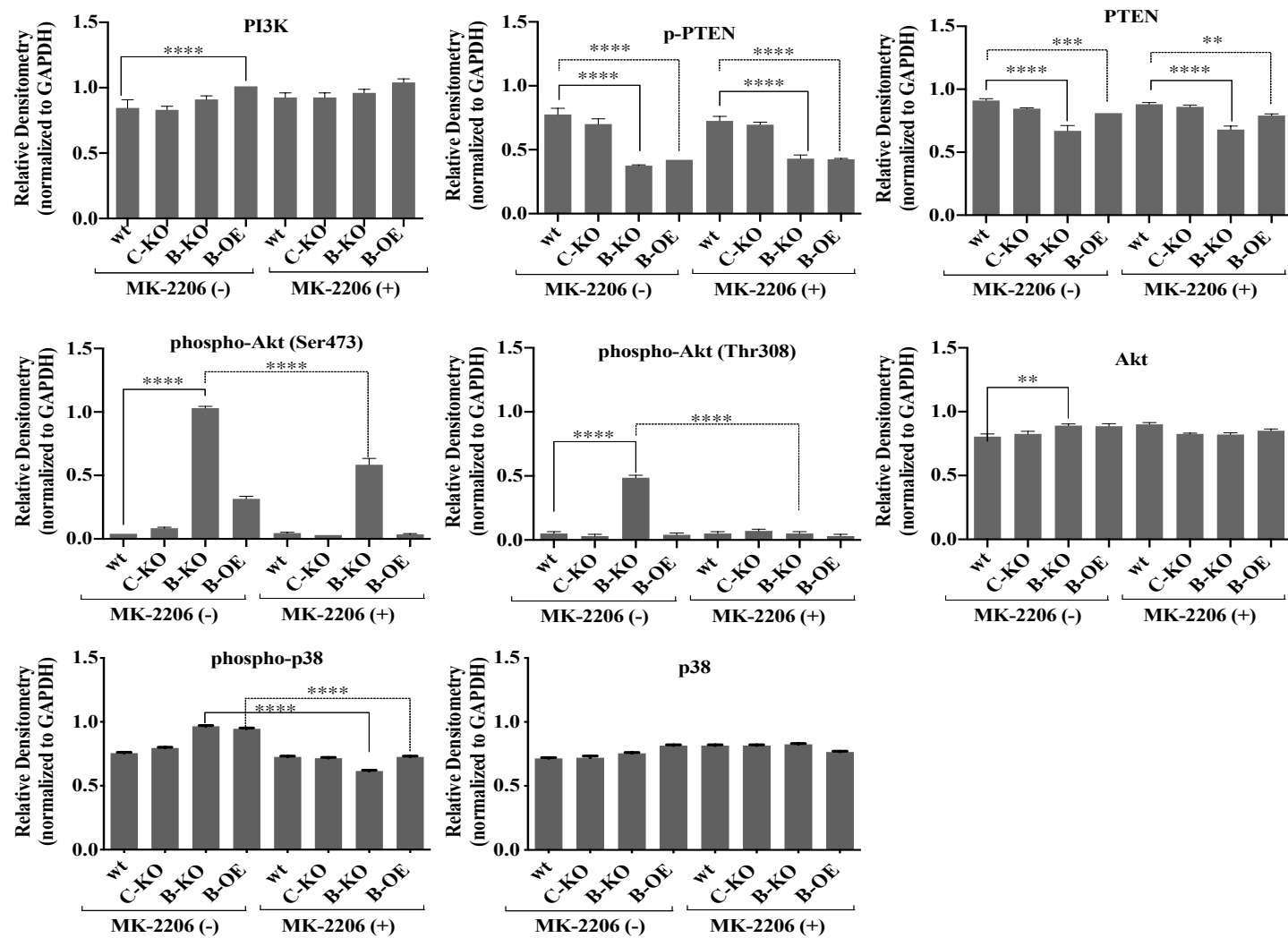

**Fig  
S4  
E**

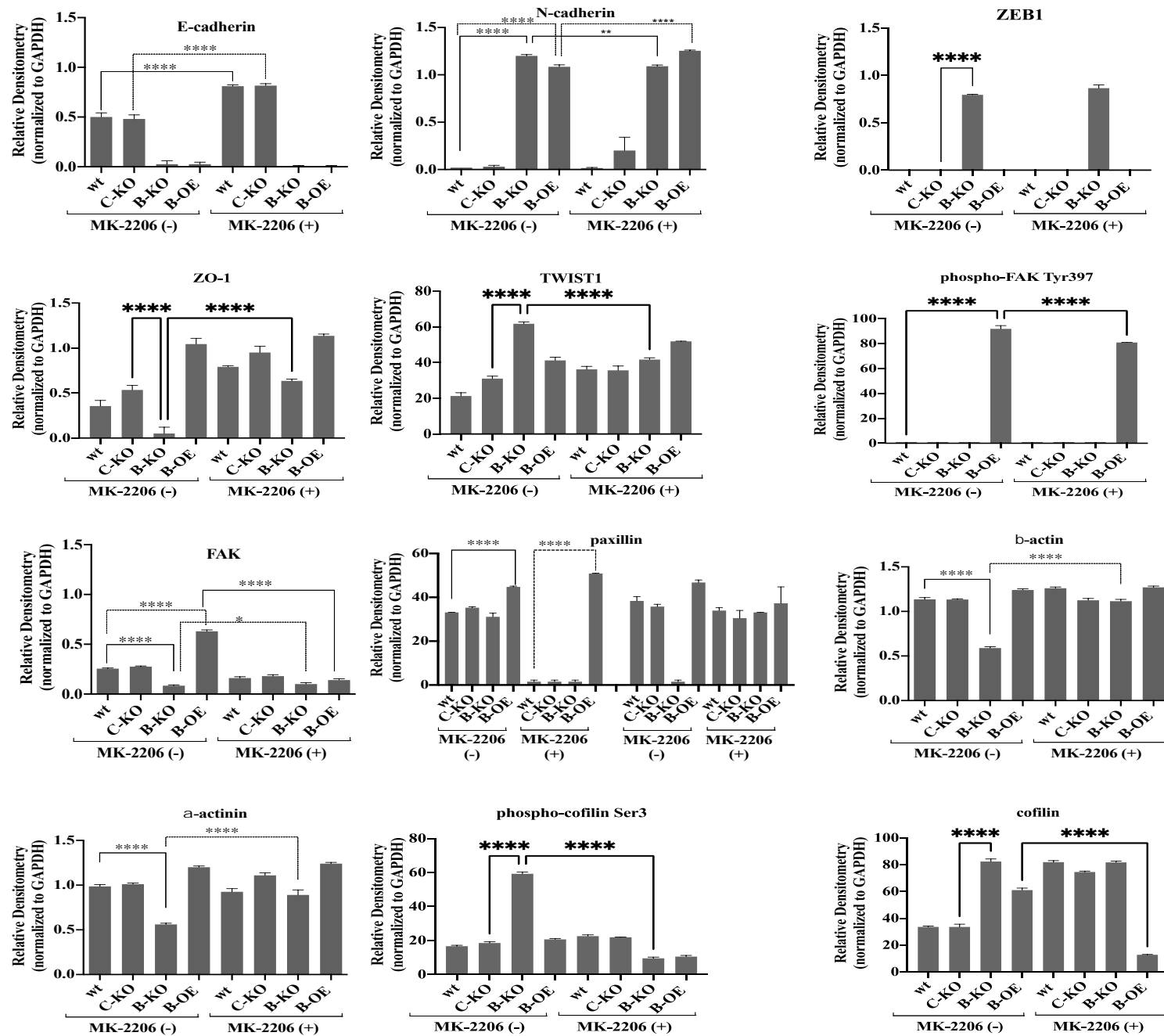

**Fig S4 F**

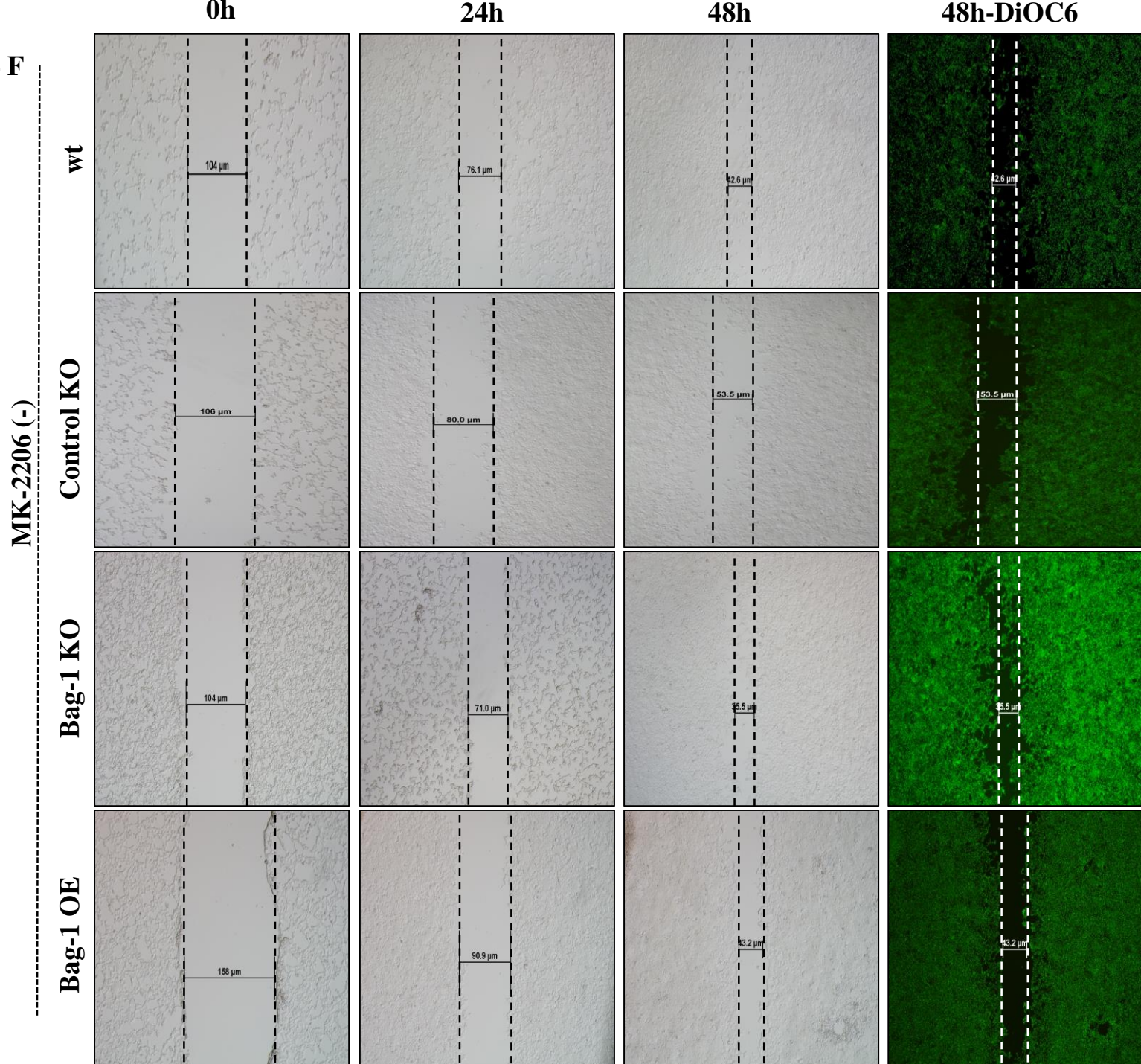

**Fig S4 G**

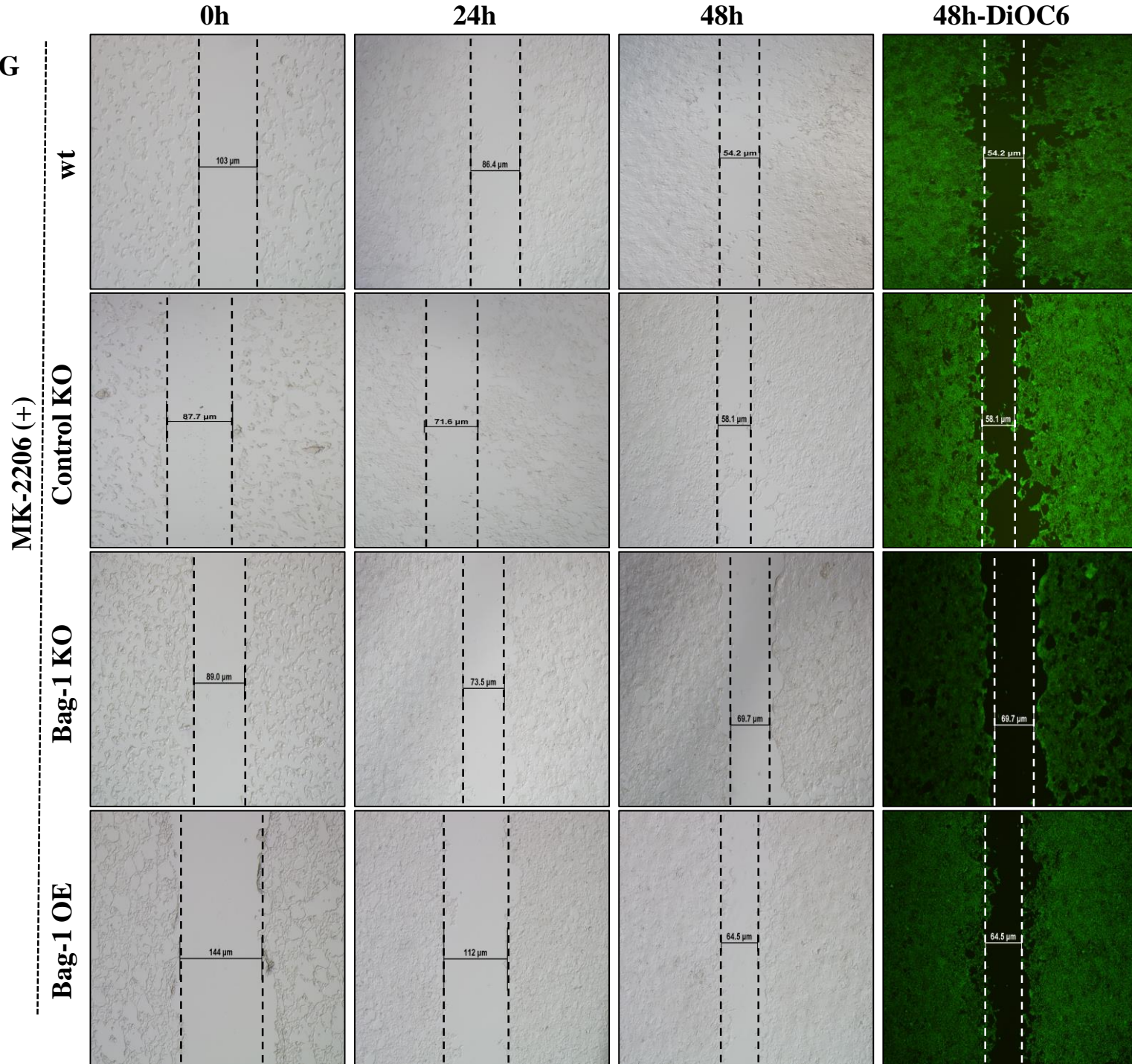

Fig S4 H

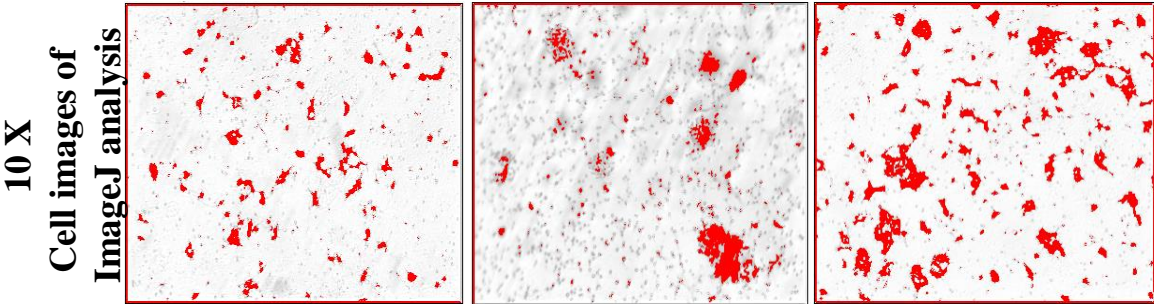

Fig S4 I

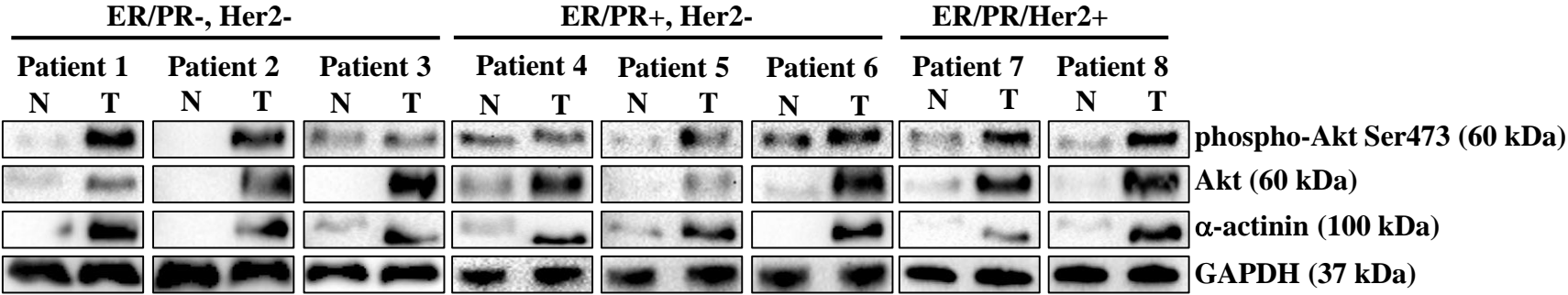

**Fig S4 J**

**ER/PR-, Her2-**

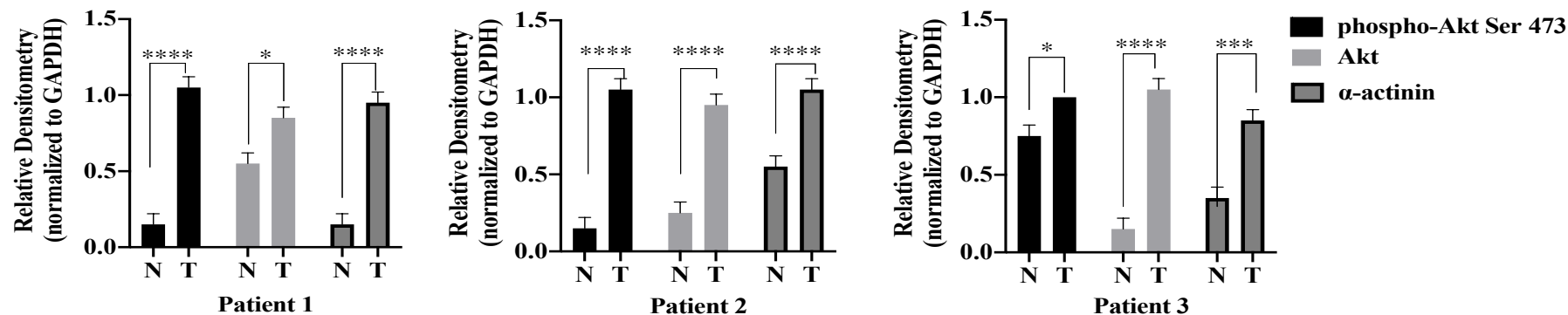

**ER/PR+, Her2-**

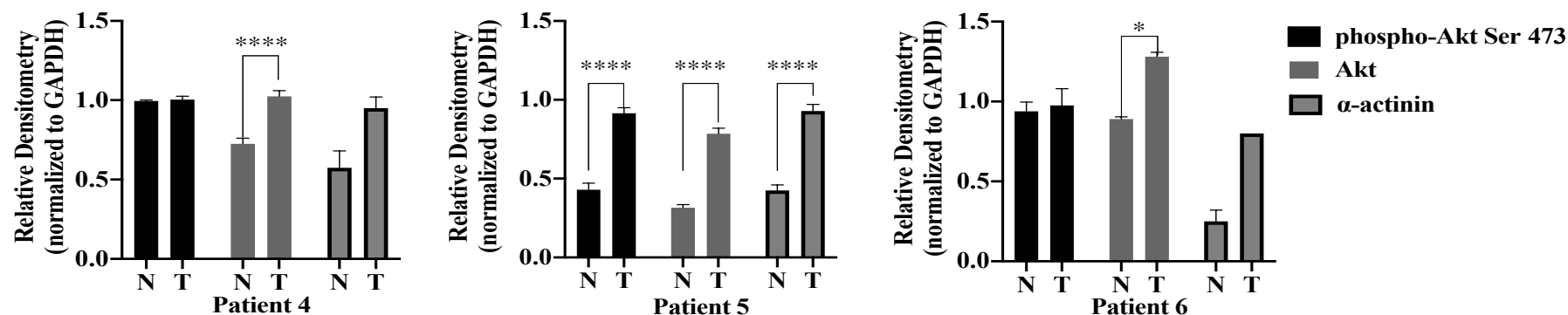

**ER/PR/Her2+**

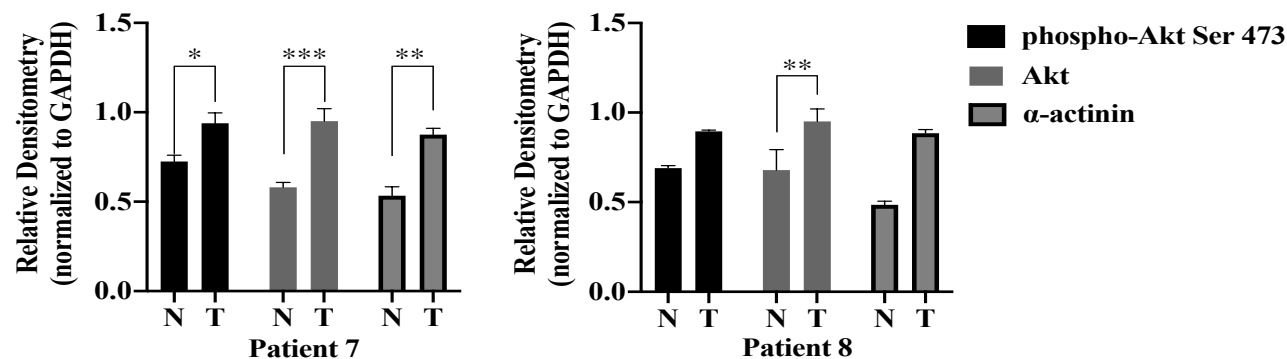

Supplement: S4 Fig — A) The effect of 24 h treatment of 500 nM MK-2206 on cells was determined by MTT Cell Viability assay. The bar histograms represent the mean ± SD of three independent experiments with at least four replicates. (*** p = 0.0003, **** p< 00001 by Two-way ANOVA, Sidak’s multiple comparison test). B) The effect of MK-2206 on colony formation potentials of wt, Control KO, Bag-1 KO, and Bag-1 OE cells was determined by colony formation assay. The number of colonies represented the mean ± SD of three repetitive experiments, measured by Image J based on their densities and were analyzed by Two-way ANOVA, Tukey’s multiple comparison test. (**** p< 0.0001). C) Cellular ROS generation of wt, Control KO, Bag-1 KO, and Bag-1 OE cells was measured by DCFH-DA staining and analyzed by BD Accuri C6 flow cytometer (* p< 0.05 and ** p< 0.01, **** p< 0.0001). D) Densitometry analysis of Fig 4A. E) Densitometry analysis of Fig 4B. The relative densitometry analysis represented the mean ± SD of three independent experiments. GAPDH was used as a loading control. (*p = 0.0114, **p = 0.0013, ***p = 0.0006, ****p<0.0001 by Two-way ANOVA, Tukey’s multiple comparison test). F-G) Wound healing images of wt, Control KO, Bag-1 KO and Bag-1 OE cells with MK-2206 treatment in time dependent manner. H) Cell images of Boyden Chamber assay that was analyzed by ImageJ. Red dots showed the cells that counted as migrated cells. I-J) Immunoblotting (I) and densitometry analysis (J) of the expression profiles of phospho-Akt Ser473, total Akt and α-actinin in different breast tissue subtypes. (PDF) [file pone.0261062.s006.pdf]

**Fig S5 A**

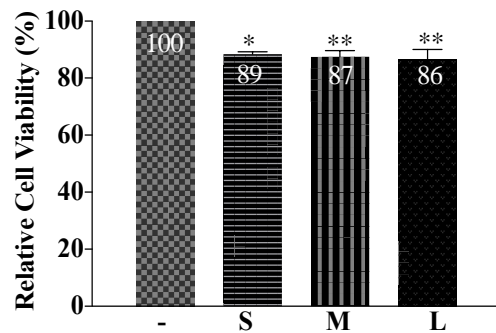

**Fig S5 B**

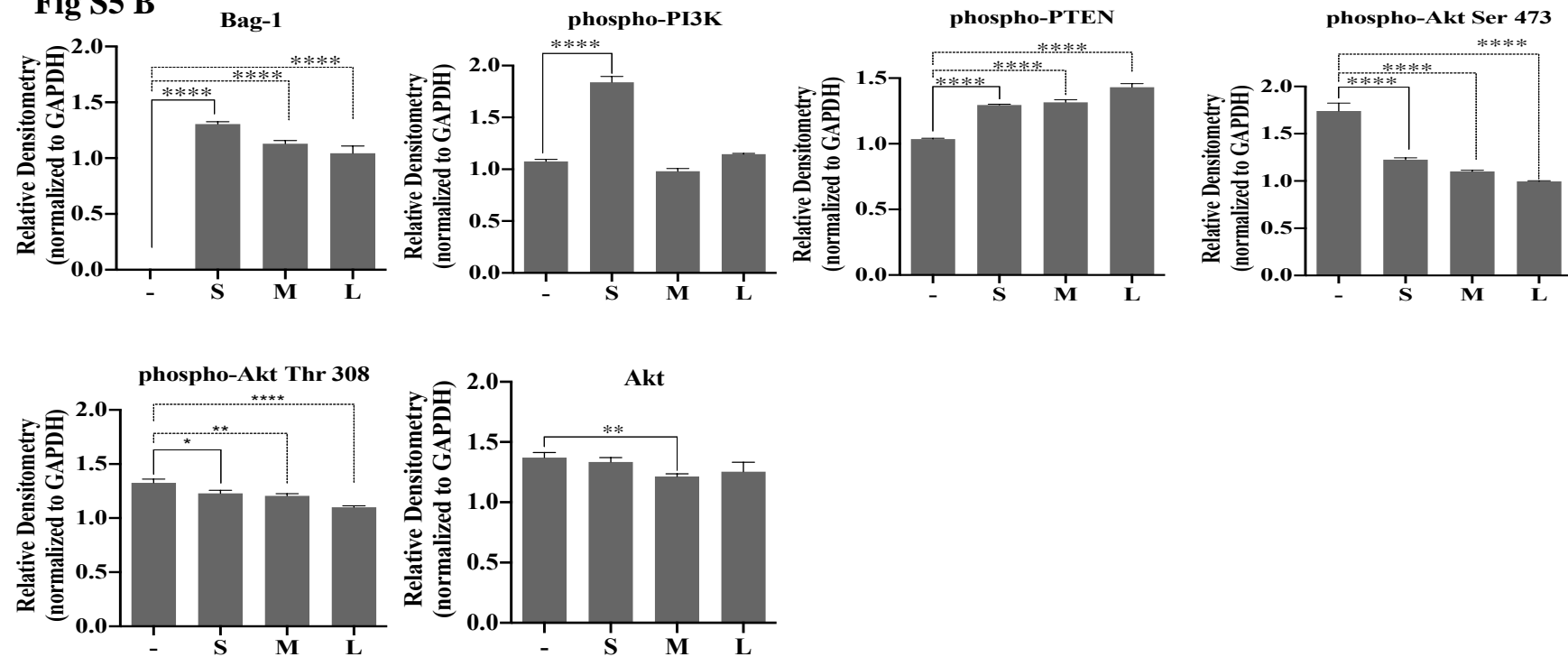

**Fig S5 C**

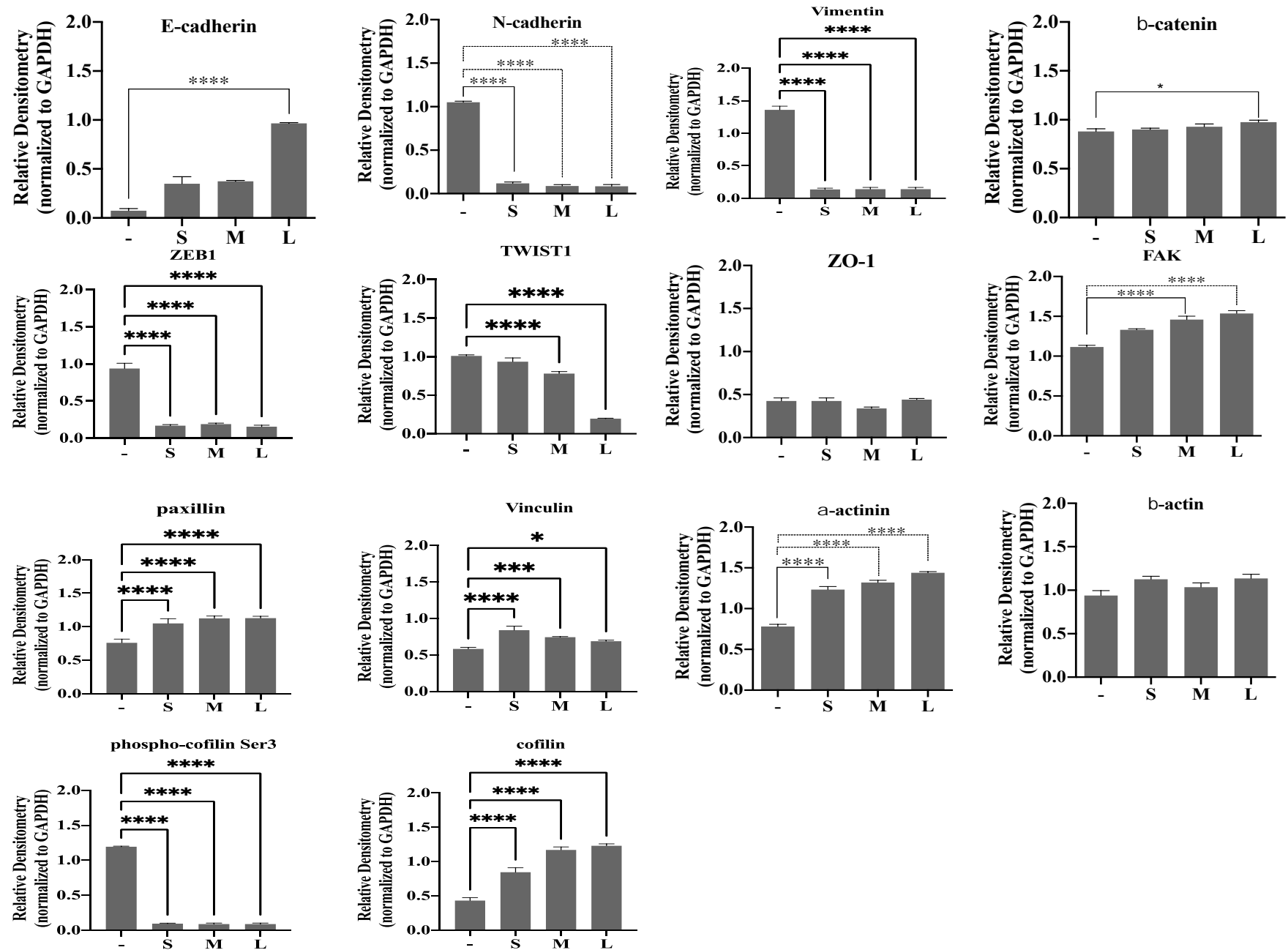

**Fig S5 D**

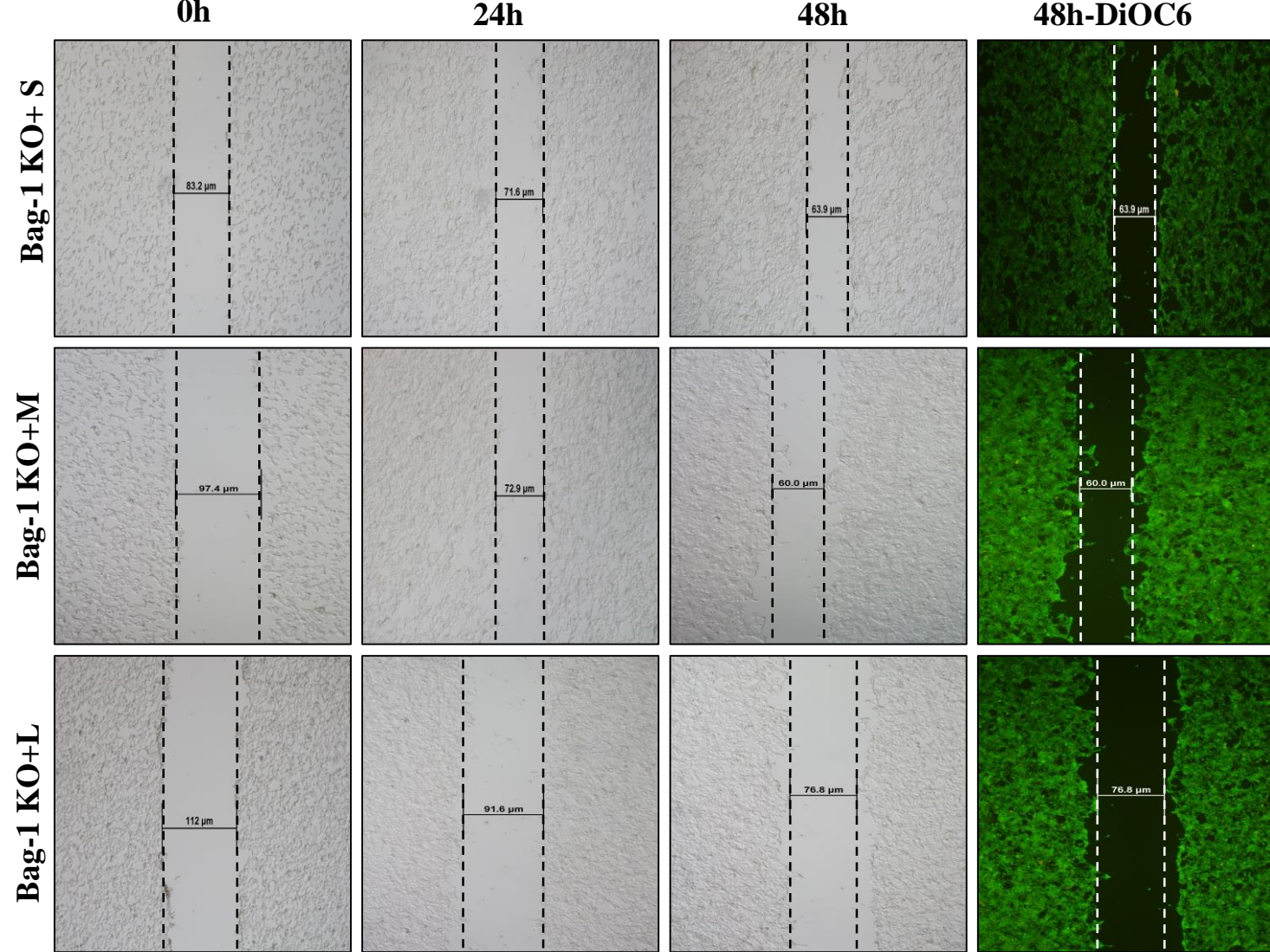

**Fig S5 E**

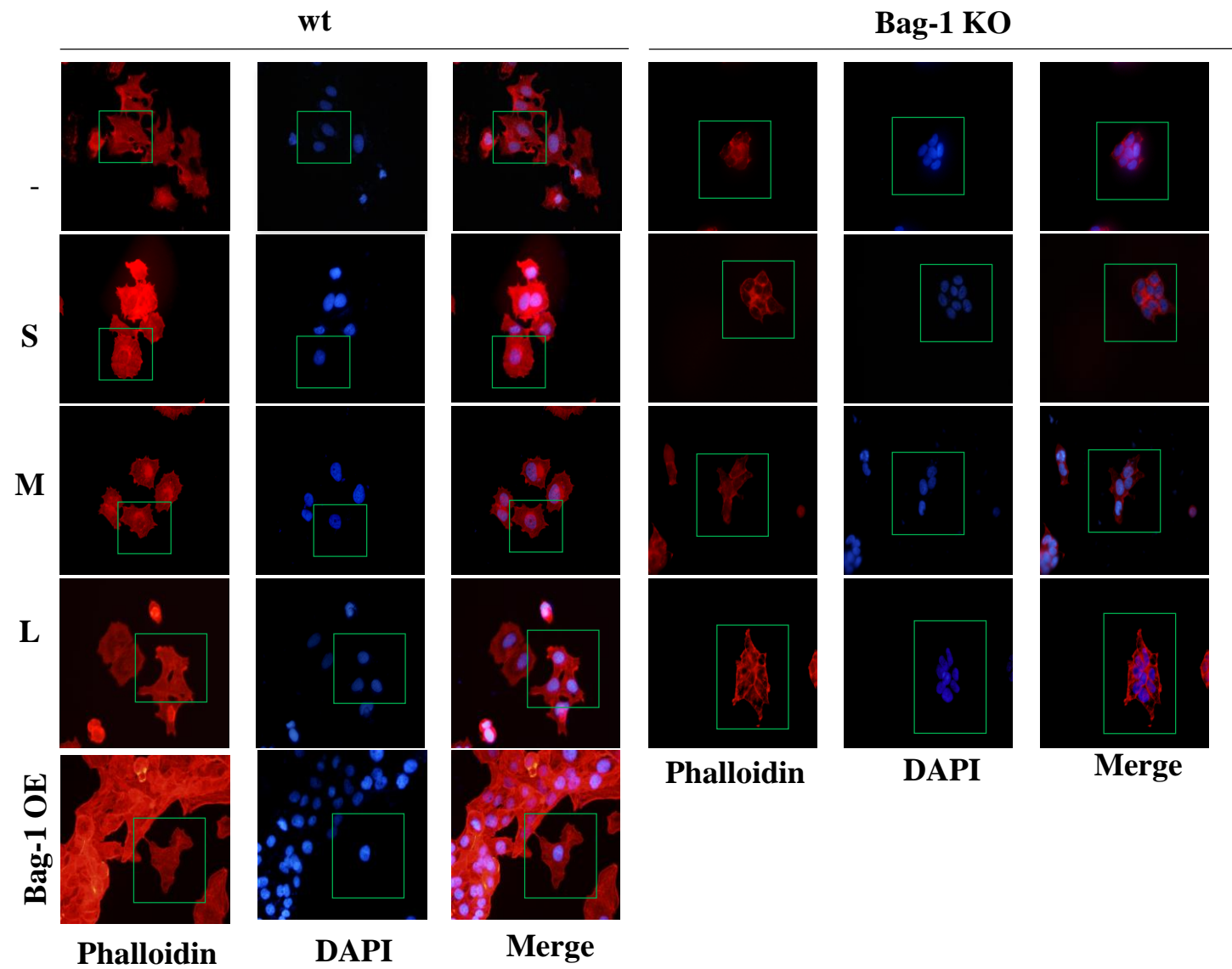

Supplement: S5 Fig — A) The cell viability of Bag-1 KO cells and transfected with Bag-1S, M and L isoforms, respectively was determined by MTT Cell Viability assay. B-C) Densitometry analyses of immunoblotting results of Bag-1 KO cells and transfected with Bag-1S, M and L isoforms. **** p< 00001 by Two-way ANOVA, Tukey’s multiple comparison test). D) Time-dependent wound healing images of Bag-1 KO cells and transfected with Bag-1S, M and L isoforms. E) Visualization of the actin cytoskeleton in Bag-1 isoform plasmid transfected MCF-7 wt and Bag-1 KO cells through fluorescent-labeled Phalloidin incubation by immunofluorescence experiment described in “Material and methods”. (PDF) [file pone.0261062.s007.pdf]
