# Supplementary material for: CRISPR/Cas9-mediated Bag-1 knockout increased mesenchymal characteristics of MCF-7 cells via Akt hyperactivation-mediated actin cytoskeleton remodeling
Source: PLoS One. 2022 Jan 7;17(1):e0261062. doi: 10.1371/journal.pone.0261062 (PMC8741009; doi:10.1371/journal.pone.0261062)
Supplement: S1 Raw images — (PDF) [file pone.0261062.s008.pdf]

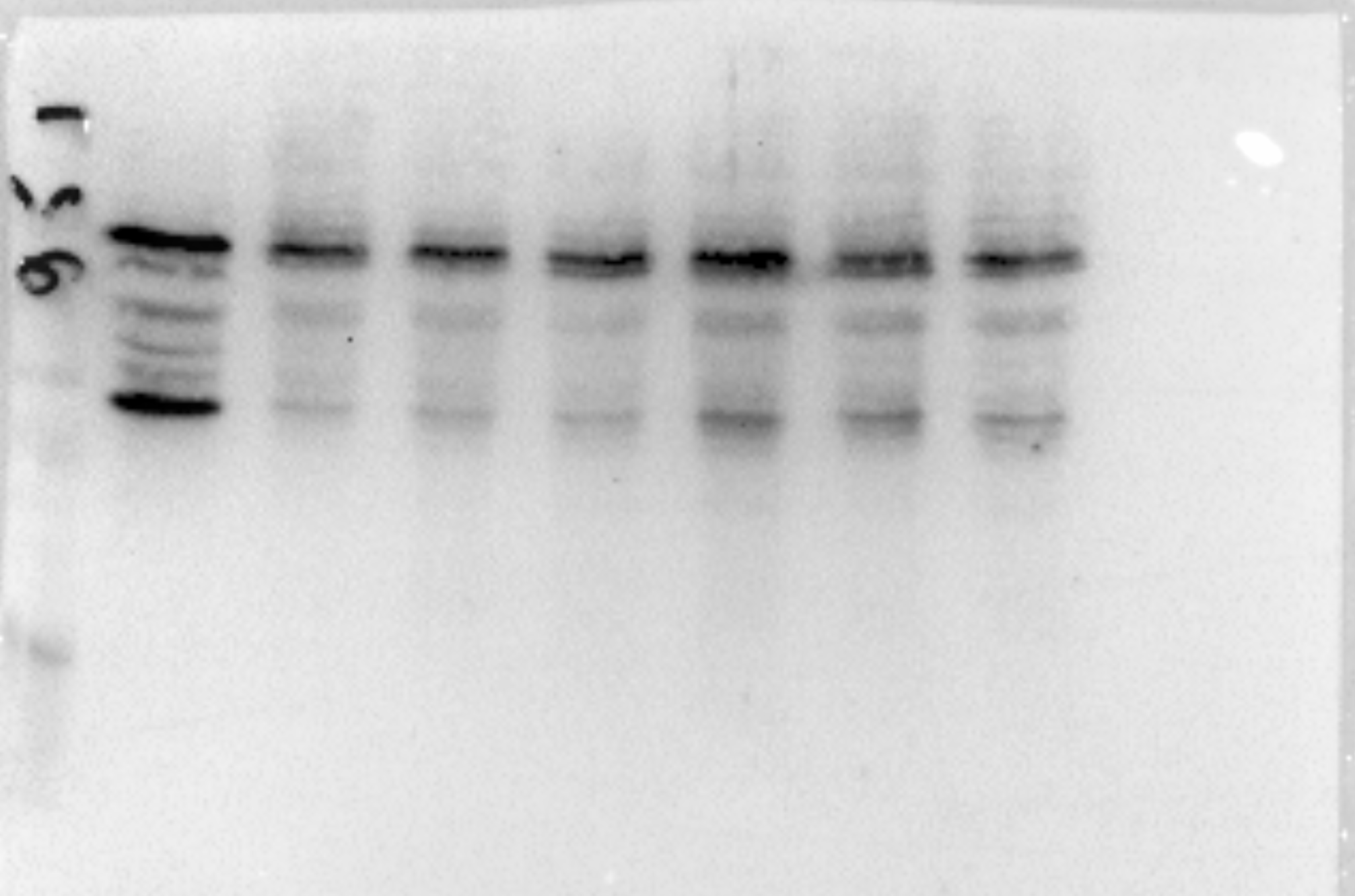

Fig1A- Bag-1 left panel

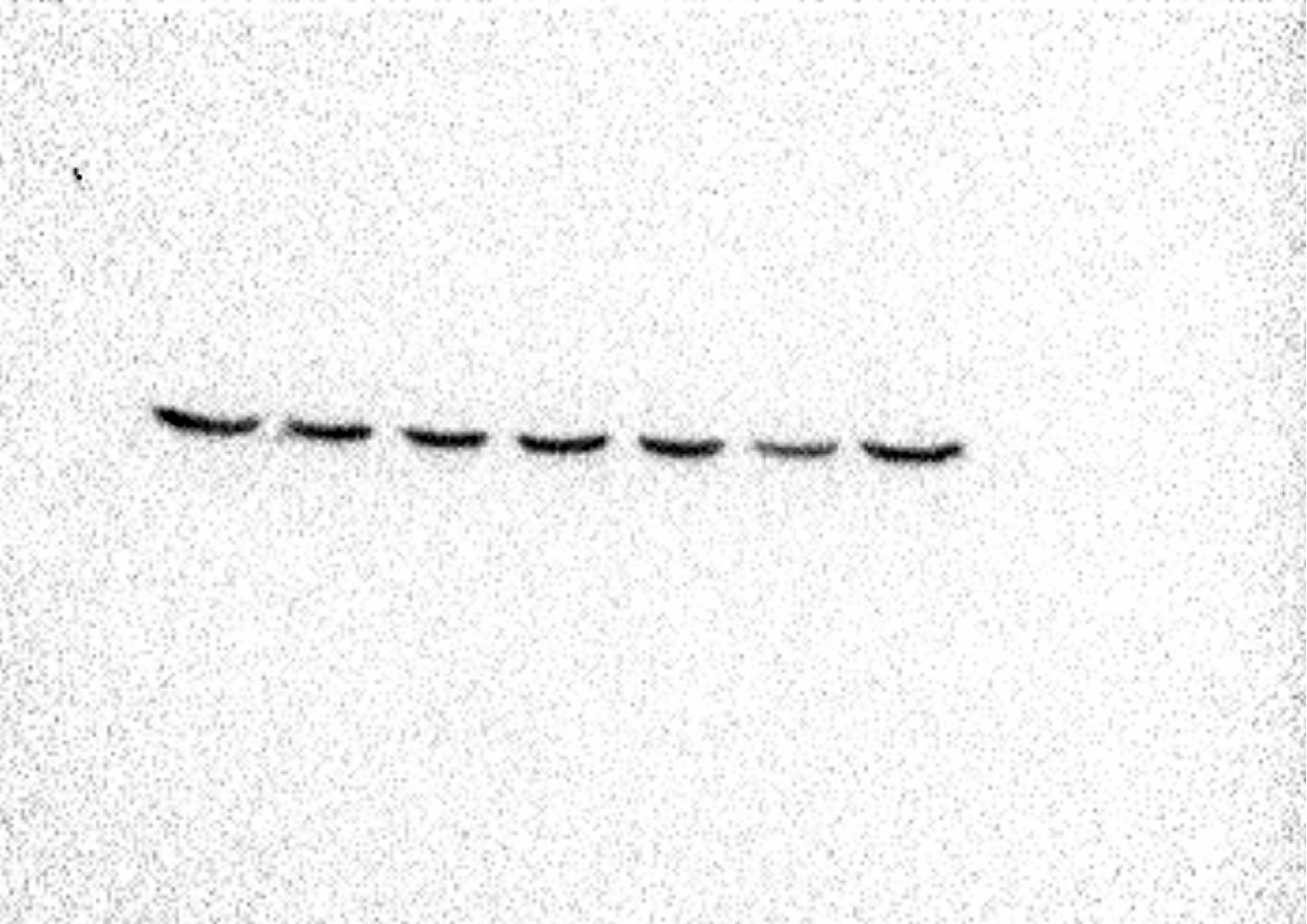

Fig1A-GAPDH left panel

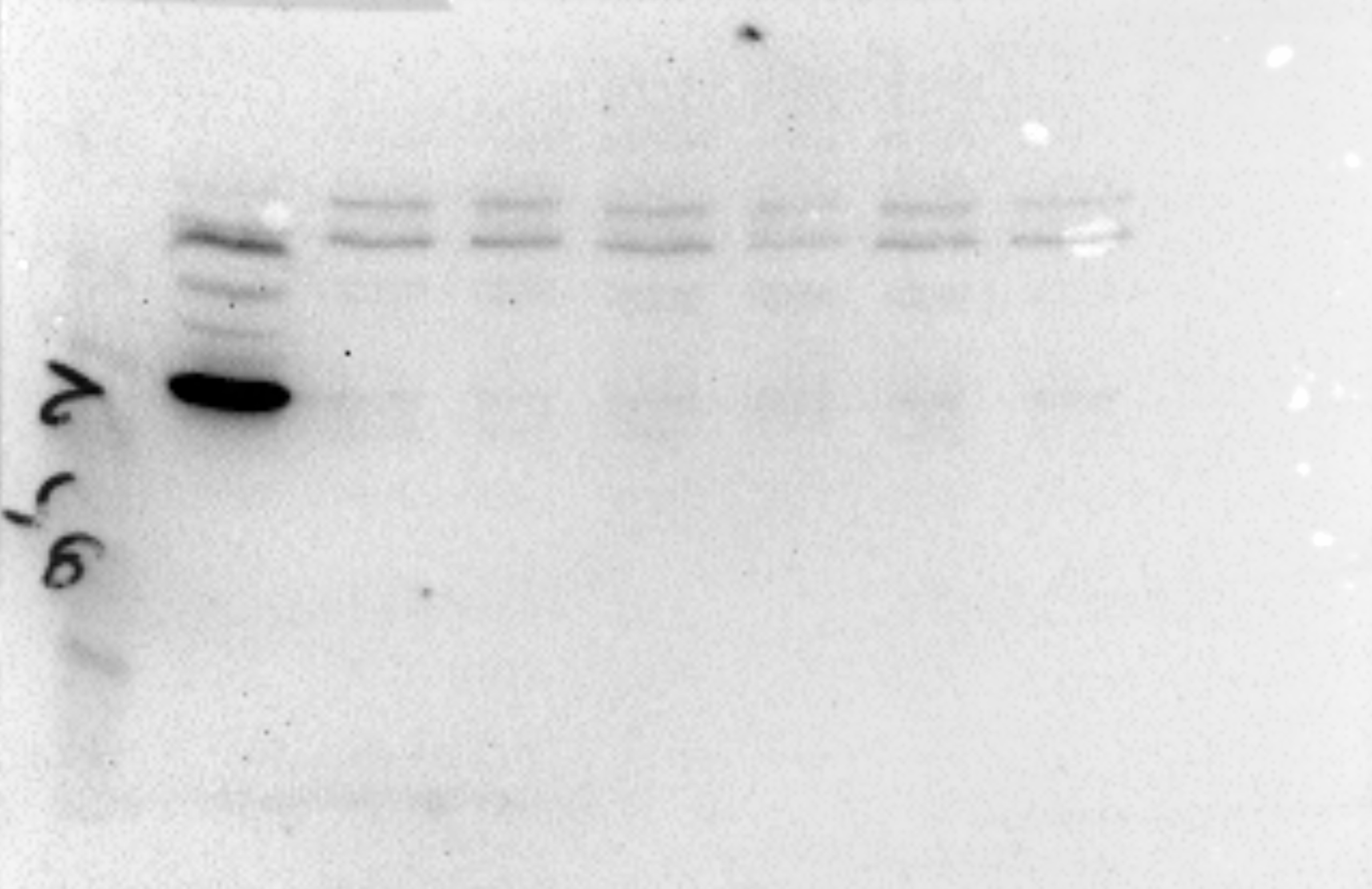

Fig1A-Bag-1-middle panel

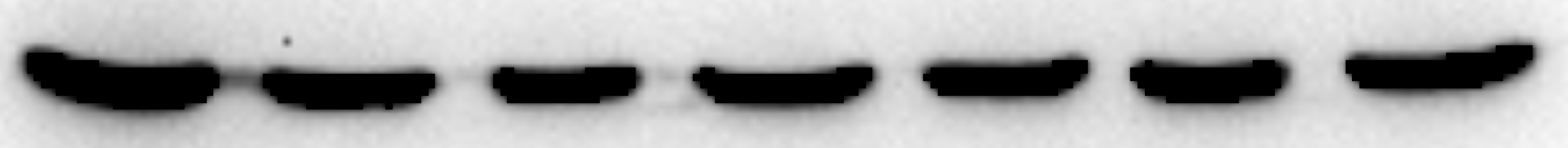

Fig1A-GAPDH-middle panel

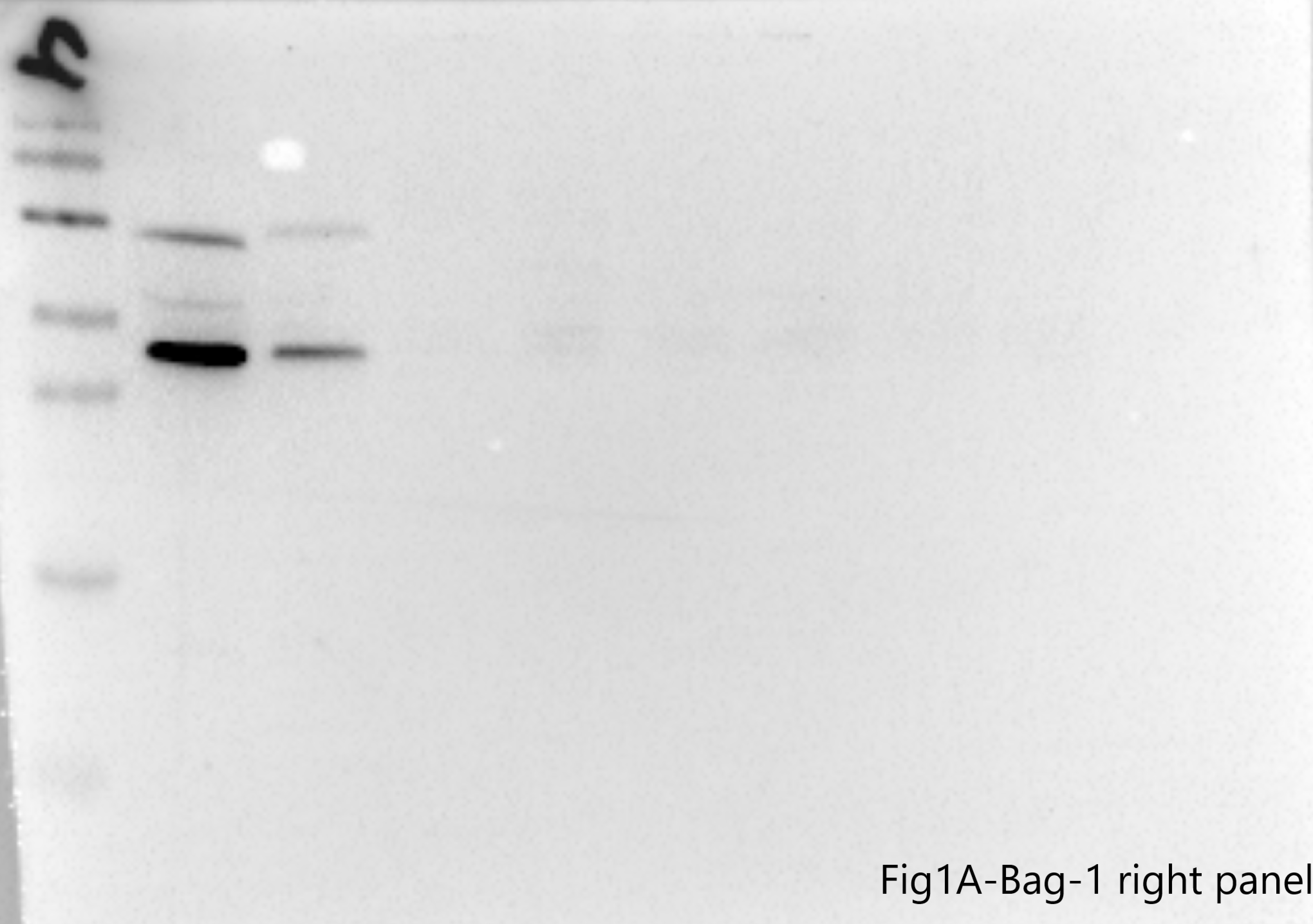

Fig1A-Bag-1 right panel

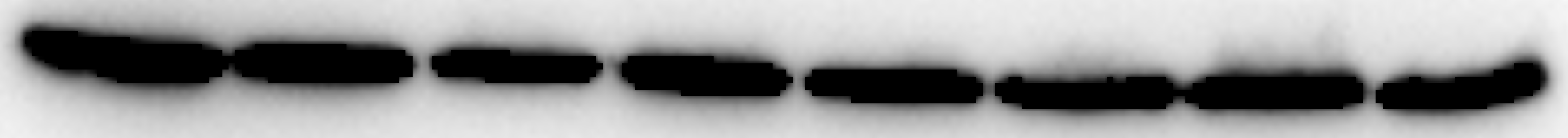

Fig1A-GAPDH-right panel

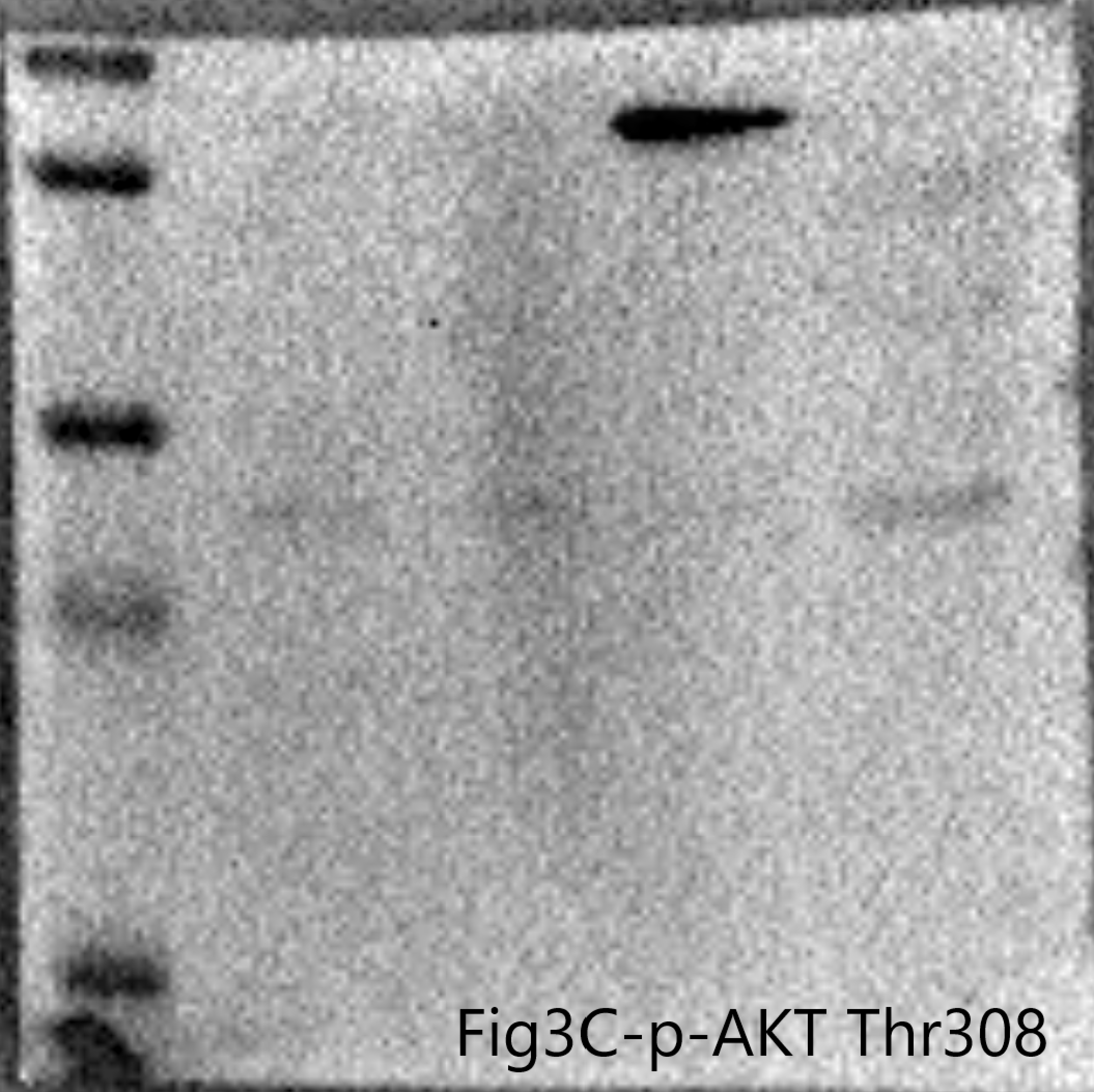
Fig3C-p-AKT Thr308

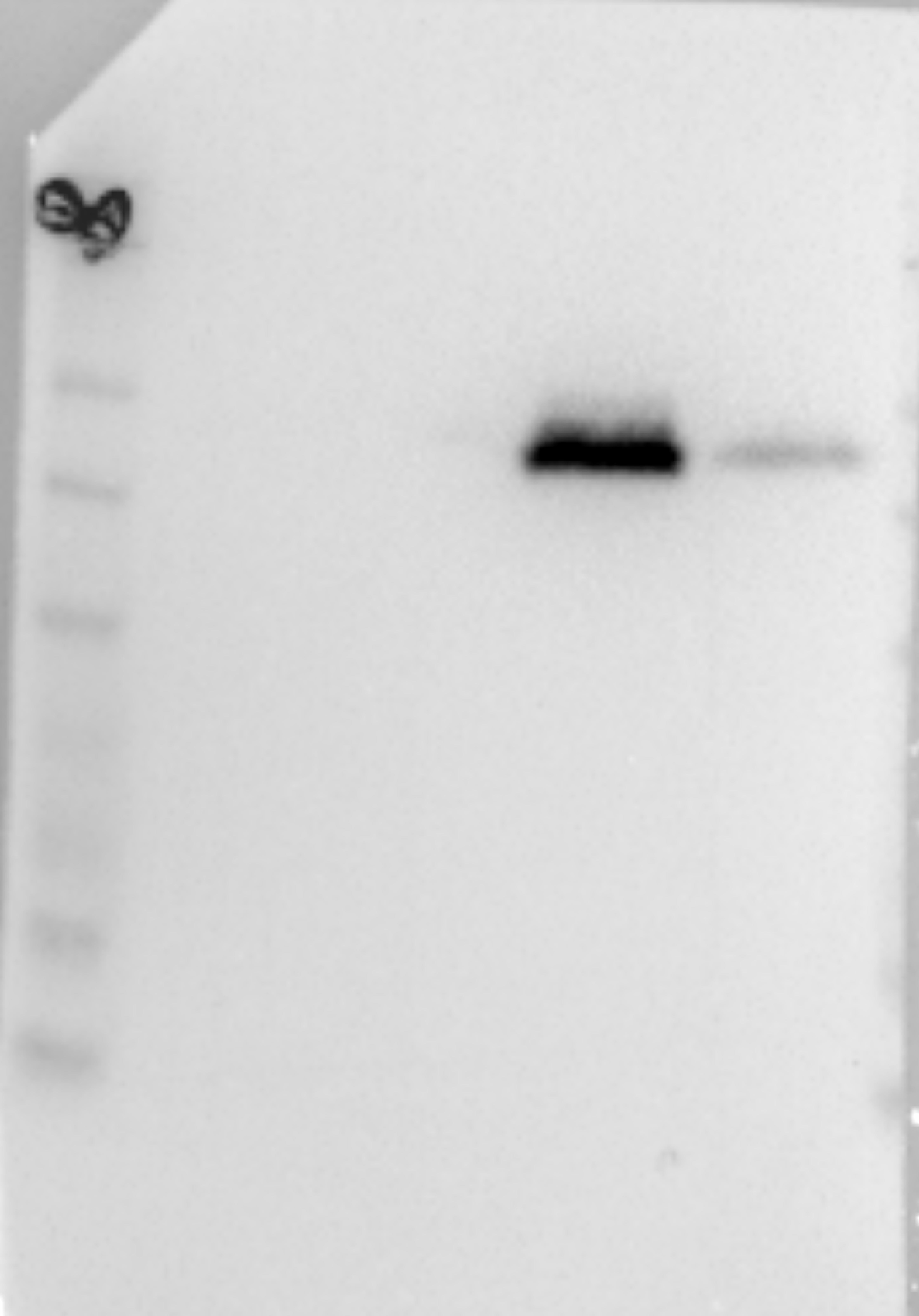

Fig3C-p-Akt Ser473

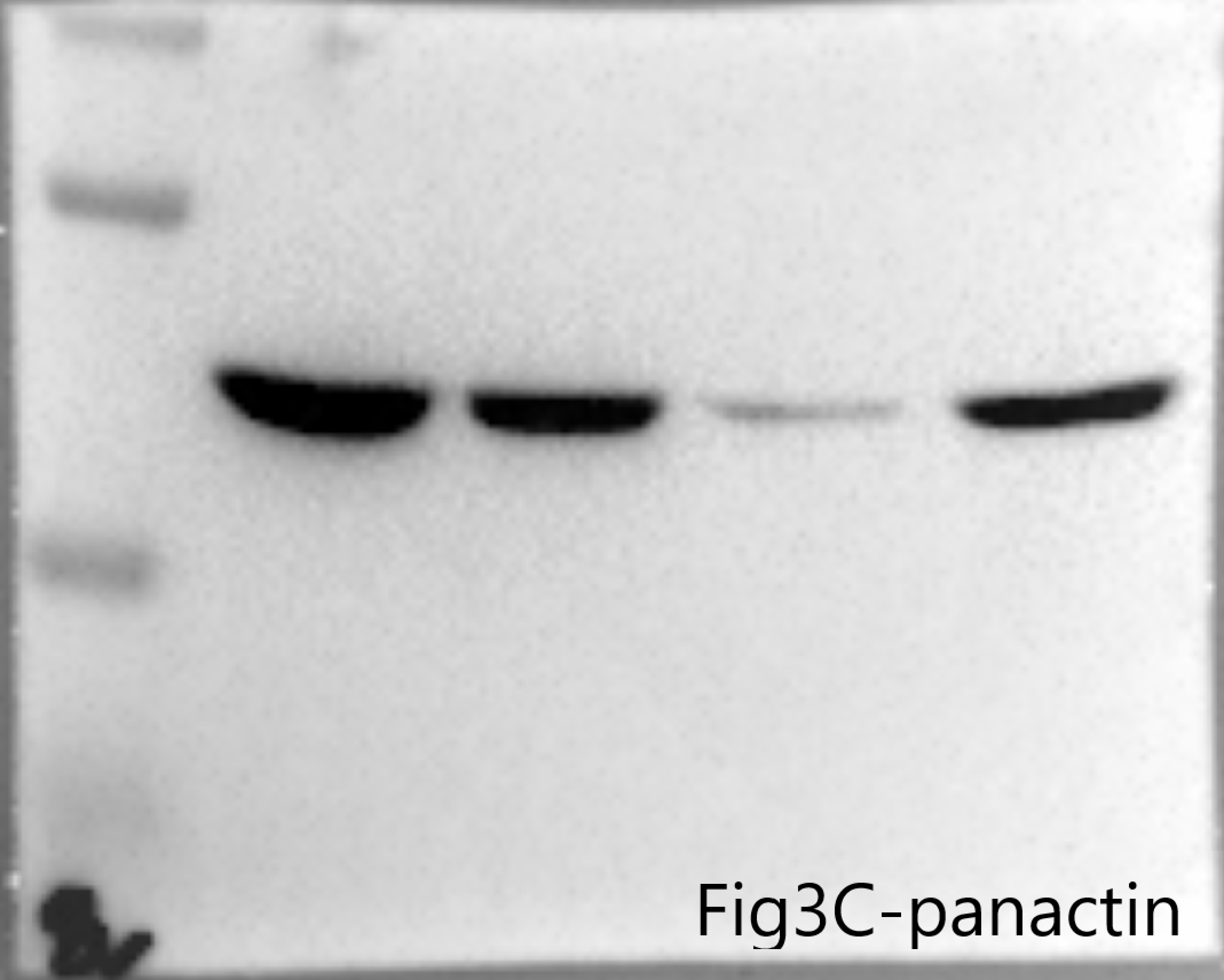
Fig3C-panactin

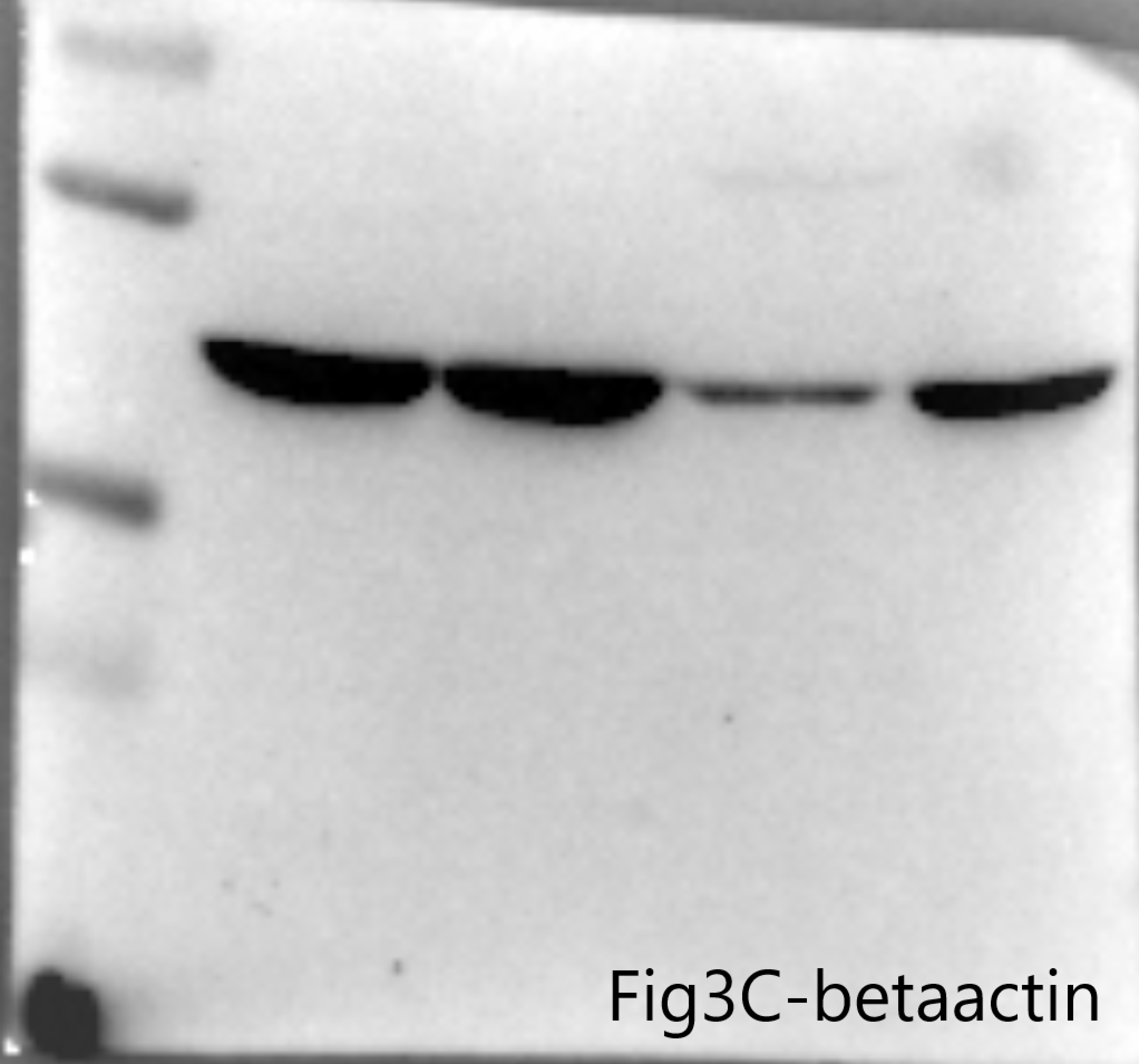
Fig3C-betaactin

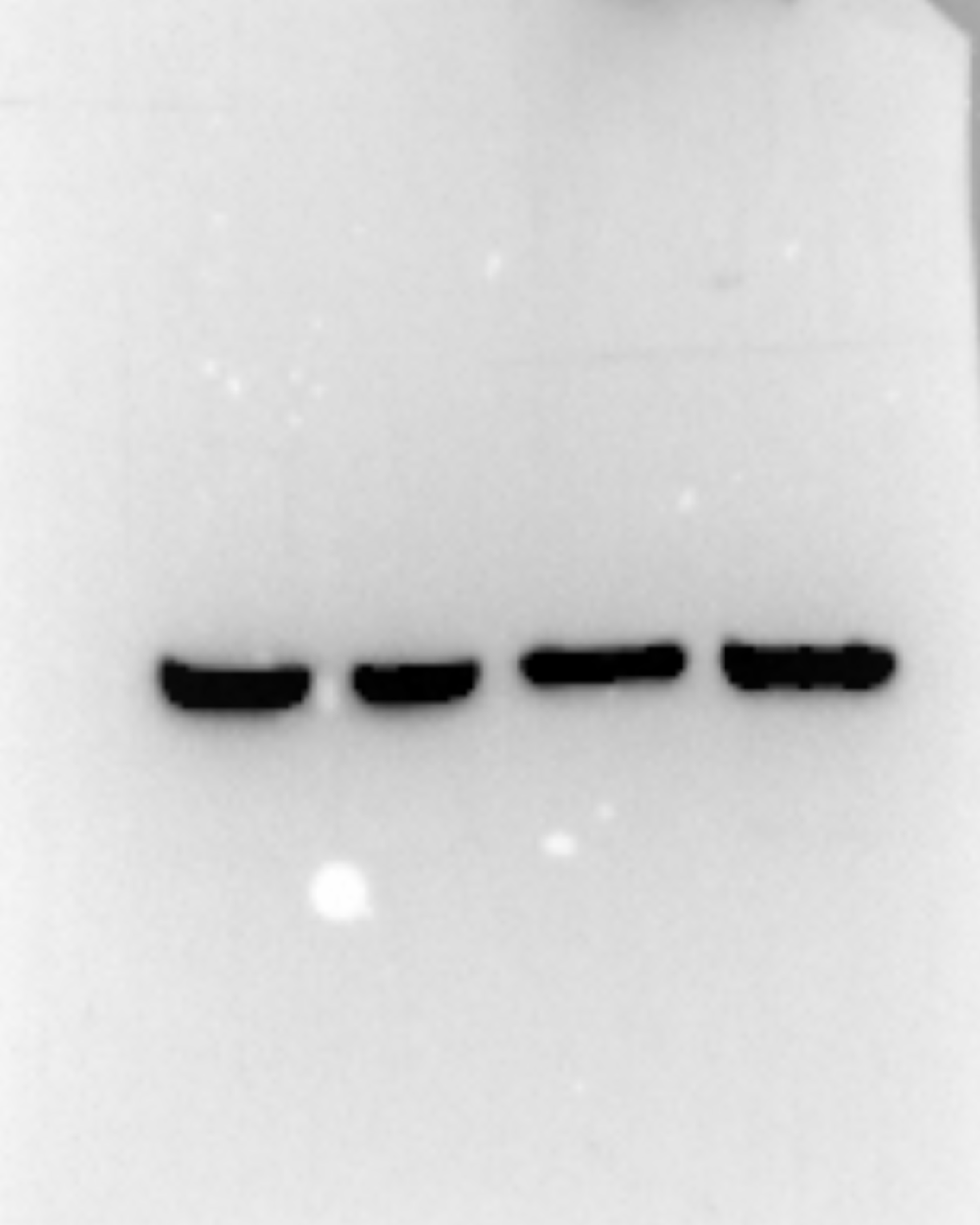

Fig3C-GAPDH

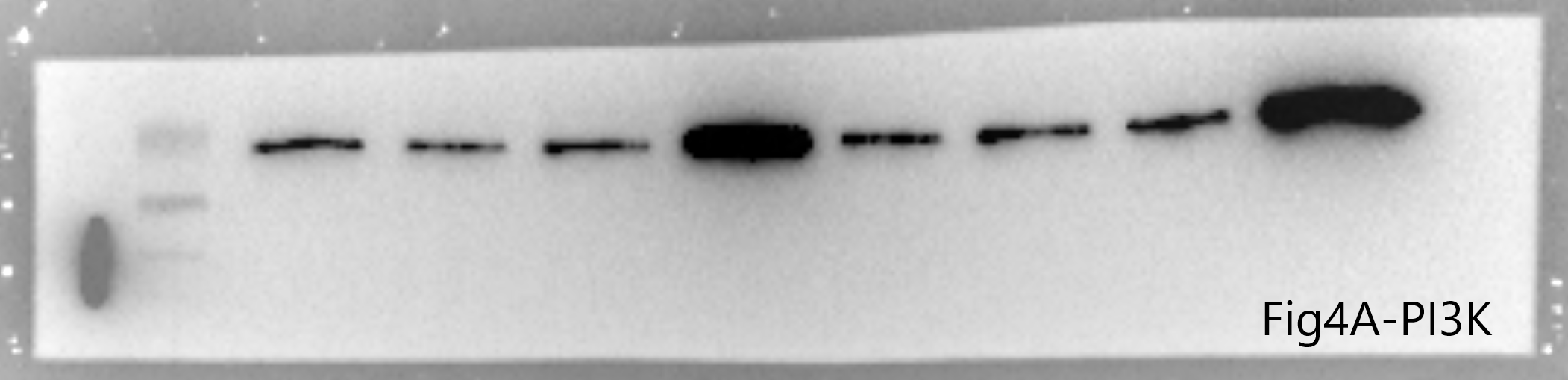
Fig4A-PI3K

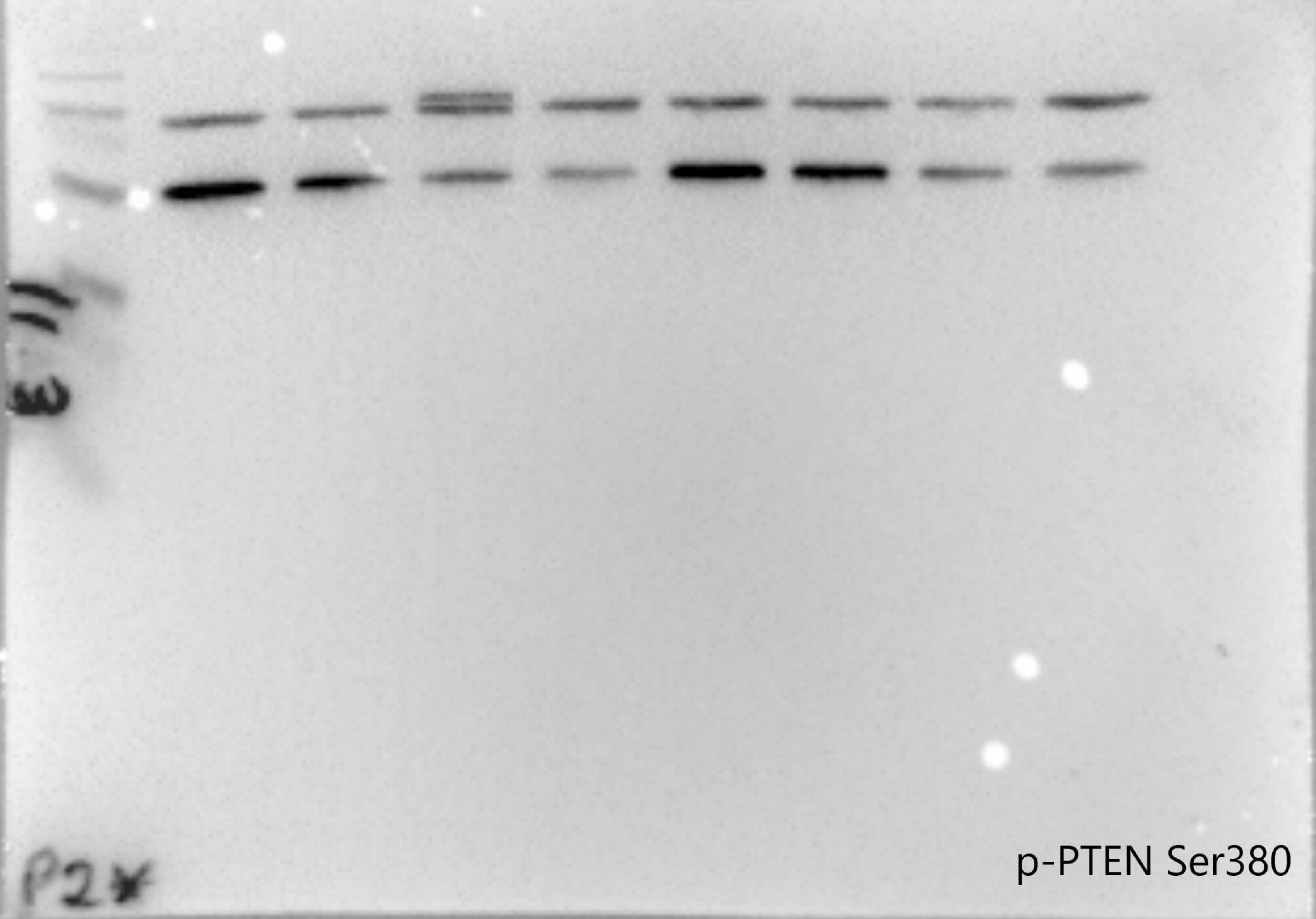
P2
p-PTEN Ser380

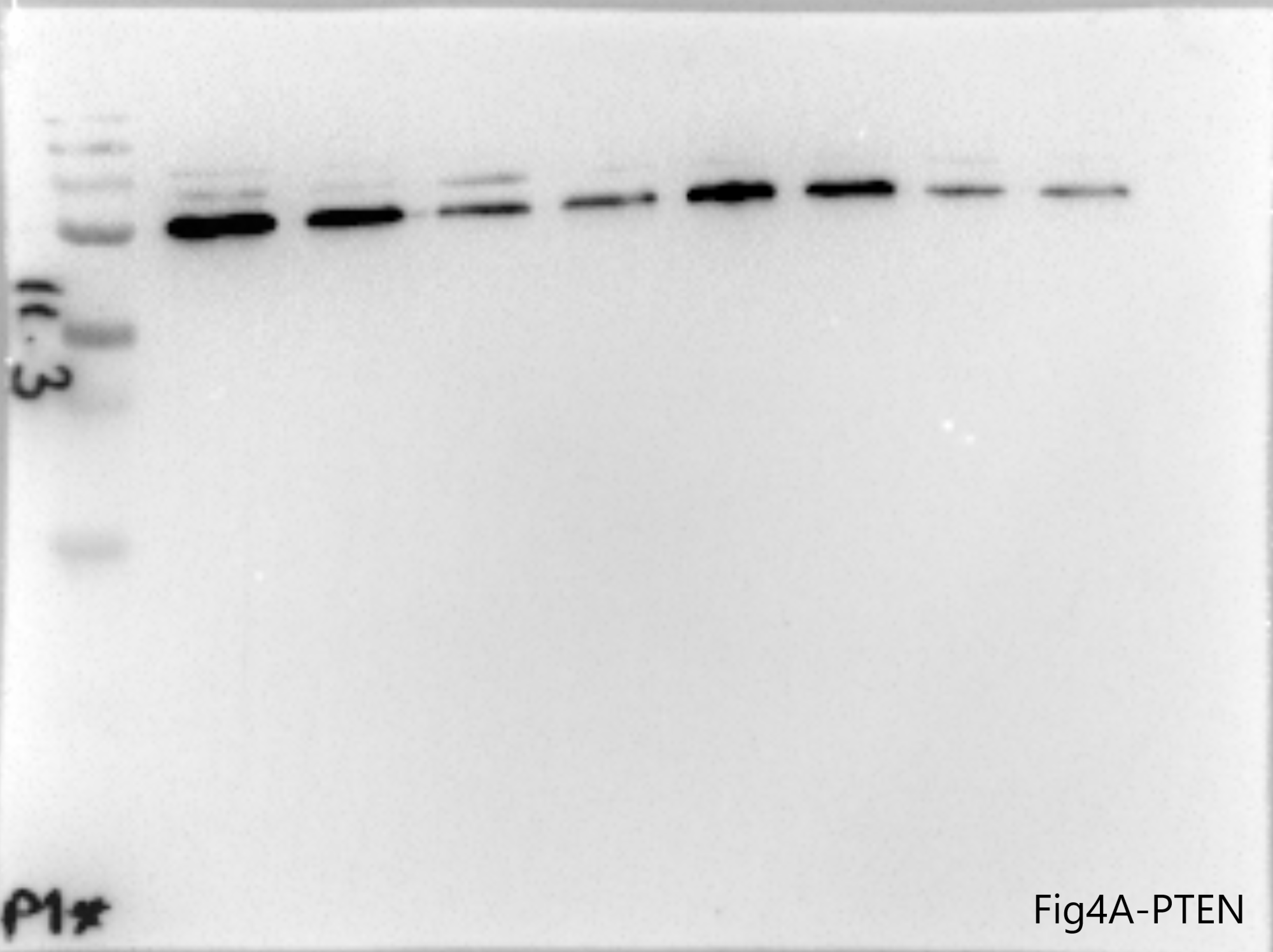

Fig4A-PTEN

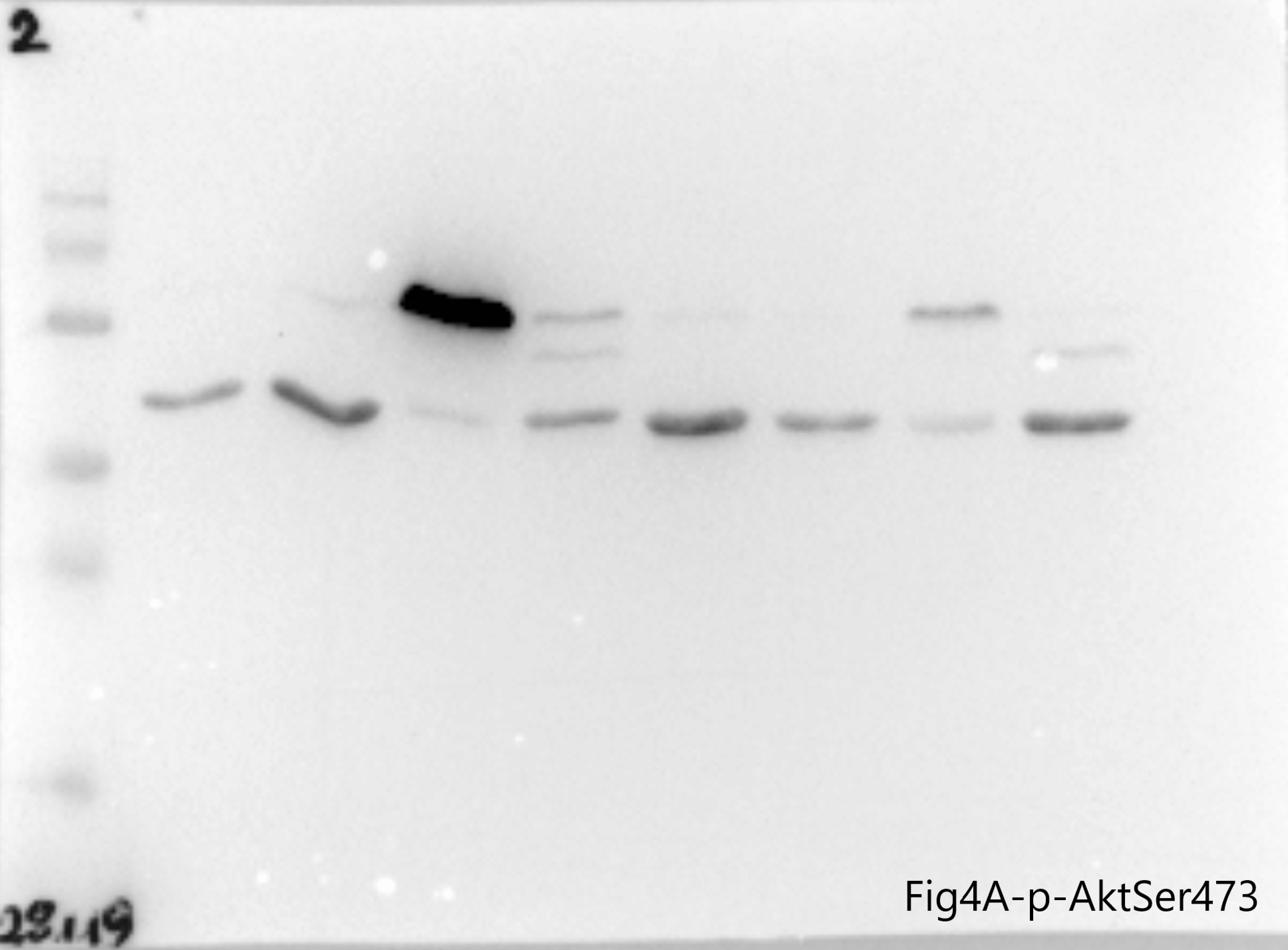

Fig4A-p-AktSer473

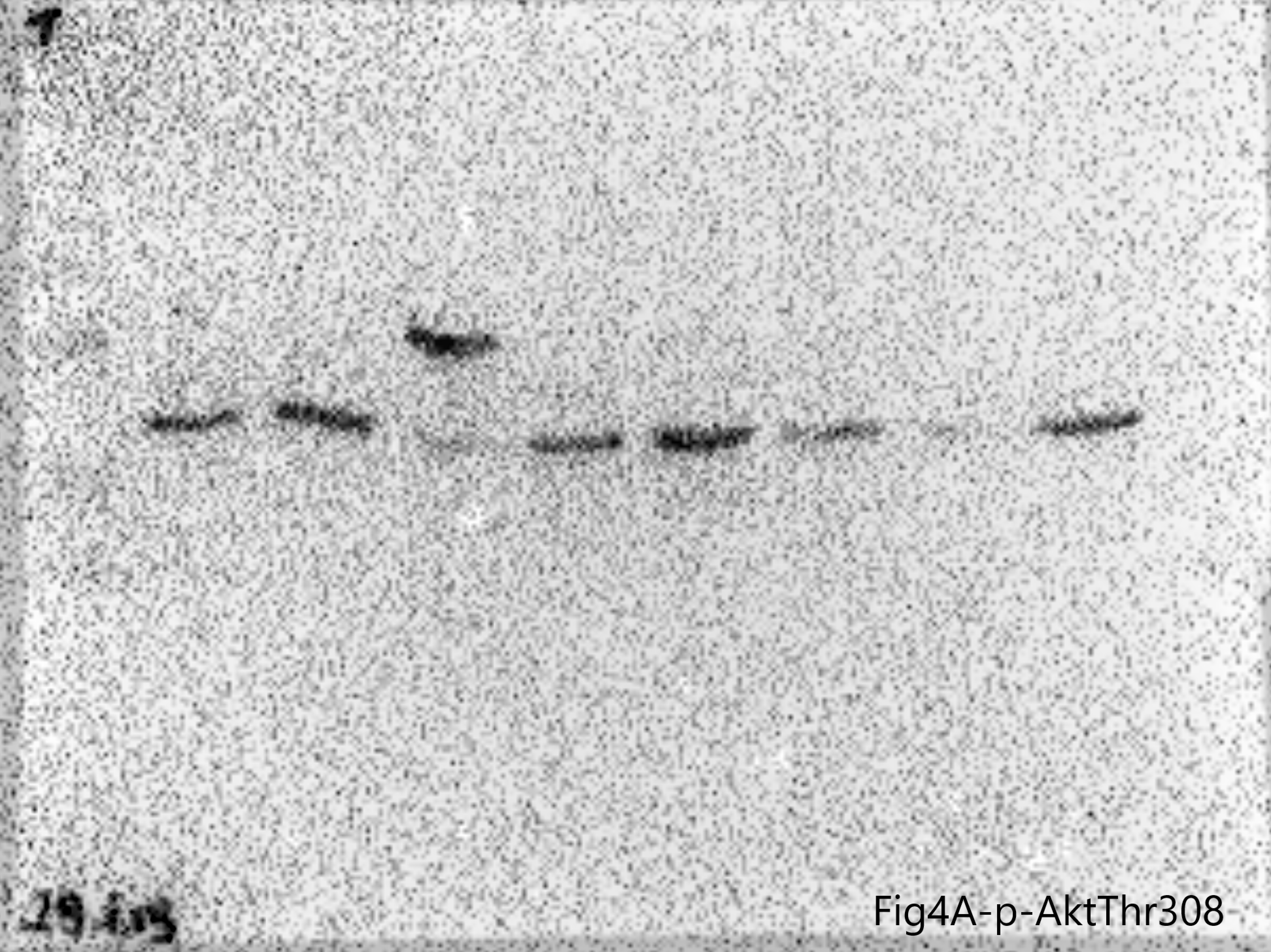

Fig4A-p-AktThr308

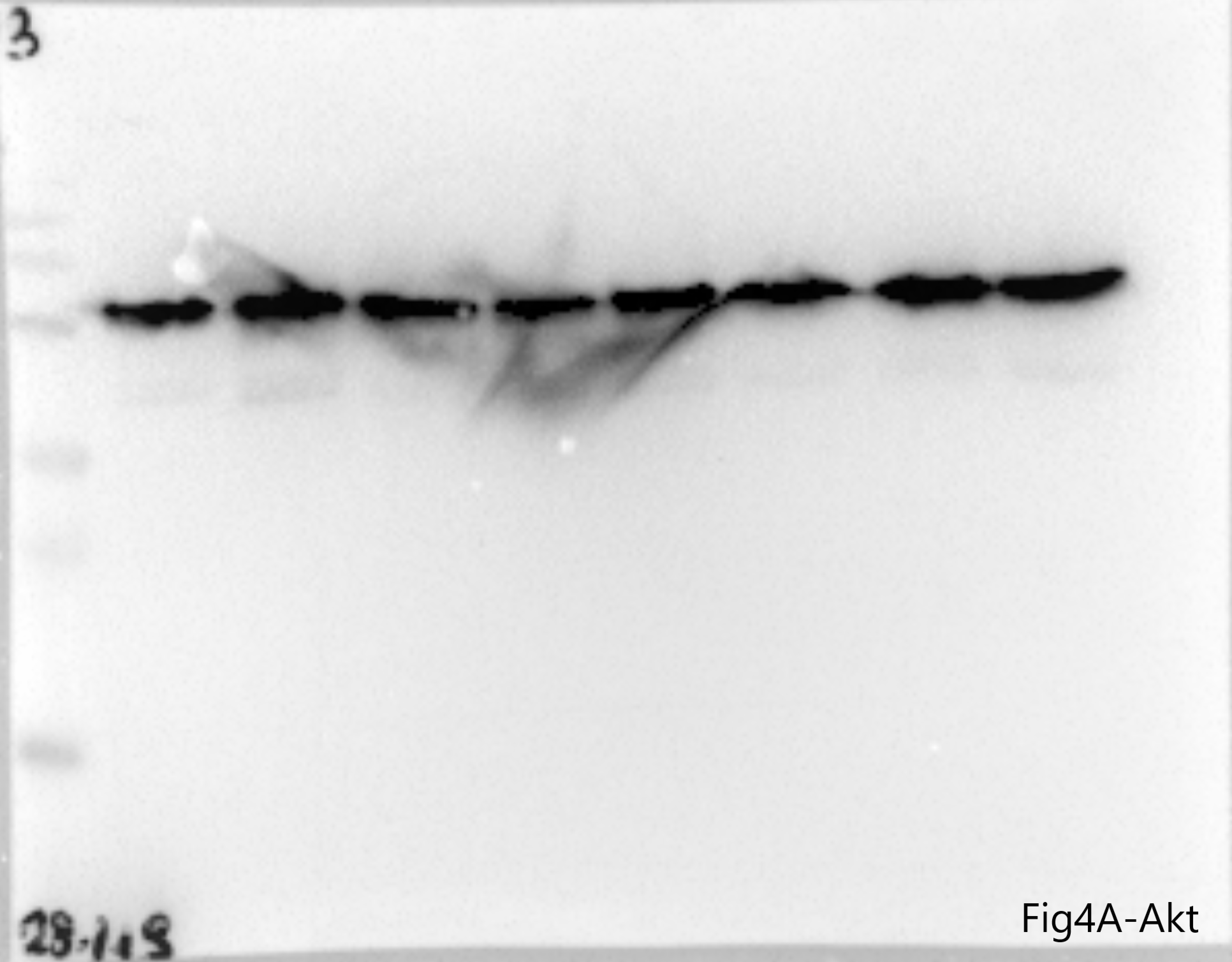

Fig4A-Akt

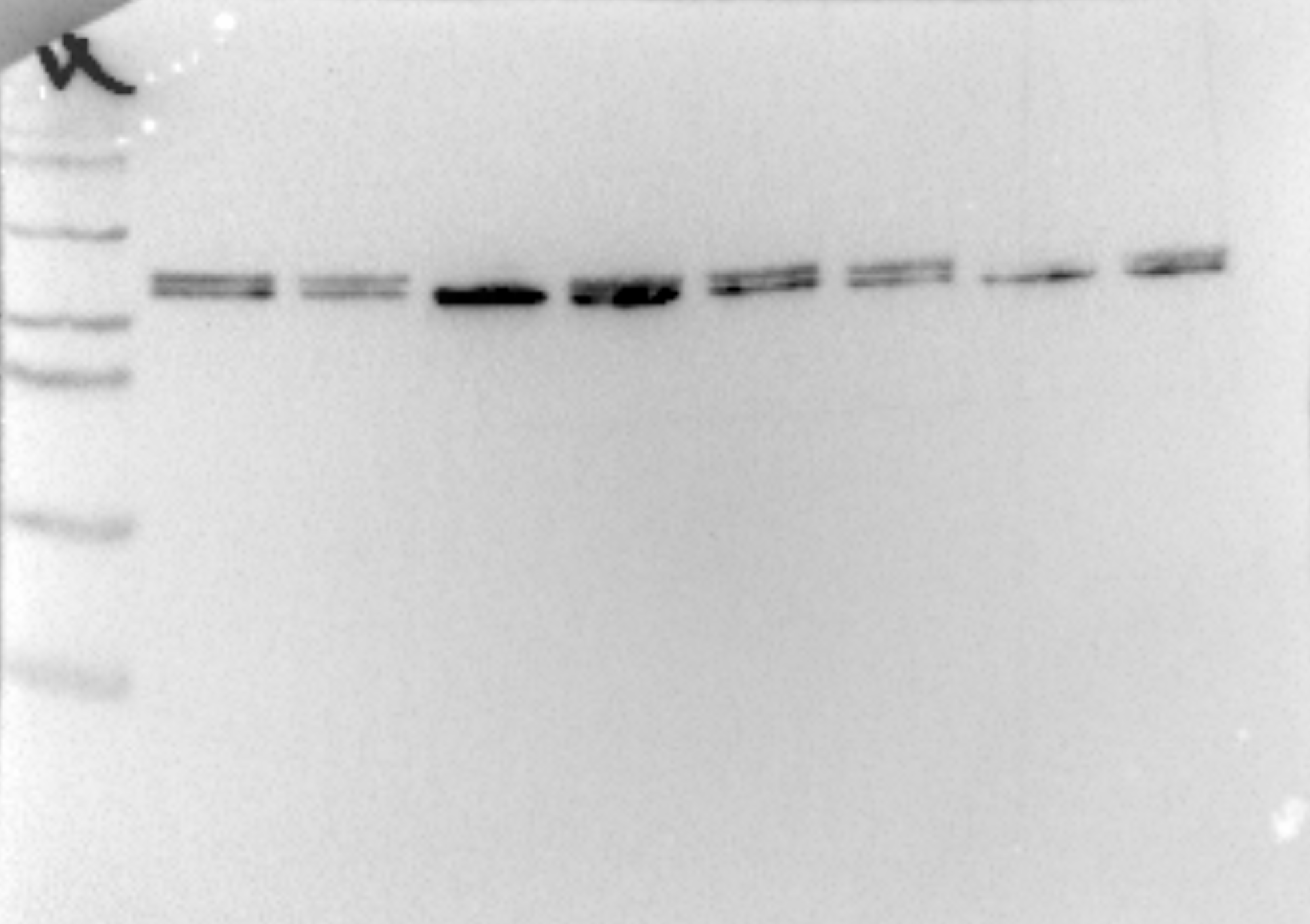

Fig 4A-p-p38

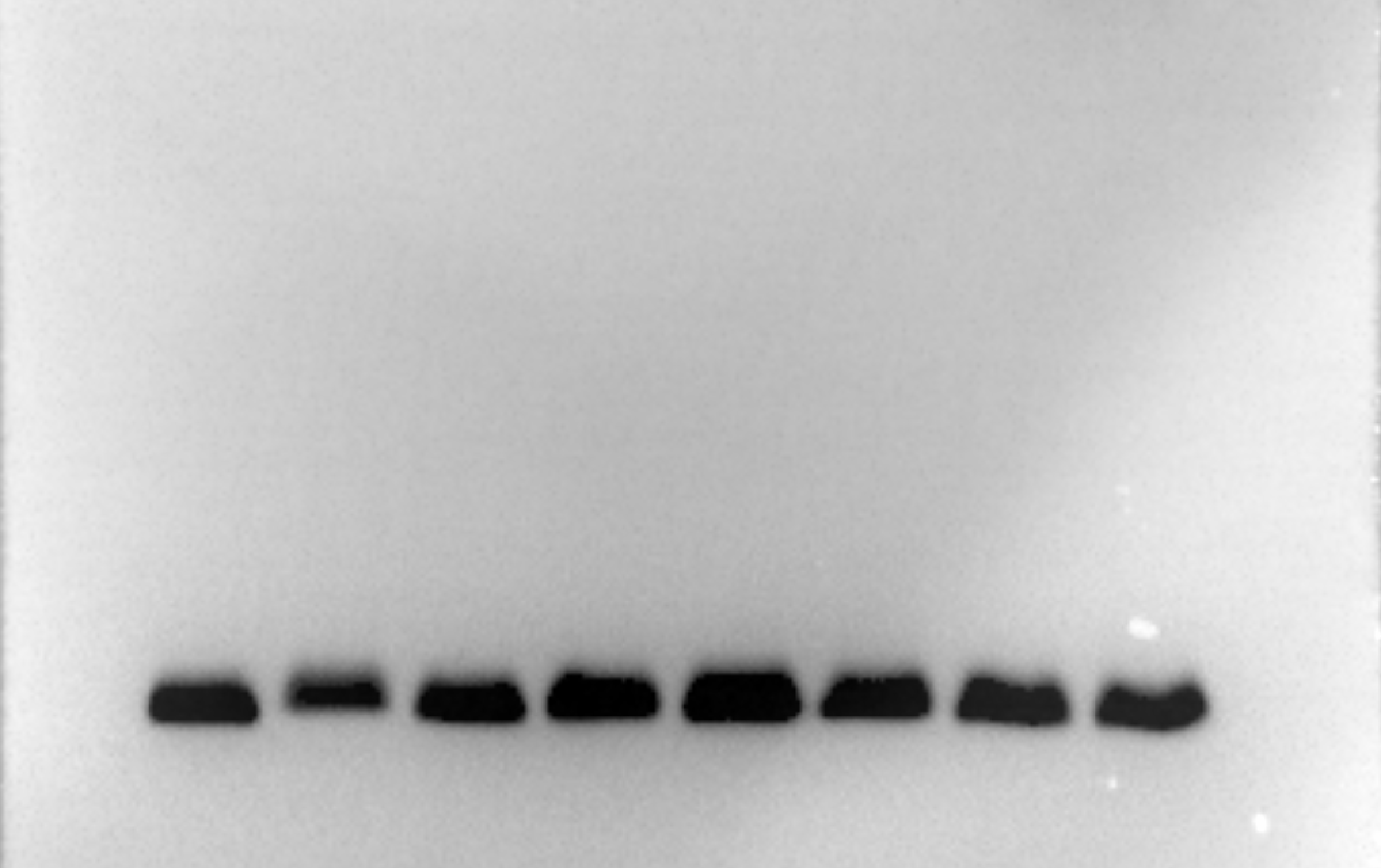

Fig4A-GAPDH

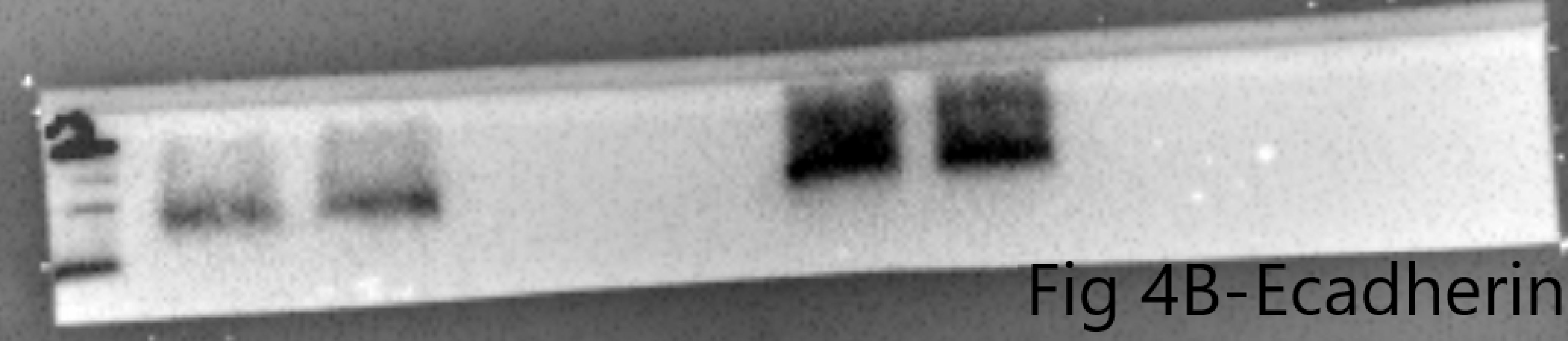

Fig 4B-Ecadherin

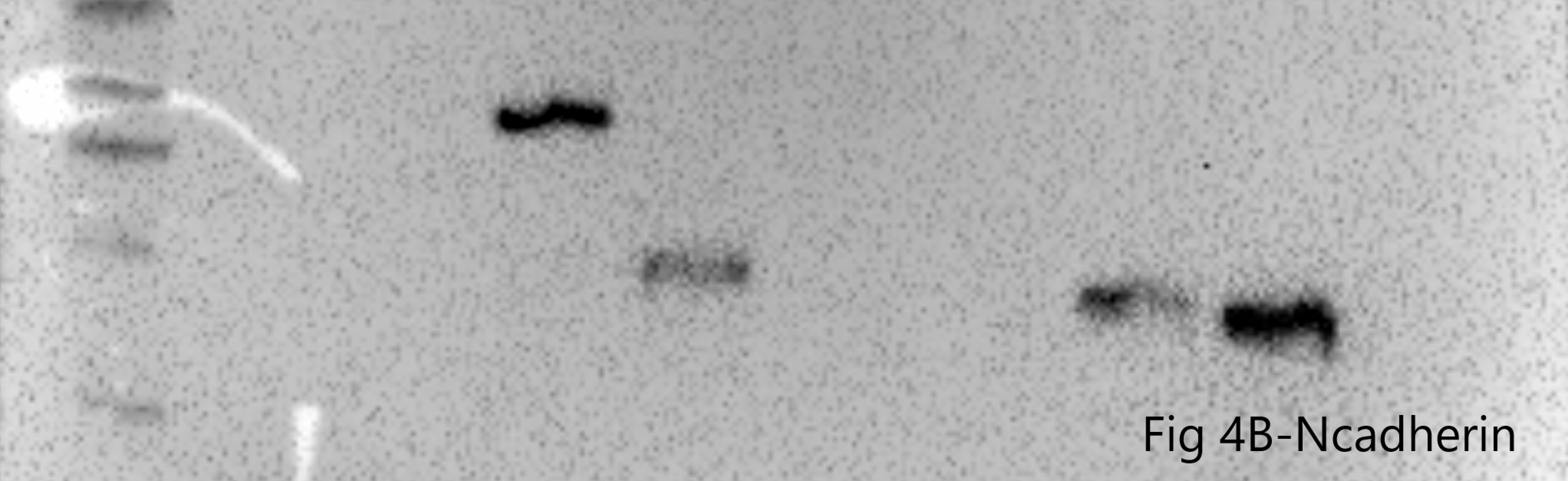

Fig 4B-Ncadherin

Fig 4B-ZEB1

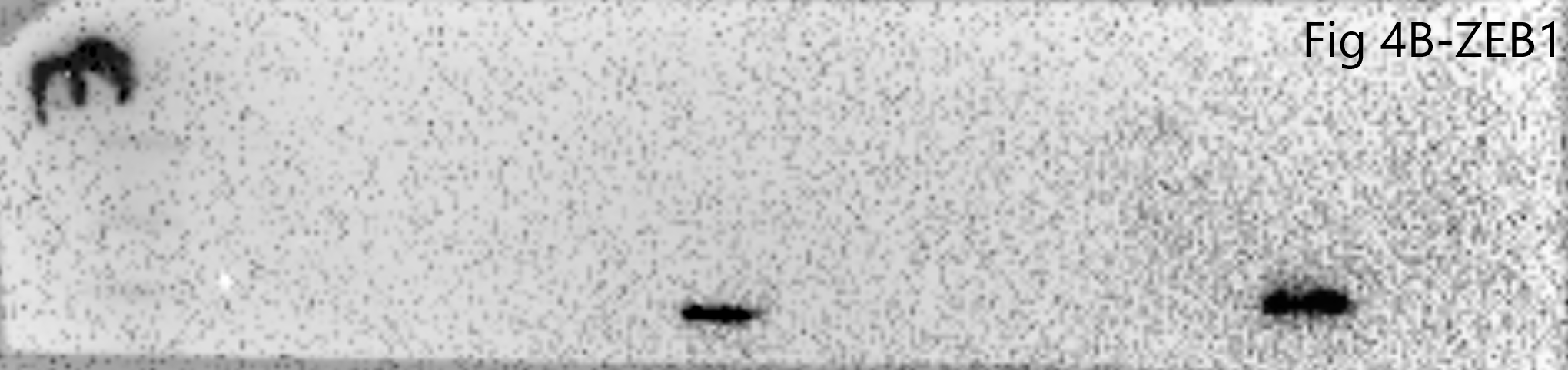

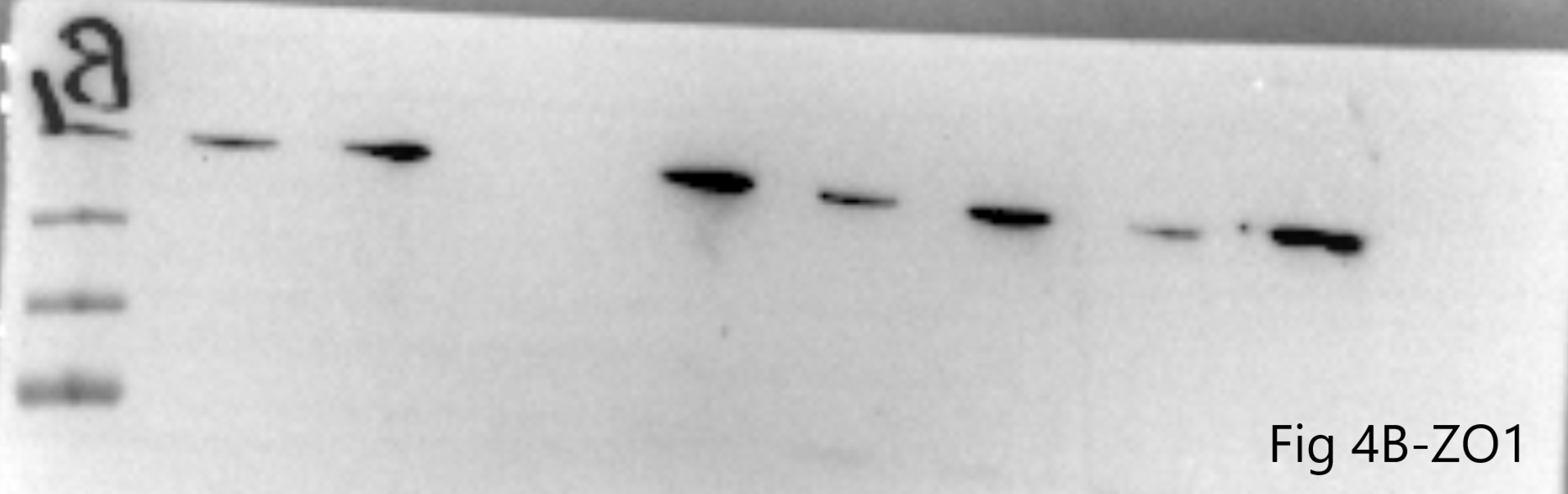

Fig 4B-ZO1

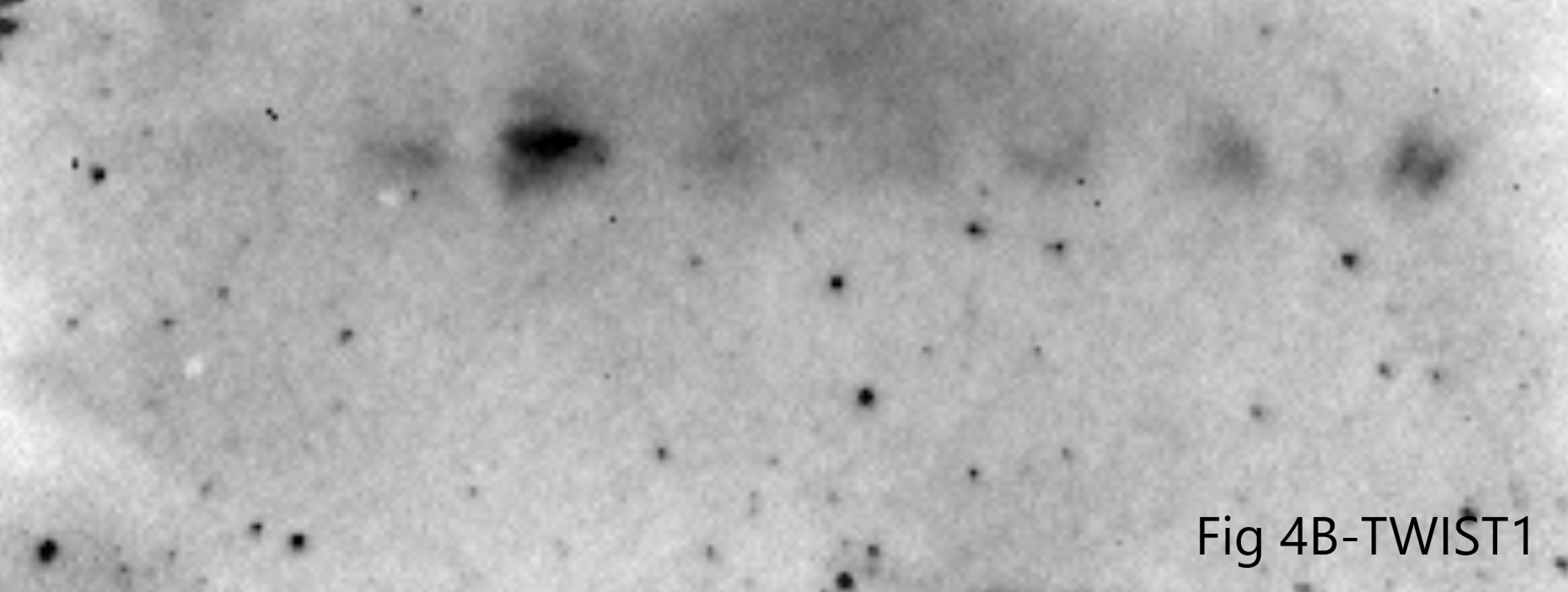

Fig 4B-TWIST1

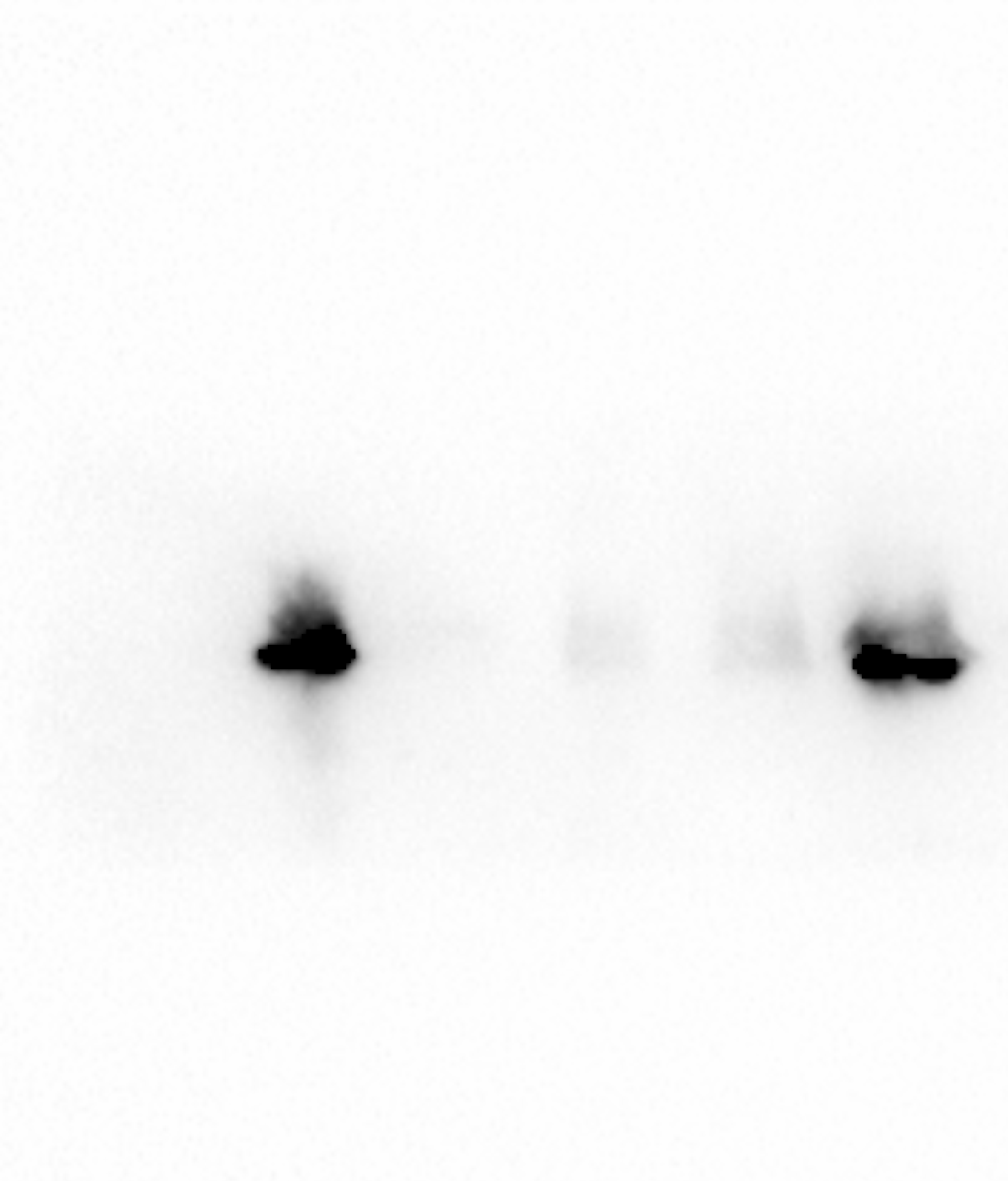

Fig 4B-p-FAK Tyr397

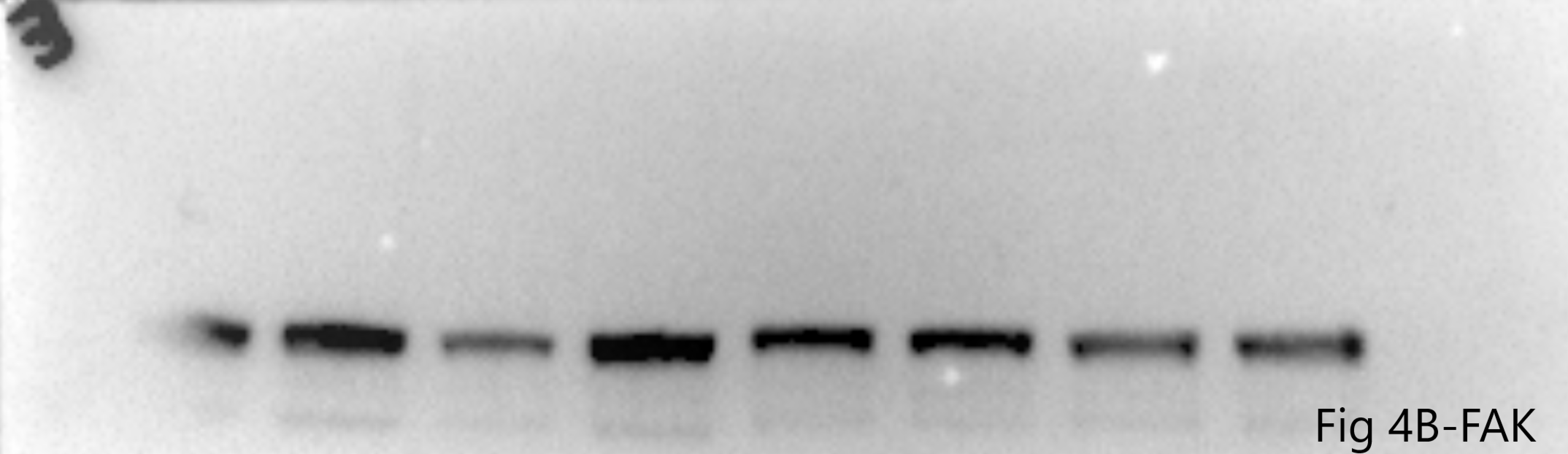

Fig 4B-FAK

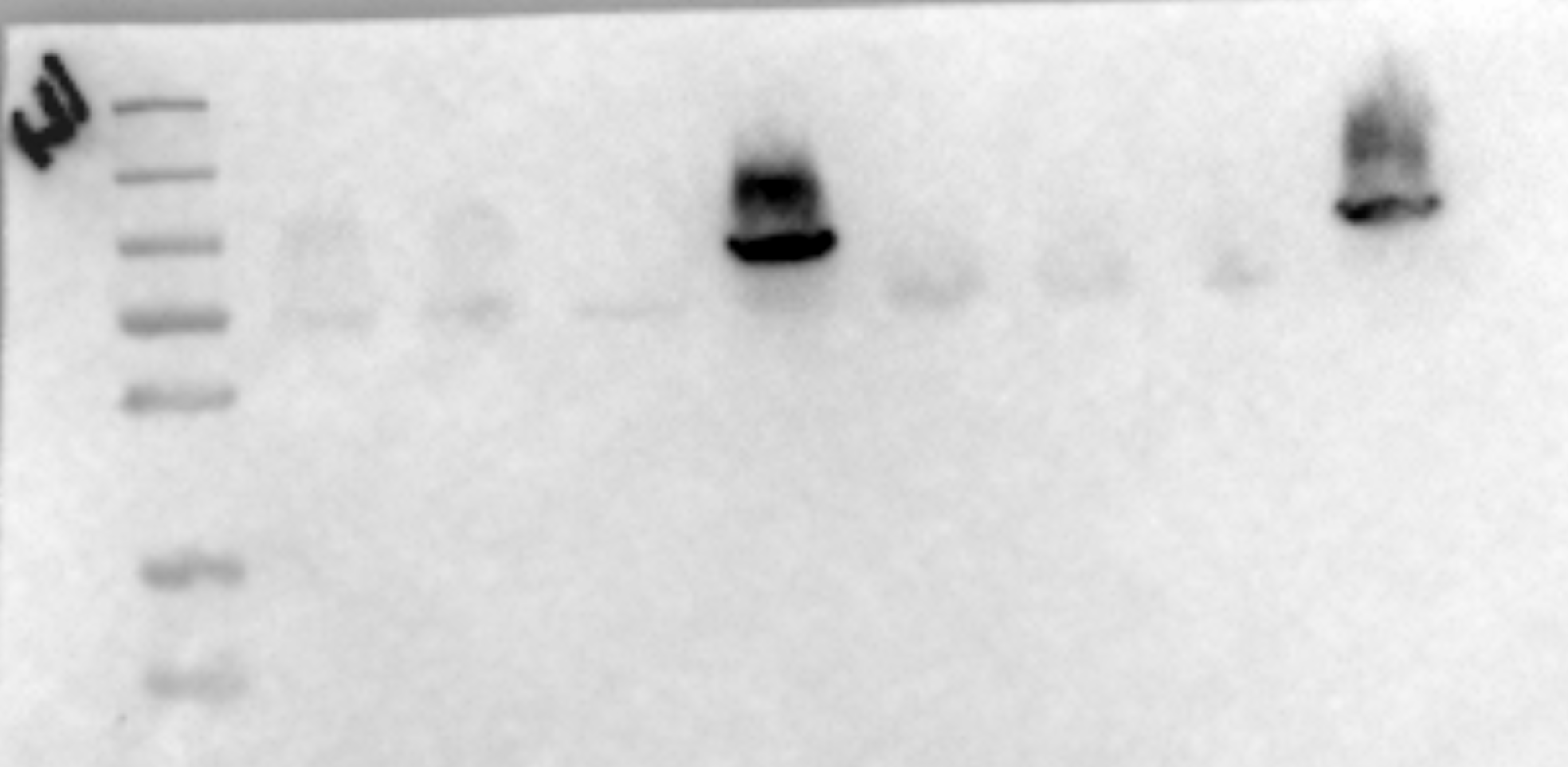

Fig 4B- paxillin

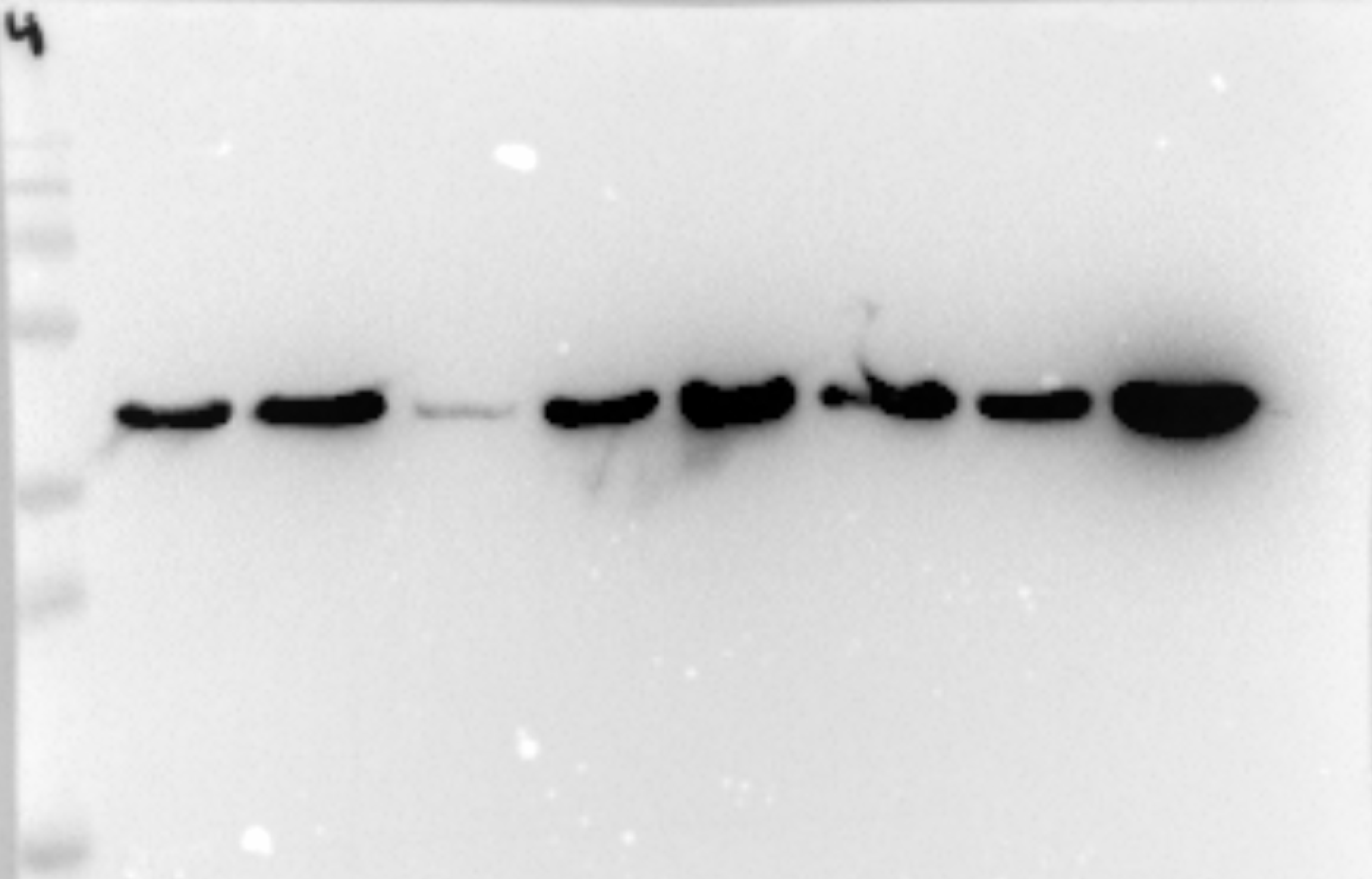

Fig4B- Betaactin

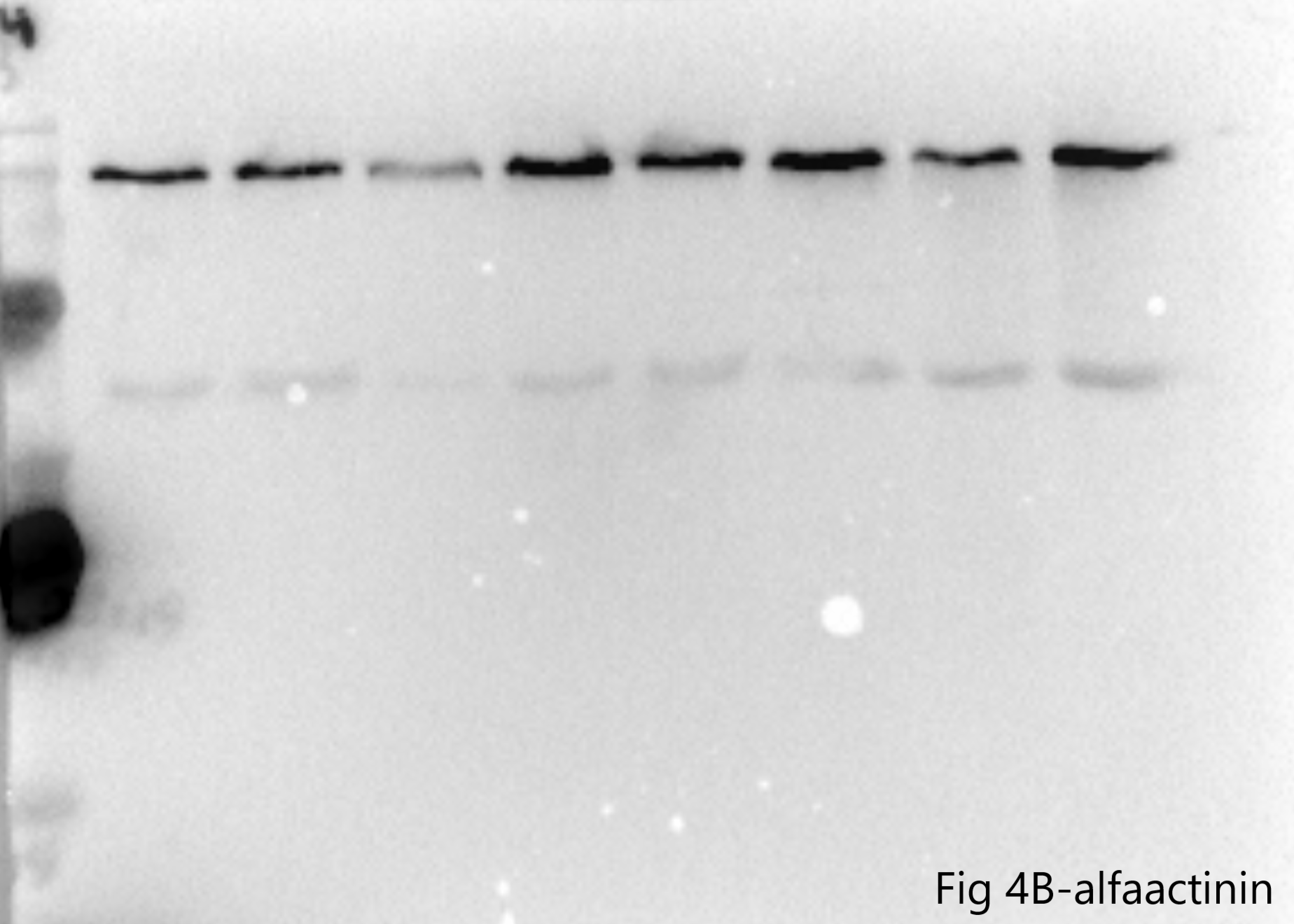

Fig 4B-alfaactinin

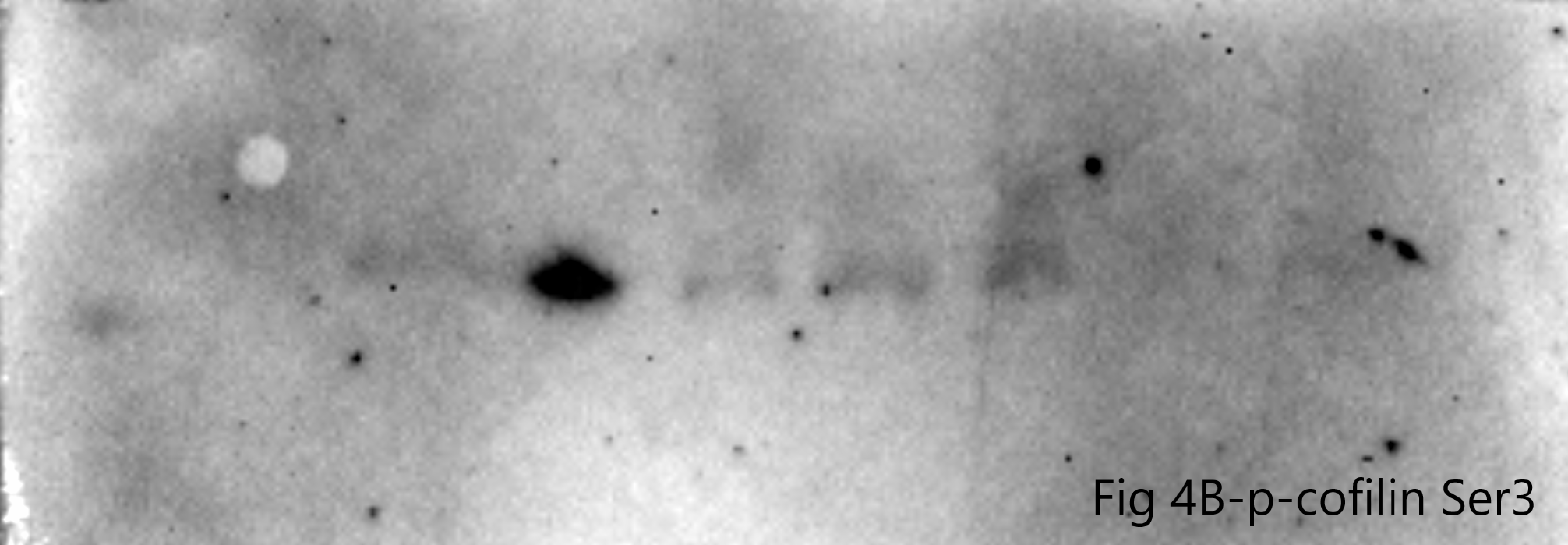

Fig 4B-p-cofilin Ser3

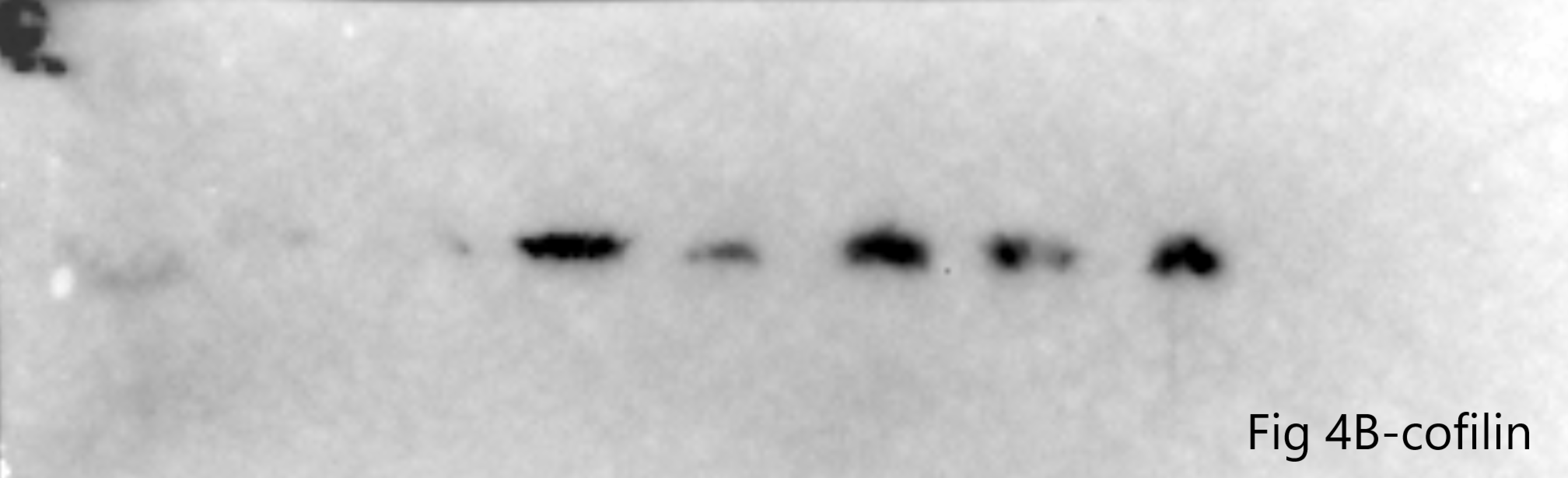

Fig 4B-cofilin

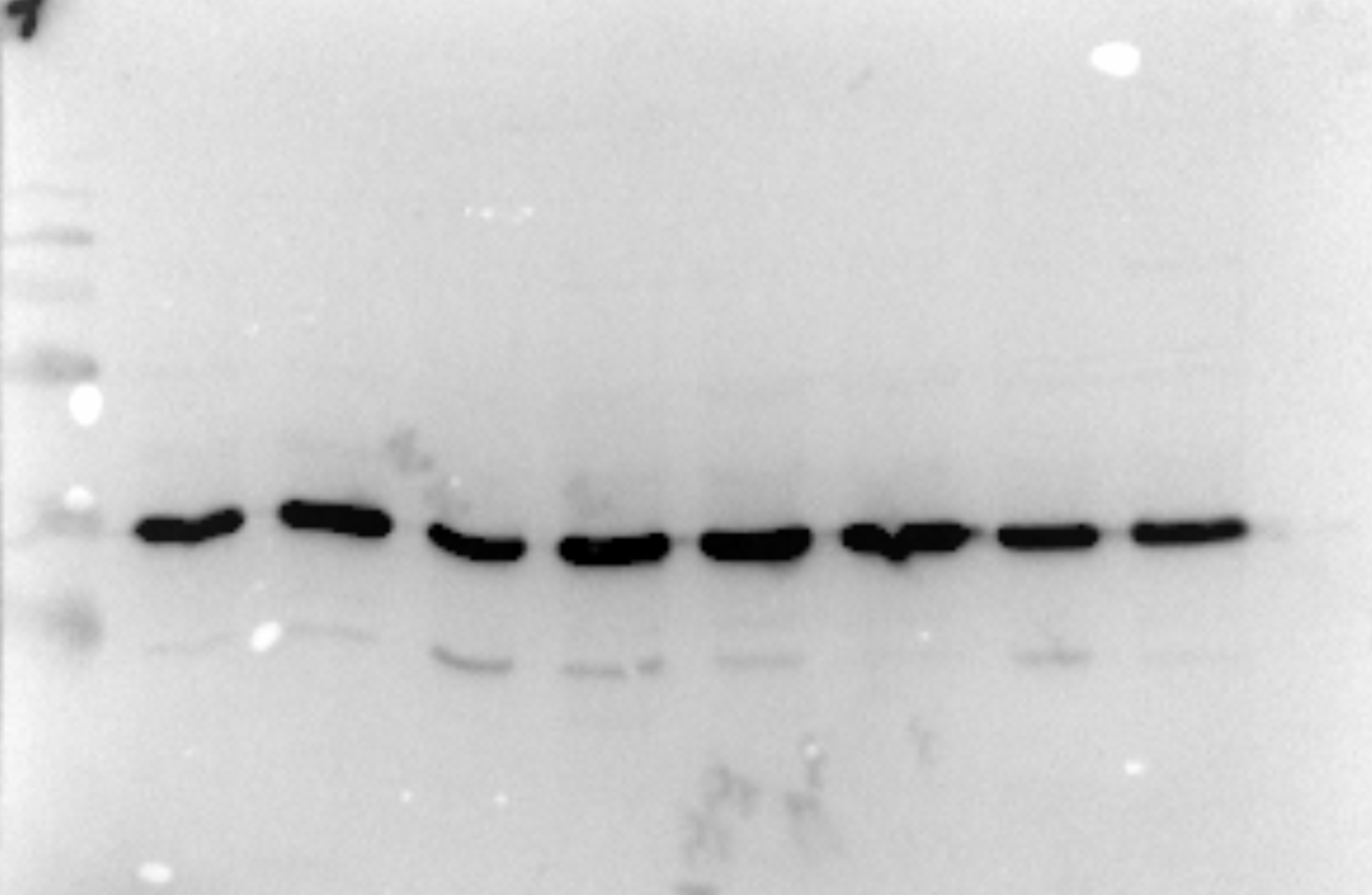

Fig 4B-GAPDH

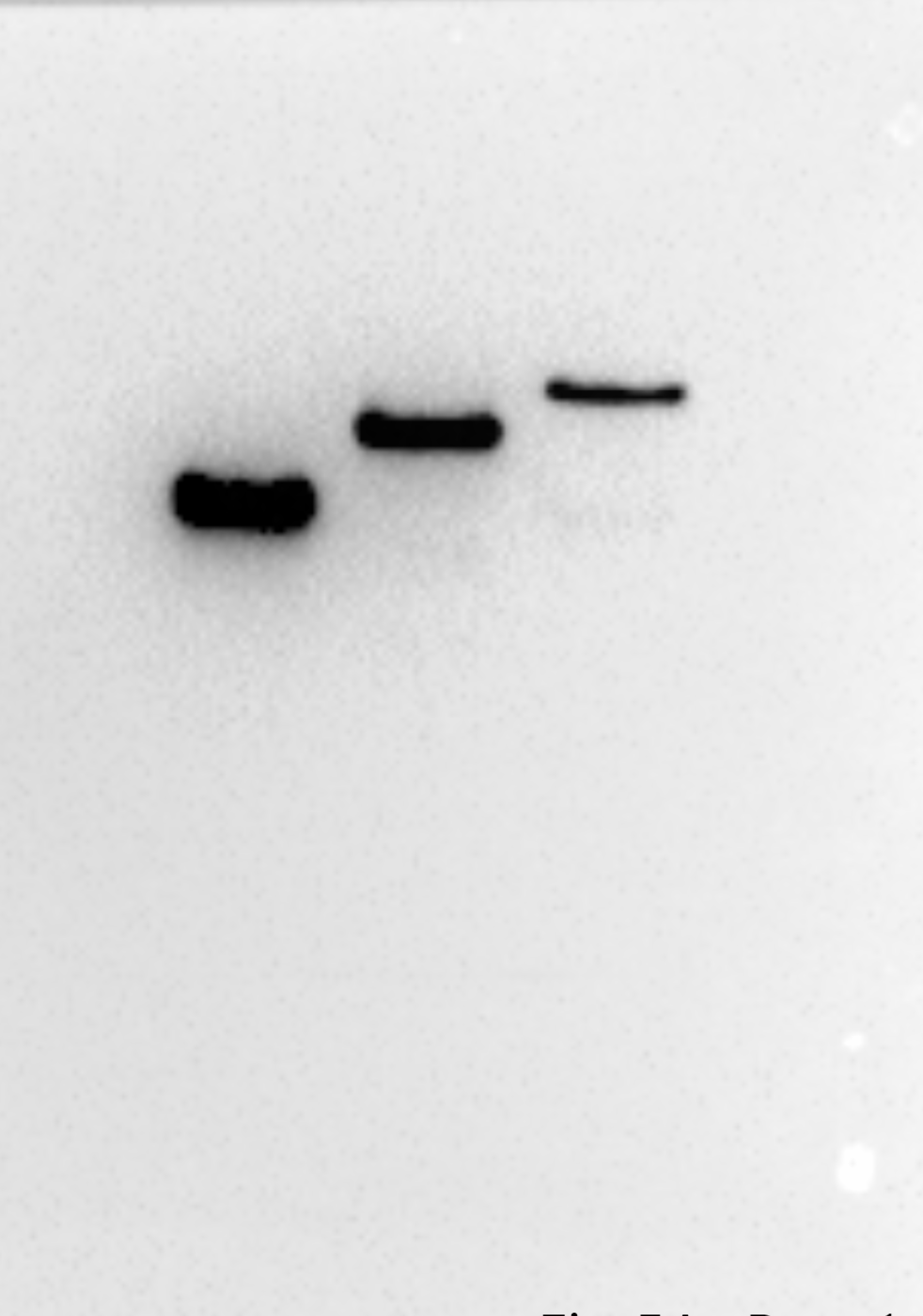

Fig 5A- Bag-1

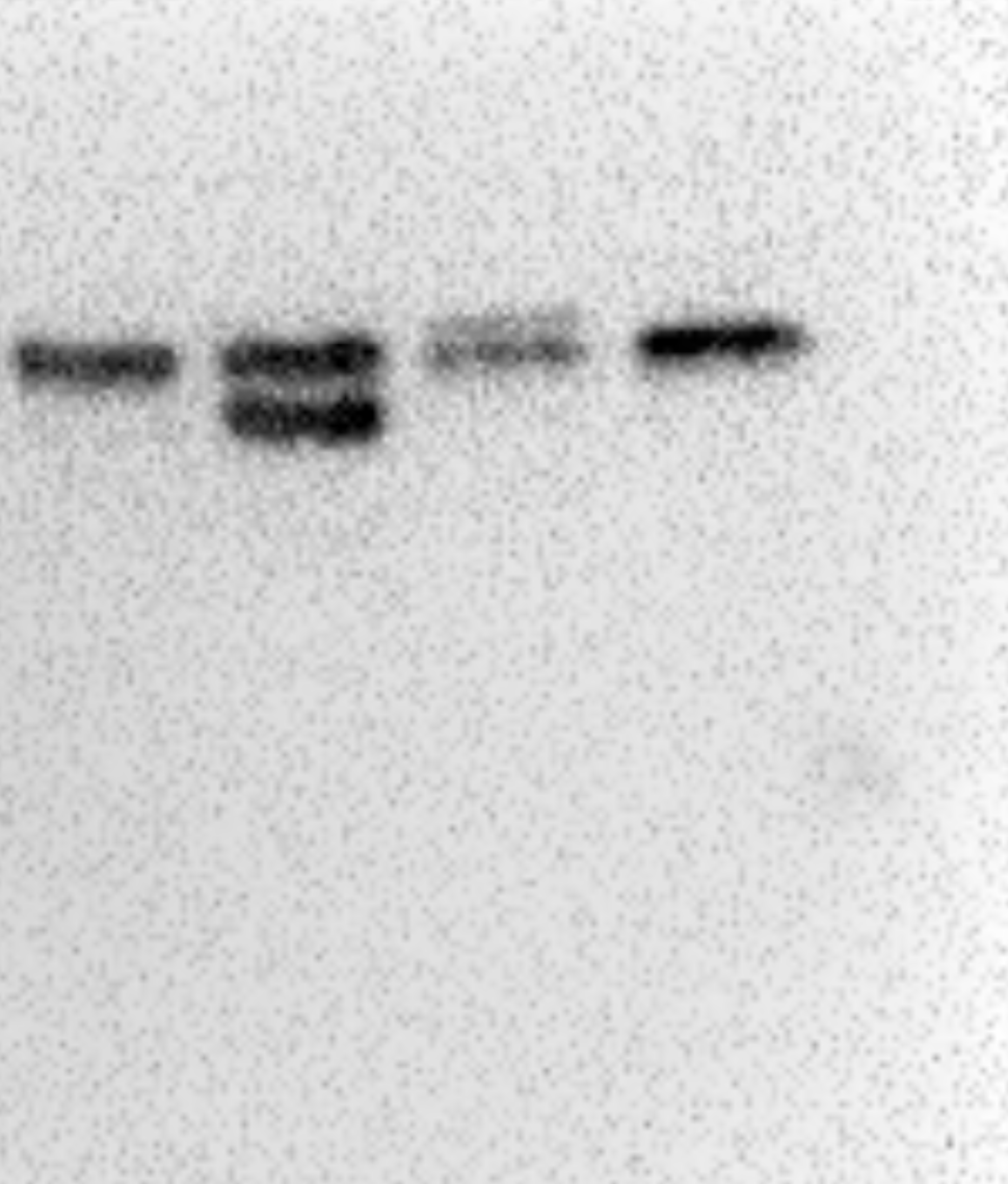

Fig 5A- pPI3K

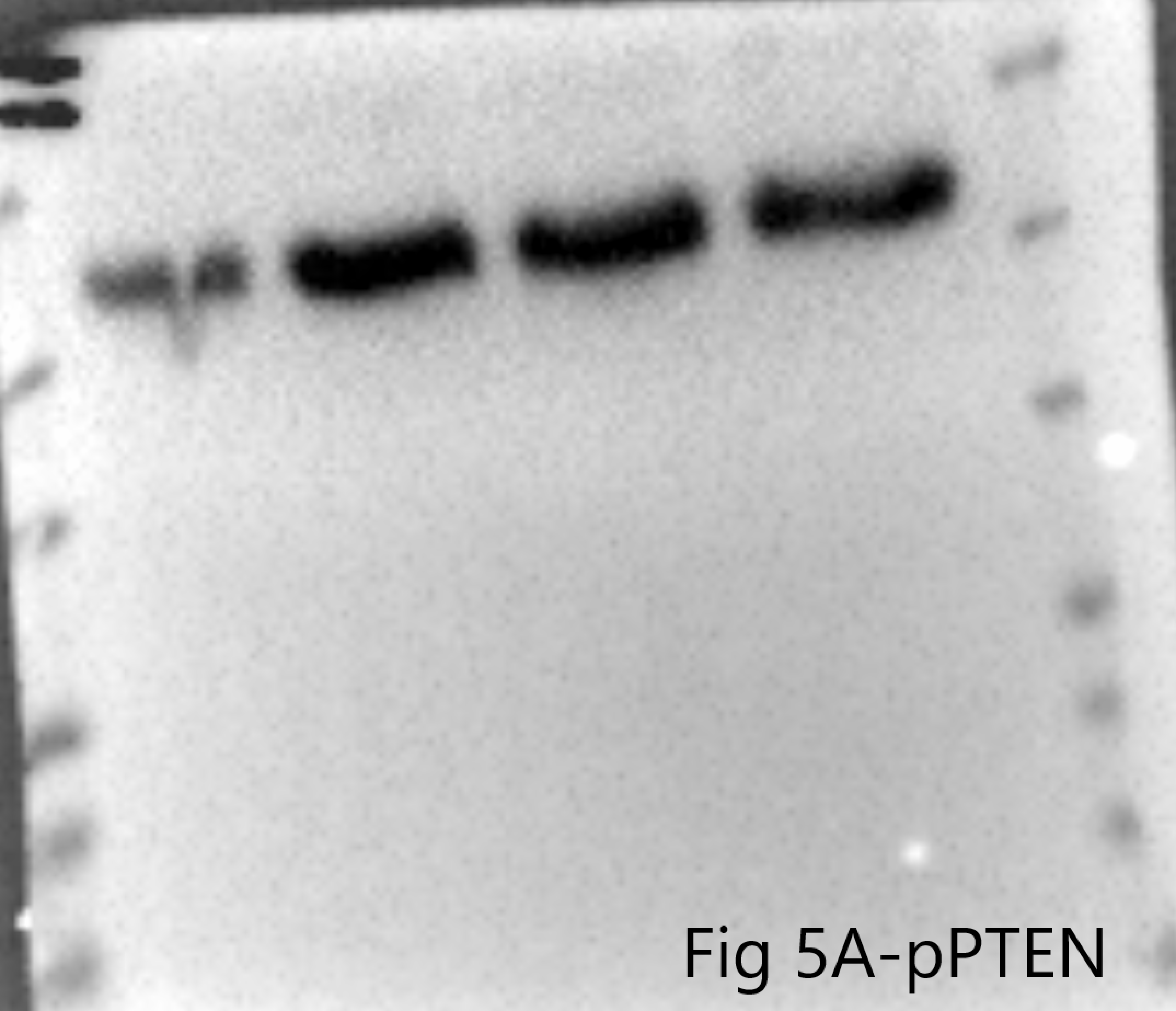

Fig 5A-pPTEN

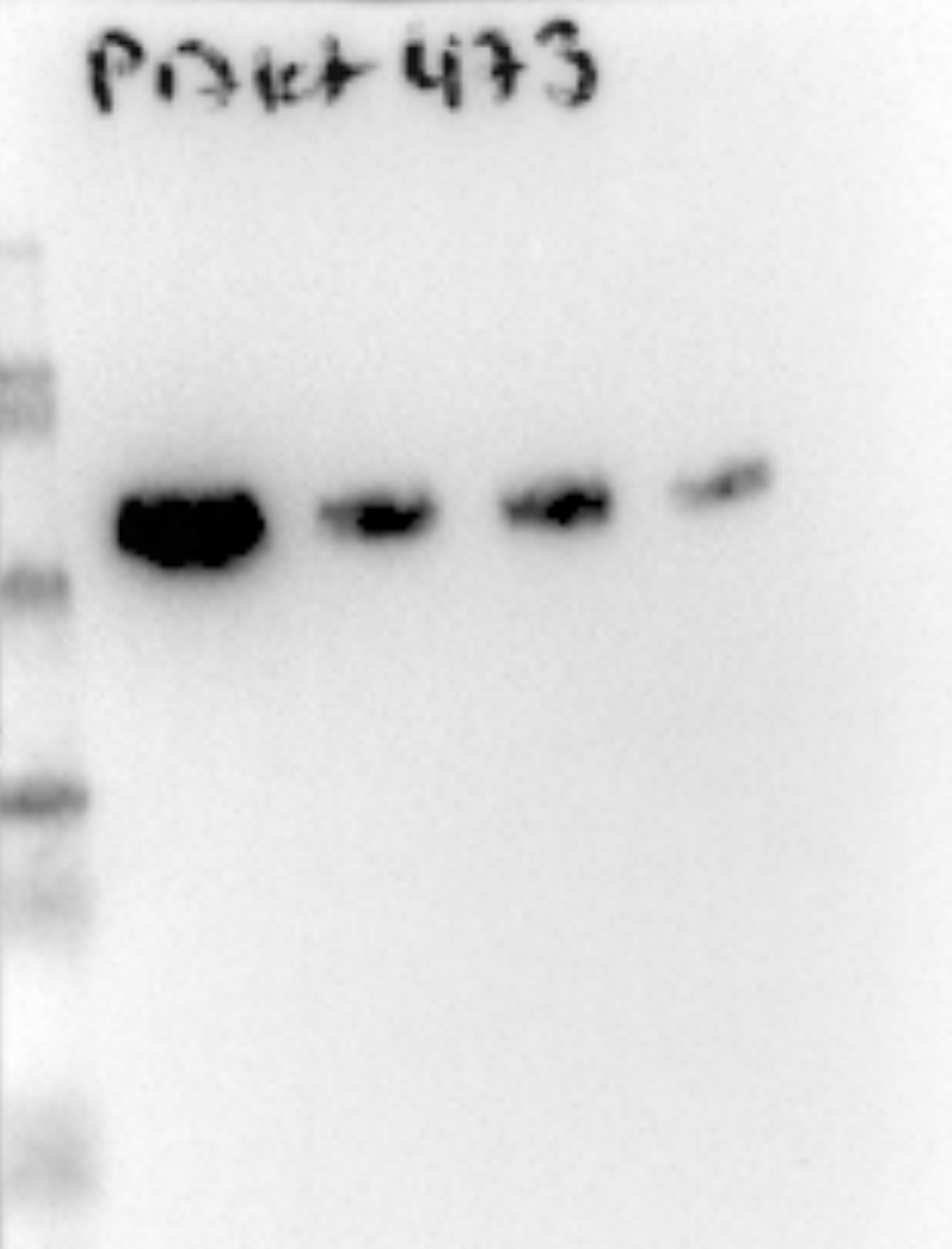

Fig 5A-p-Akt Ser473

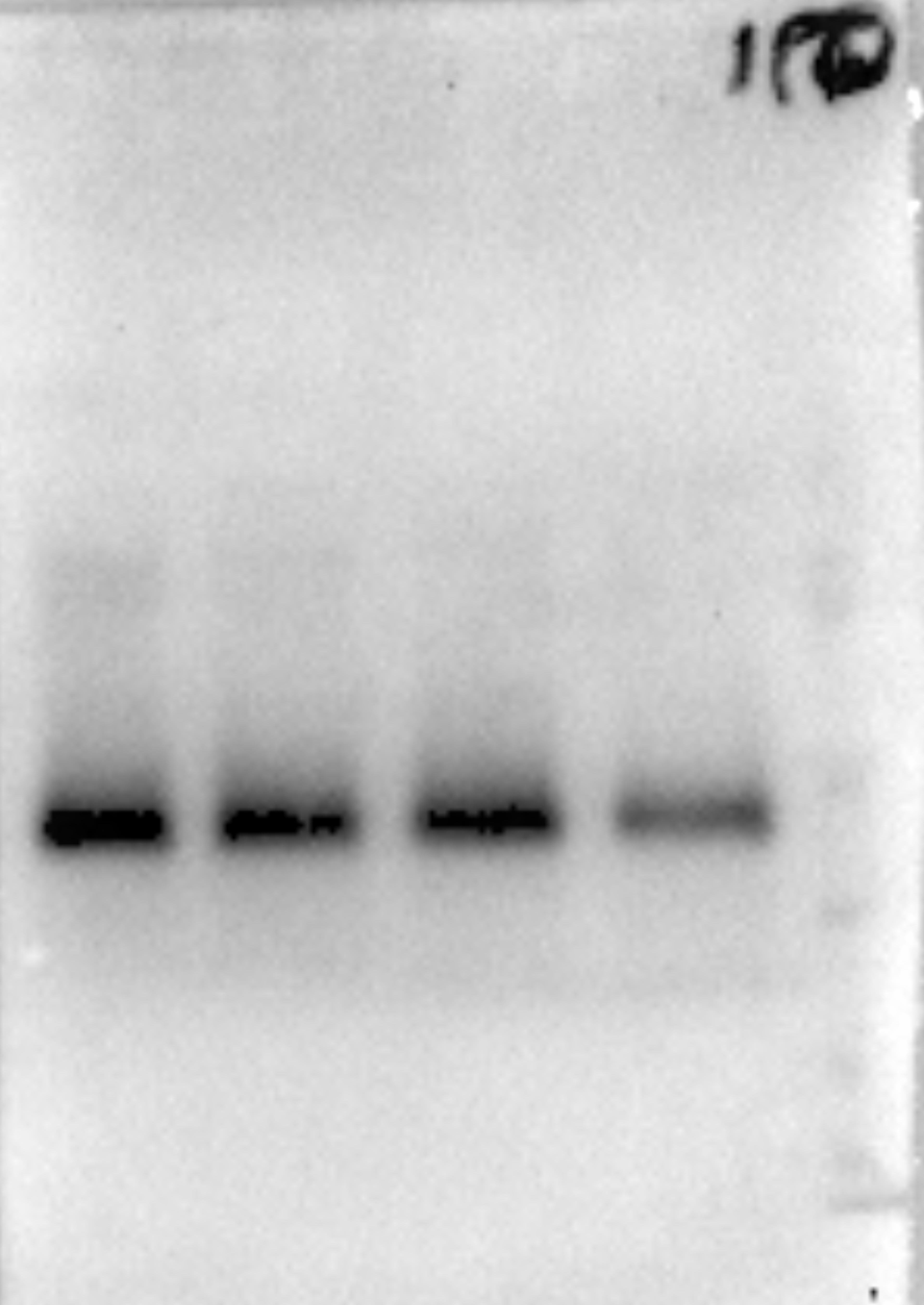

Fig 5A-p-Akt Thr308

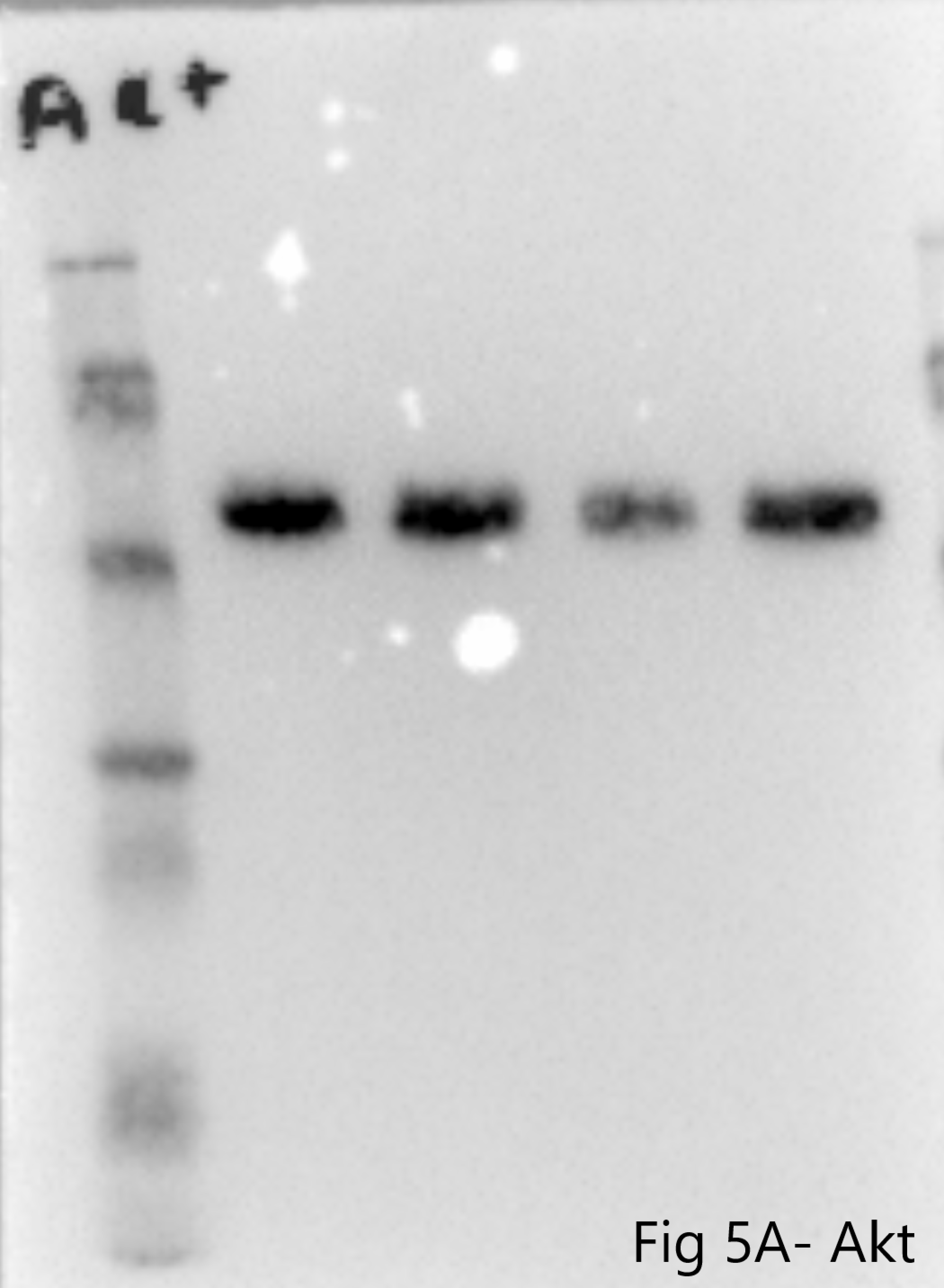

Fig 5A- Akt

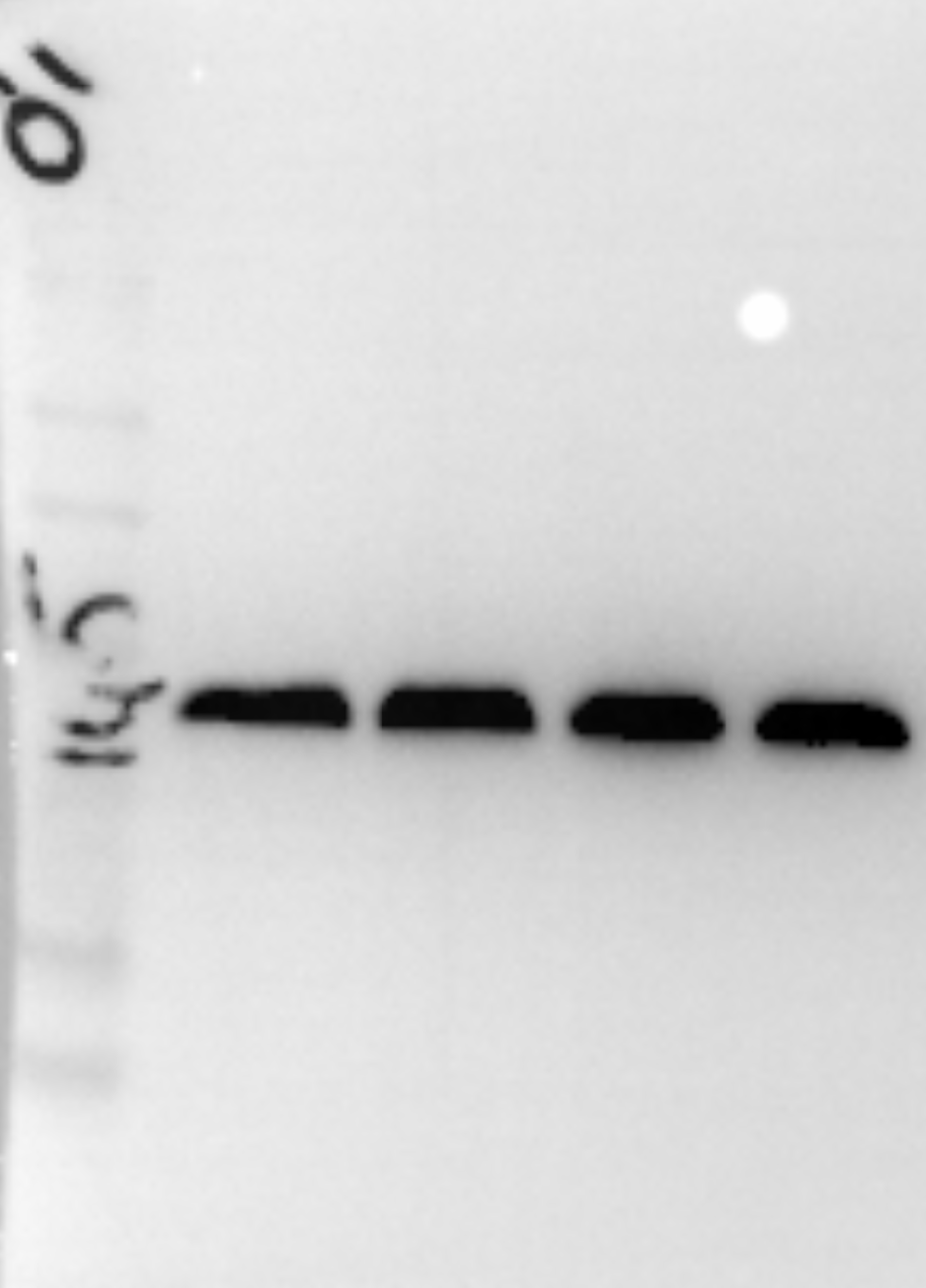

Fig 5A-GAPDH

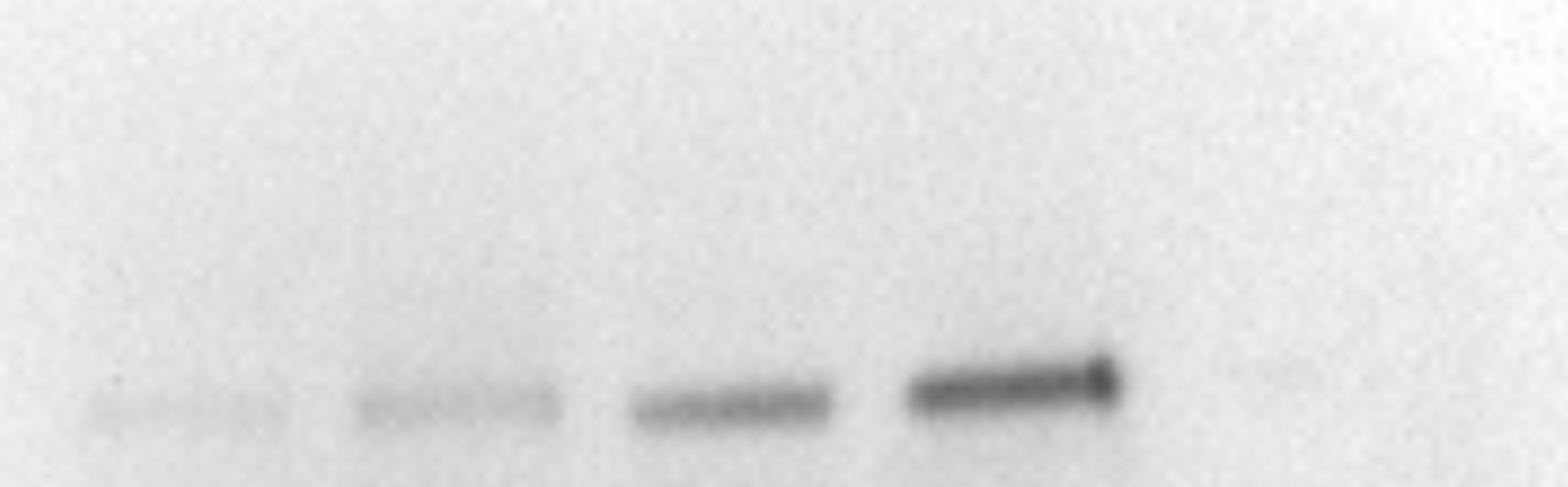

Fig 5B-Ecadherin

Fig 5B-Ncadherin

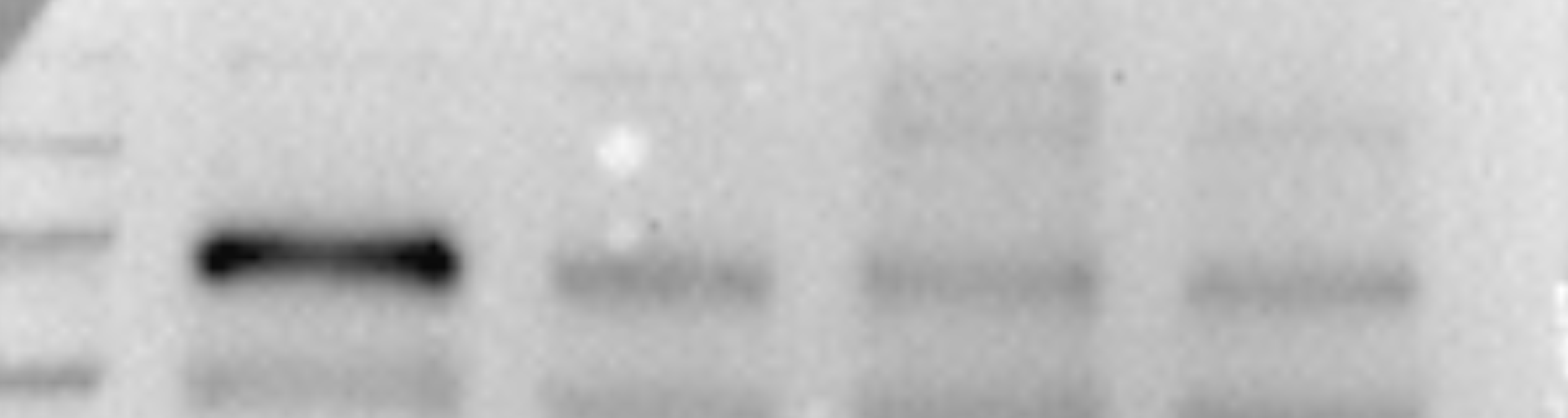

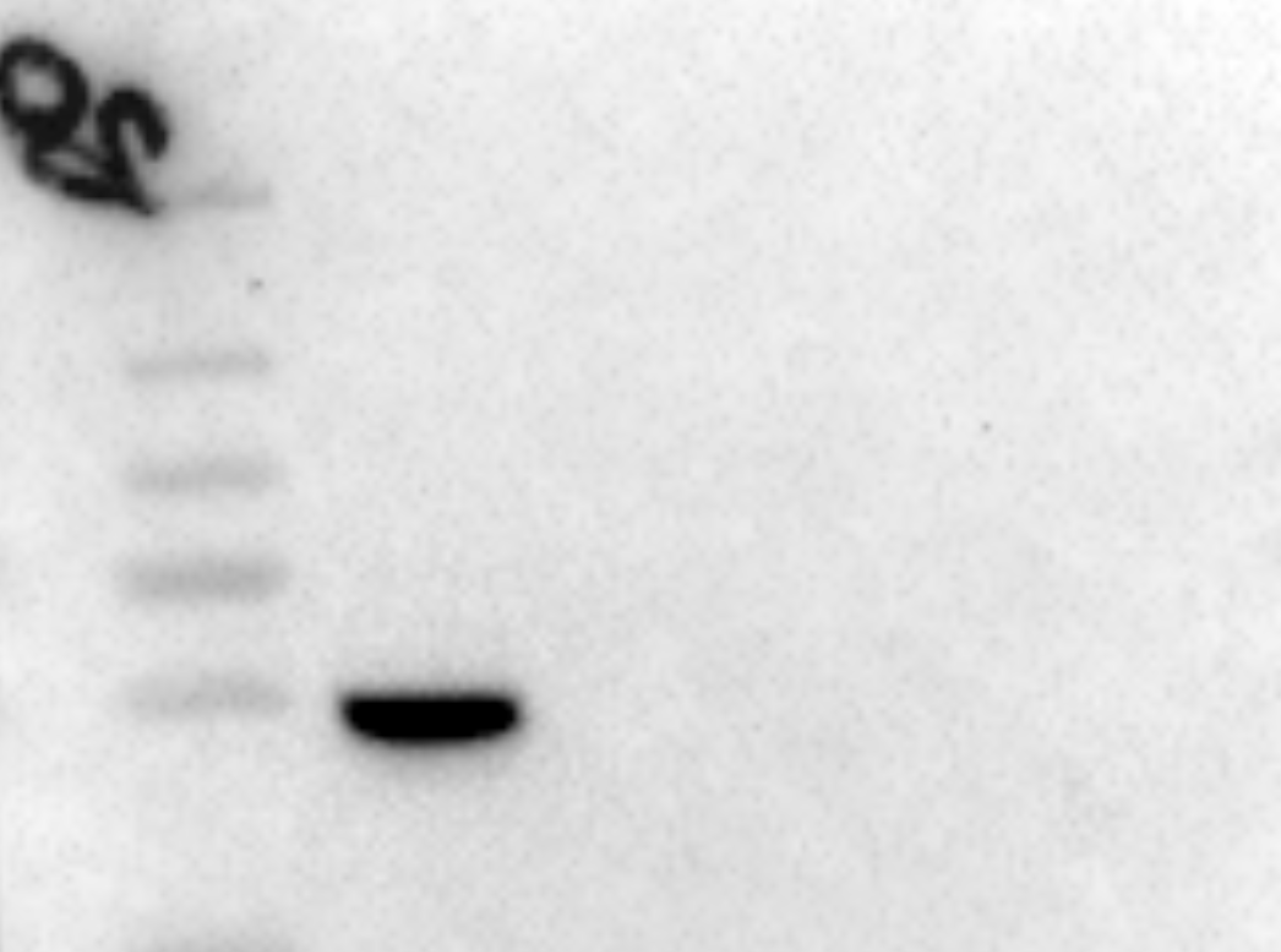

Fig 5B-Vimentin

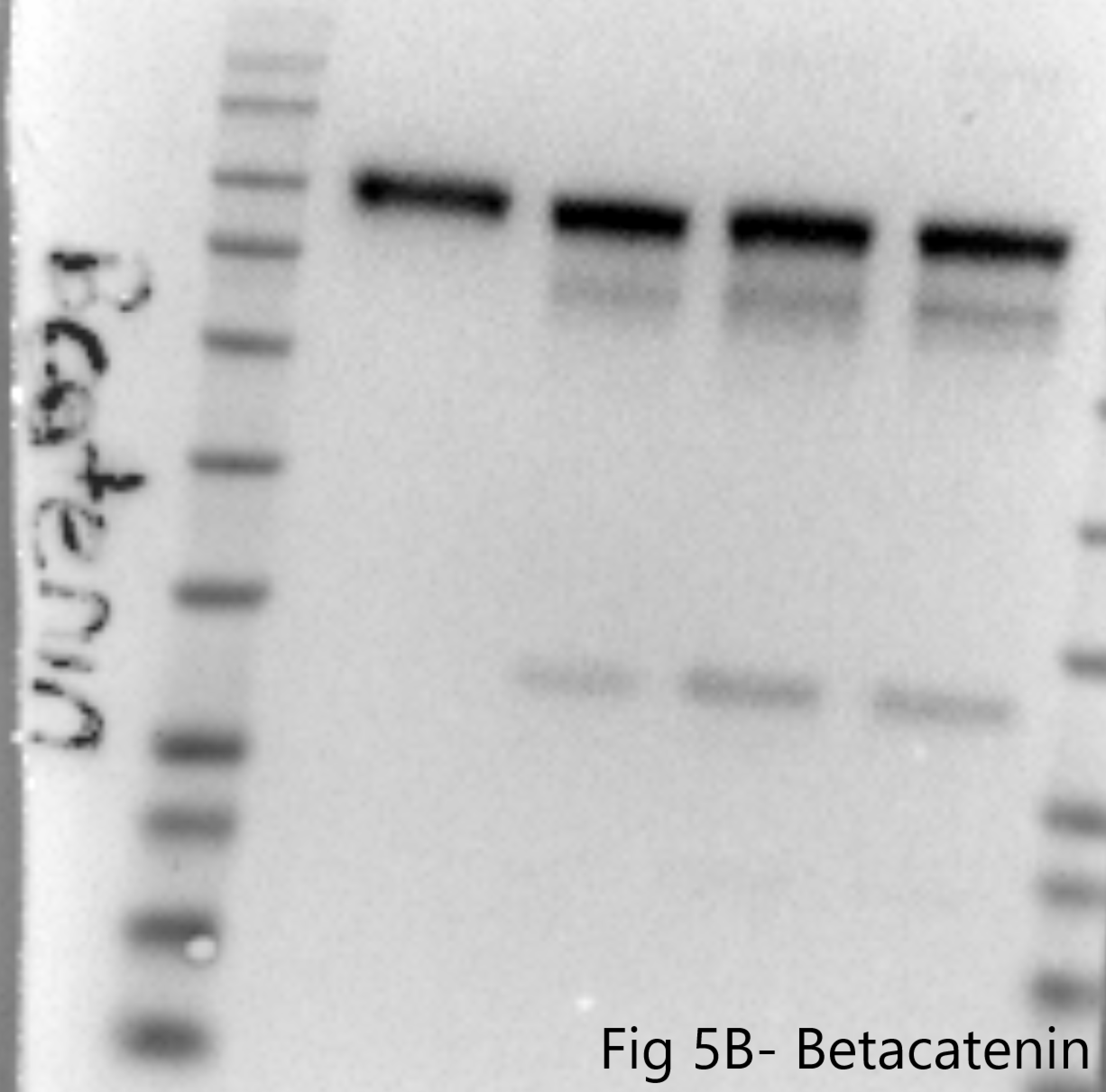

Fig 5B- Betacatenin

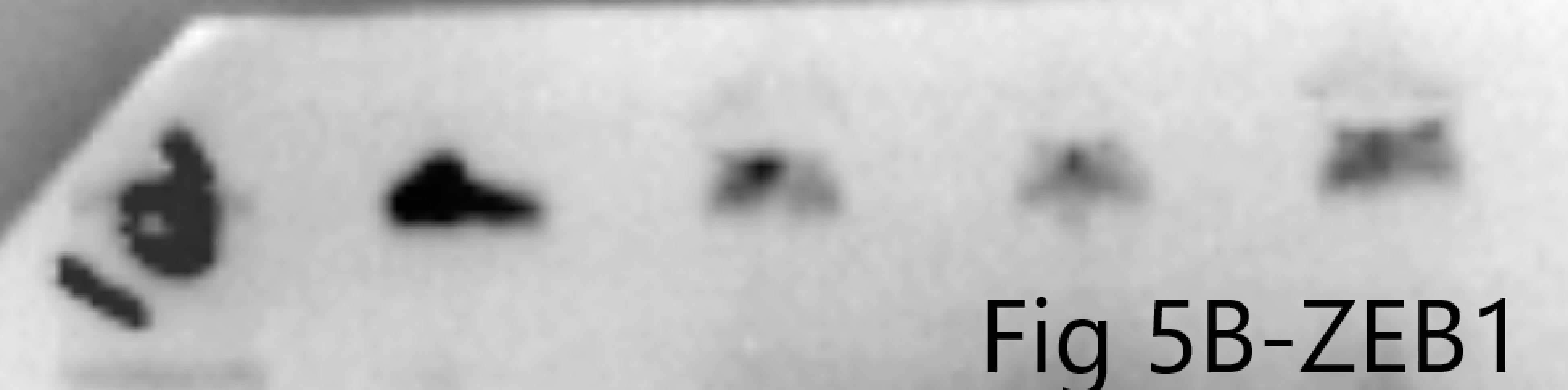

Fig 5B-ZEB1

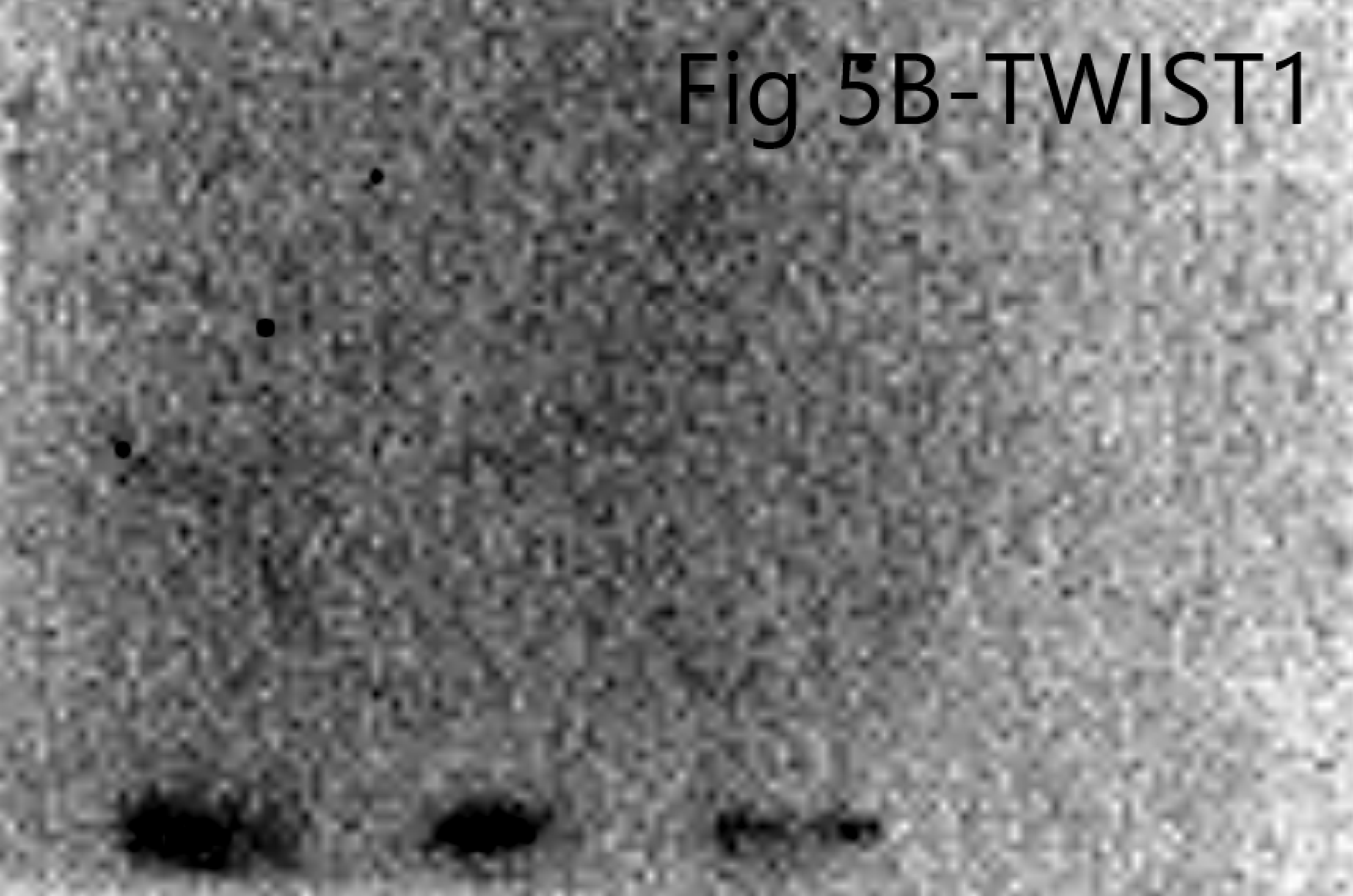

Fig 5B-TWIST1

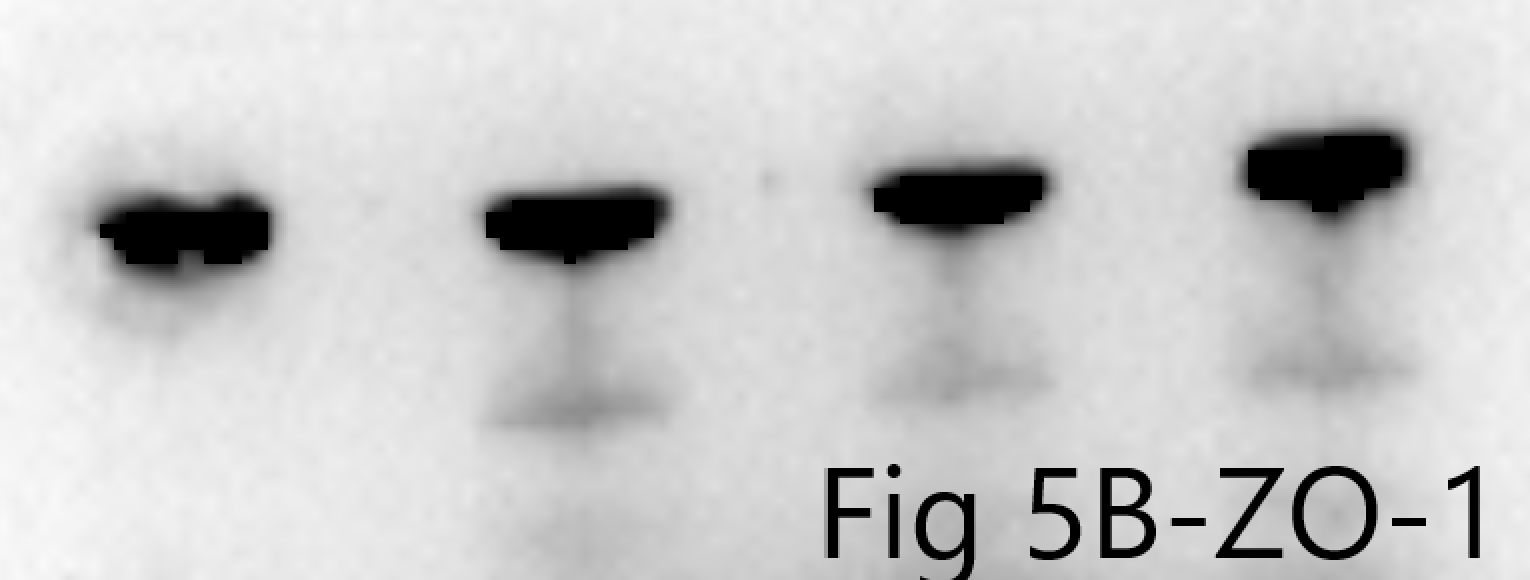

Fig 5B-ZO-1

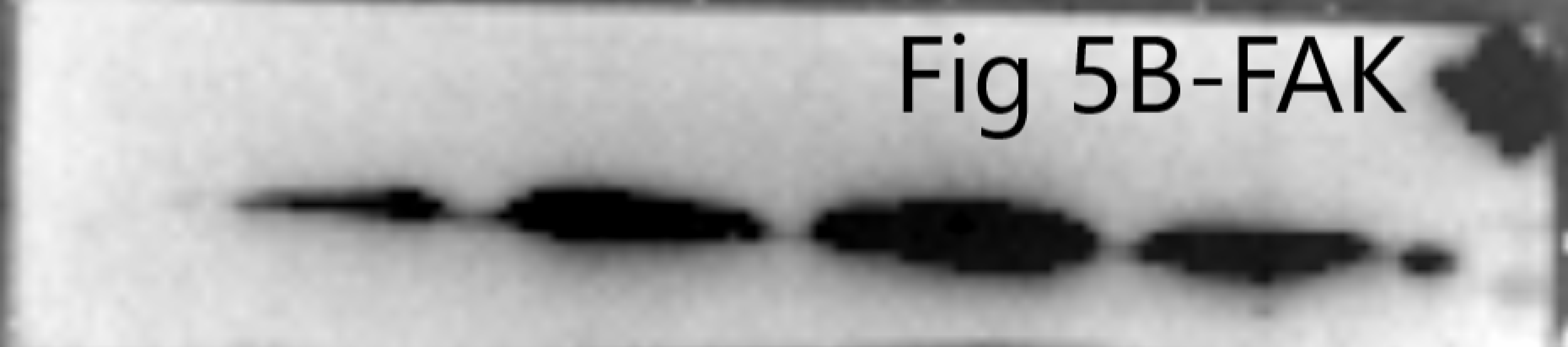
Fig 5B-FAK

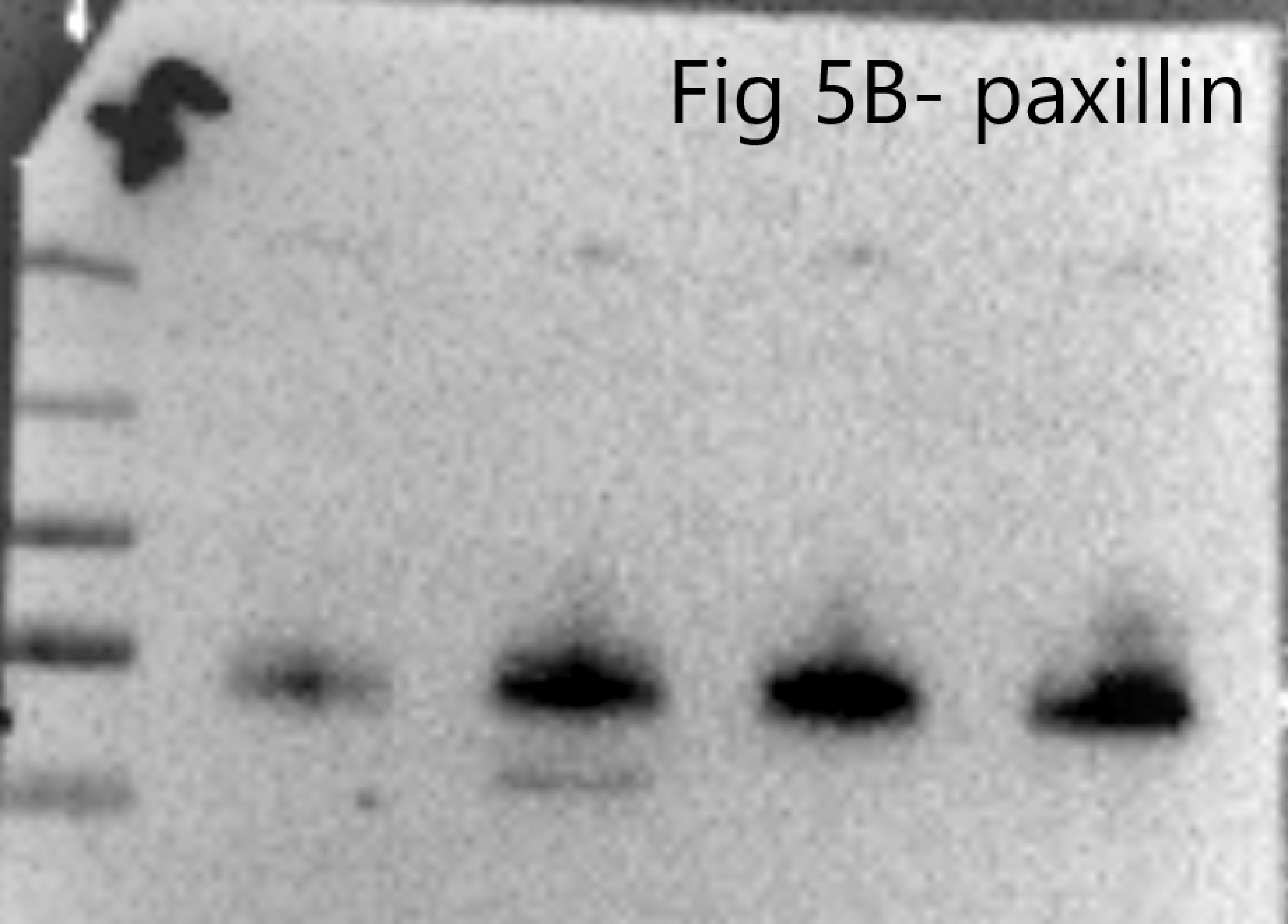
Fig 5B- paxillin

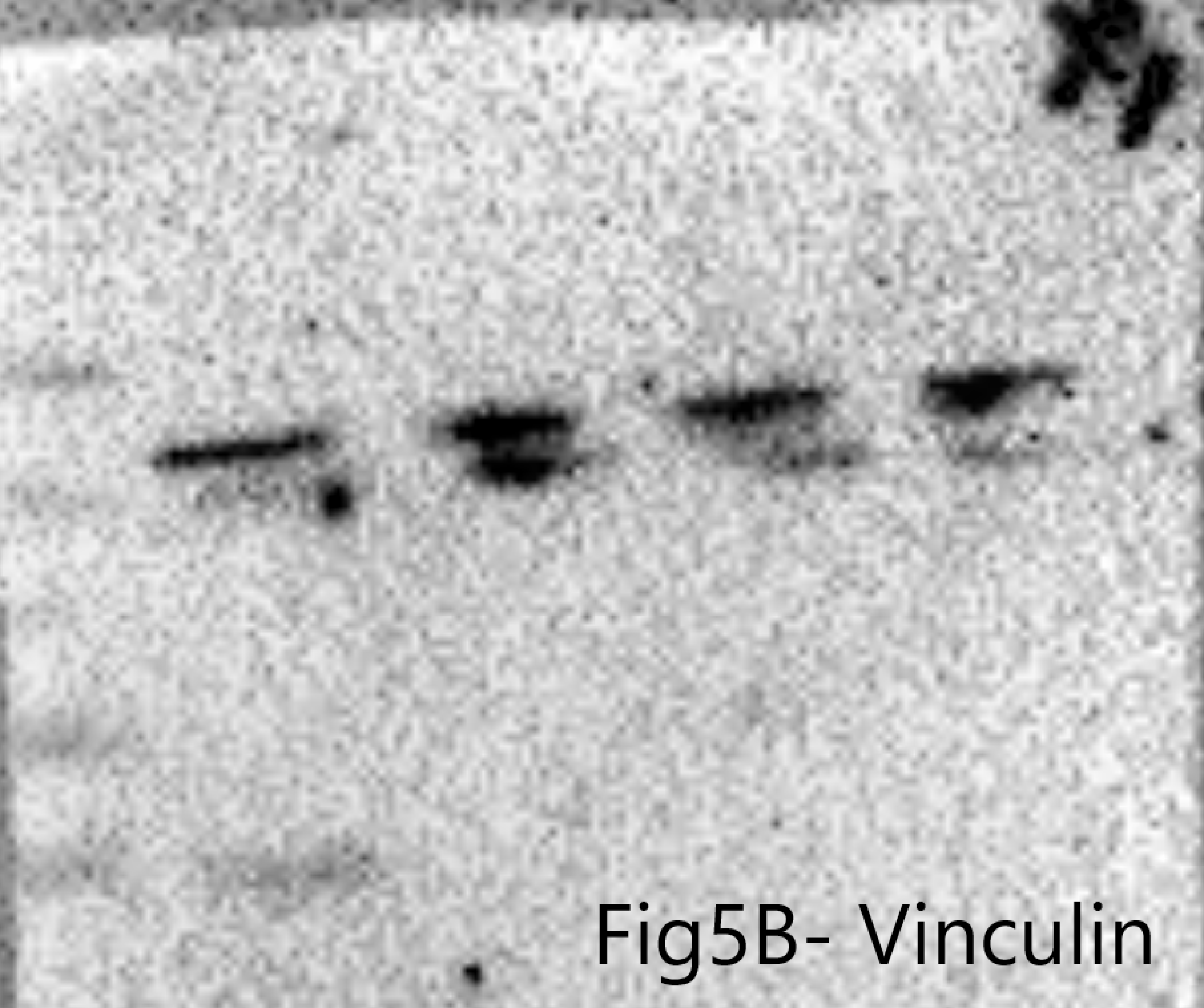

Fig5B- Vinculin

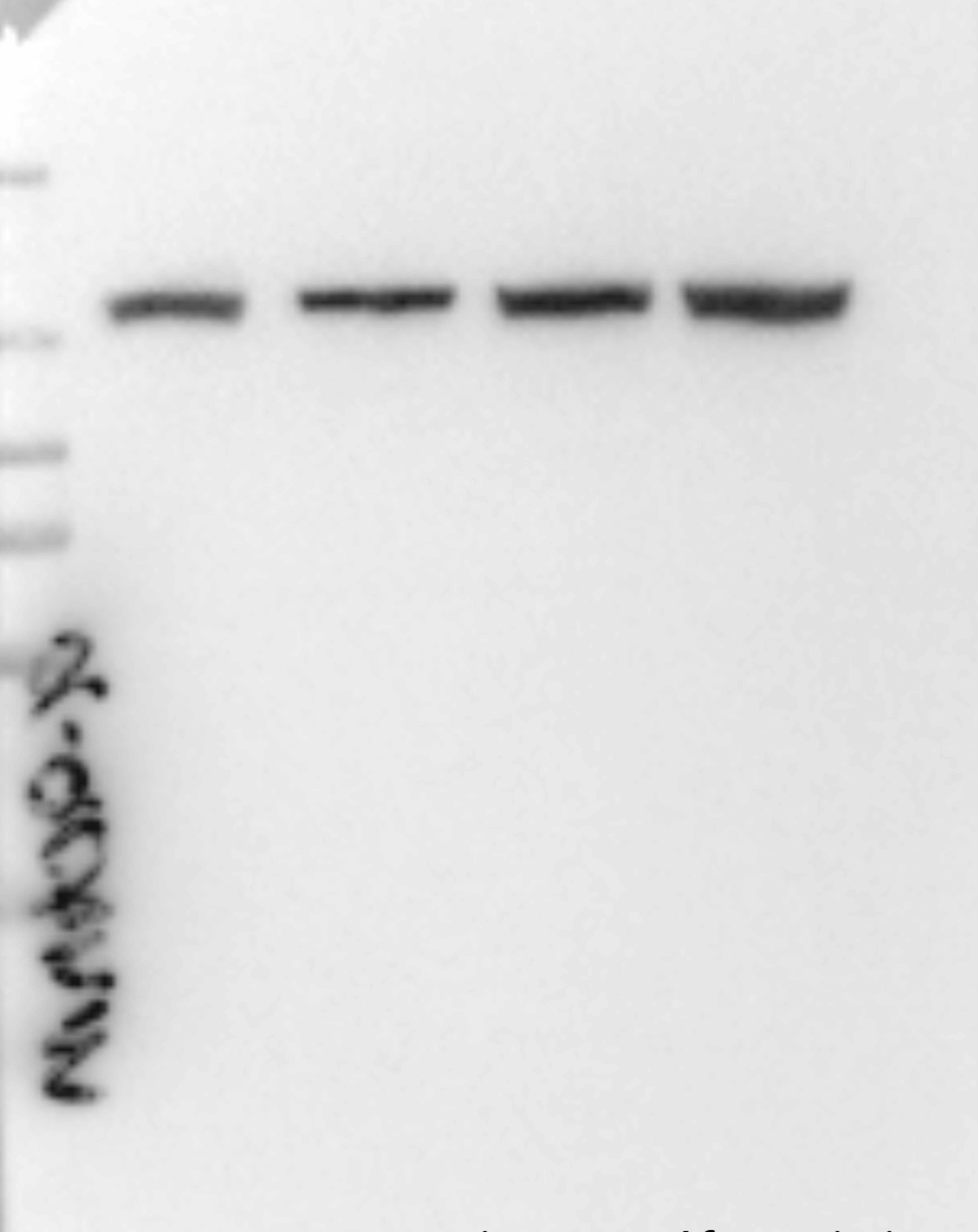

Fig 5B-alfaactinin

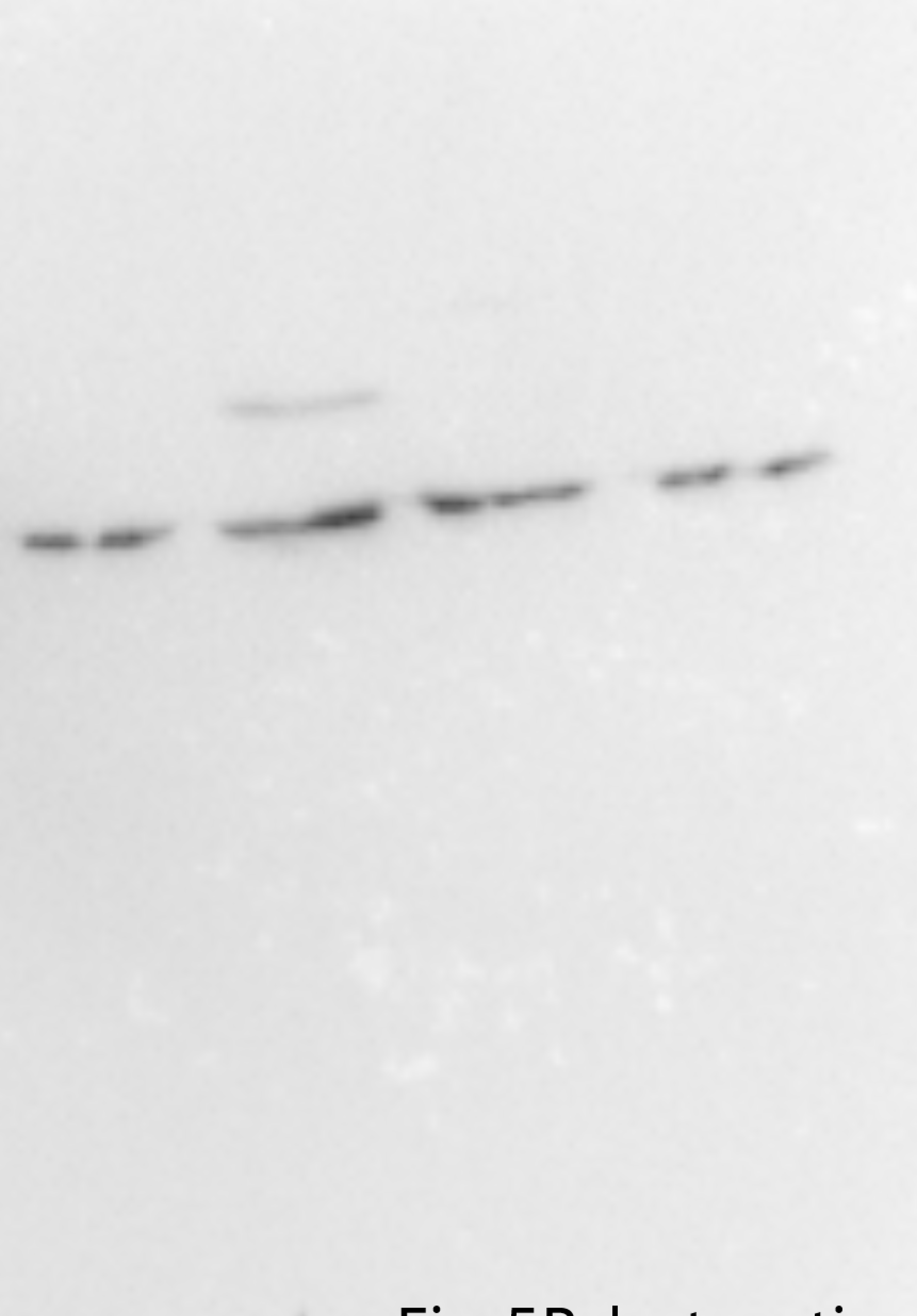

Fig 5B-betaactin

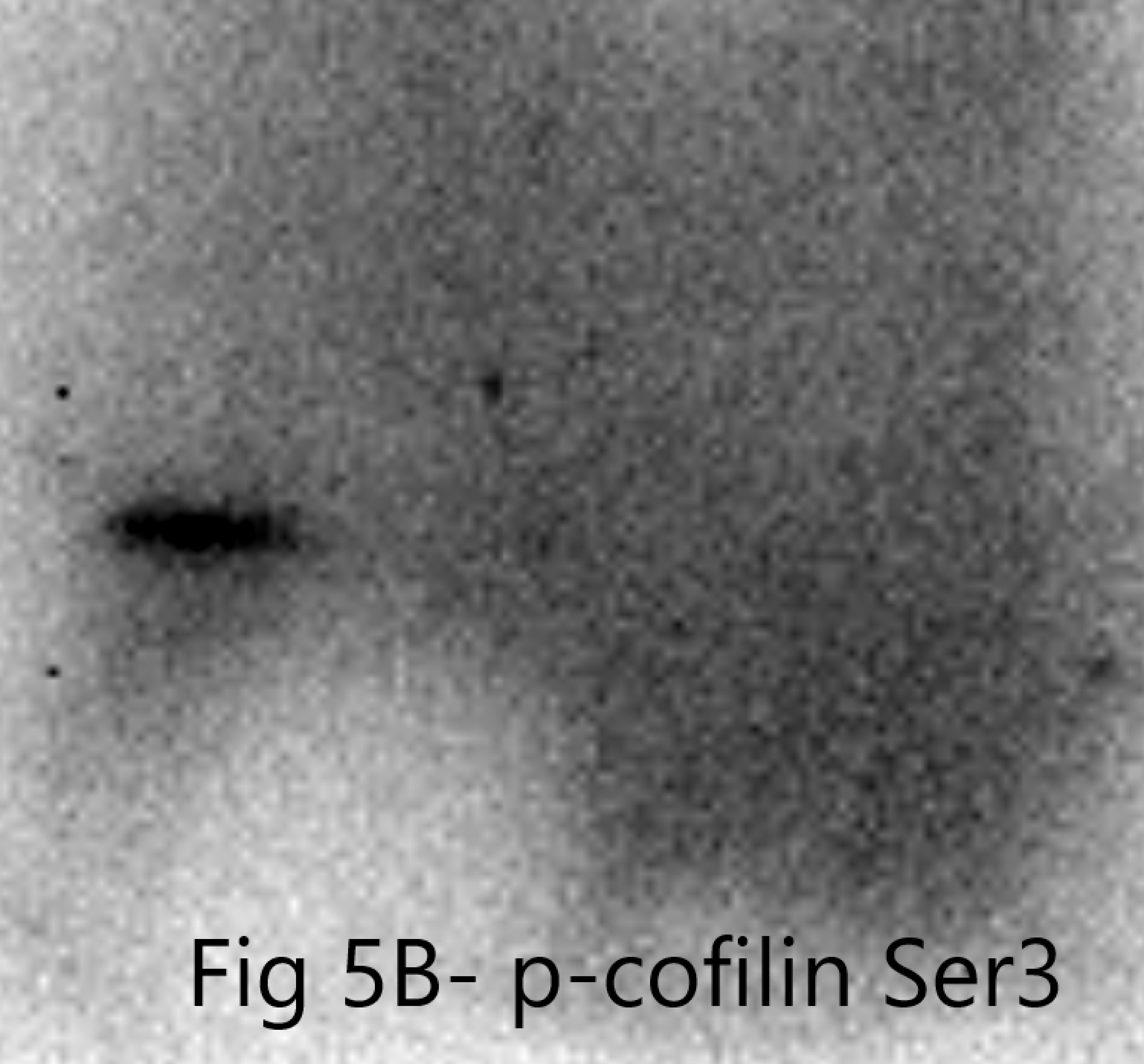

Fig 5B- p-cofilin Ser3

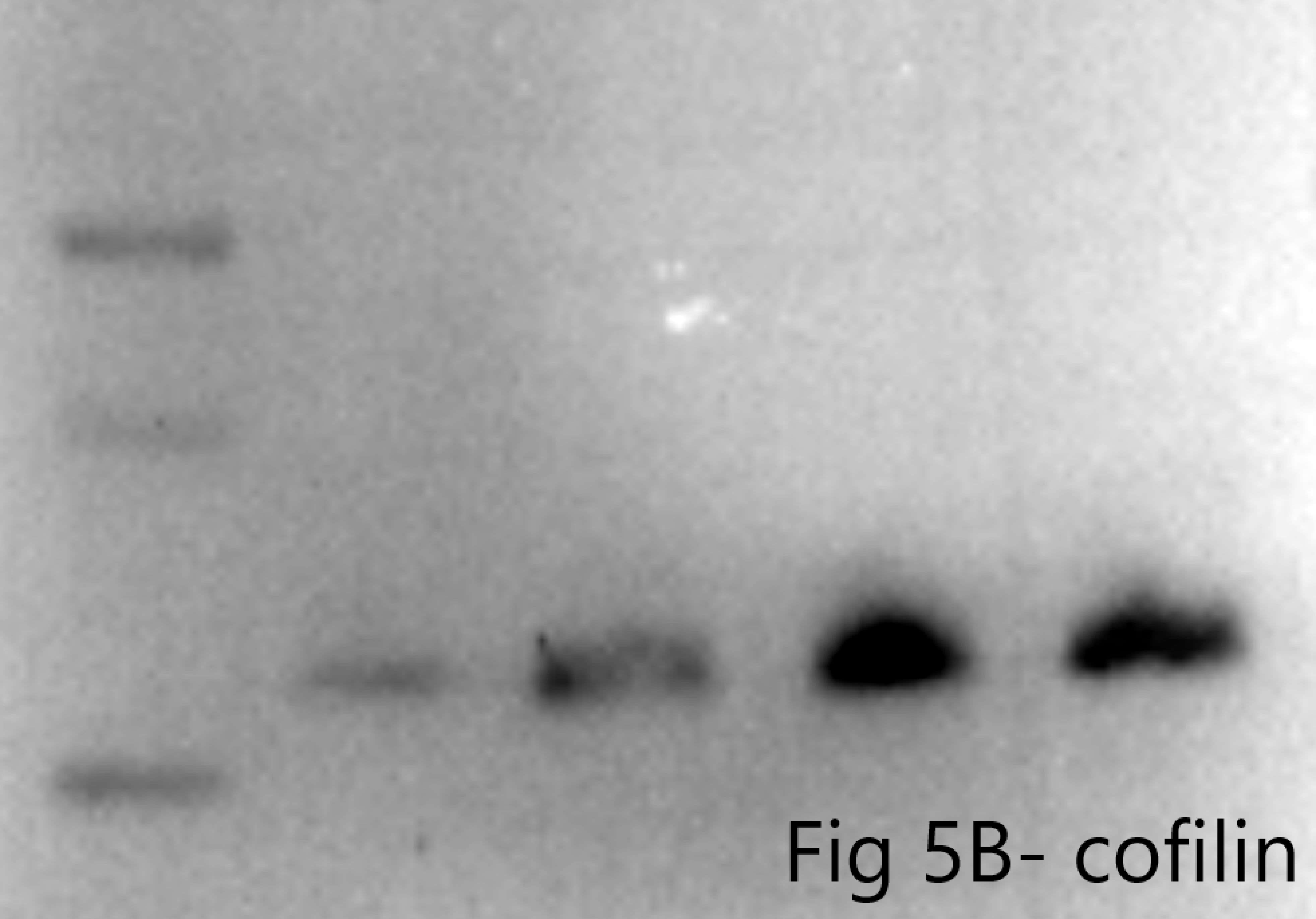

Fig 5B- cofilin

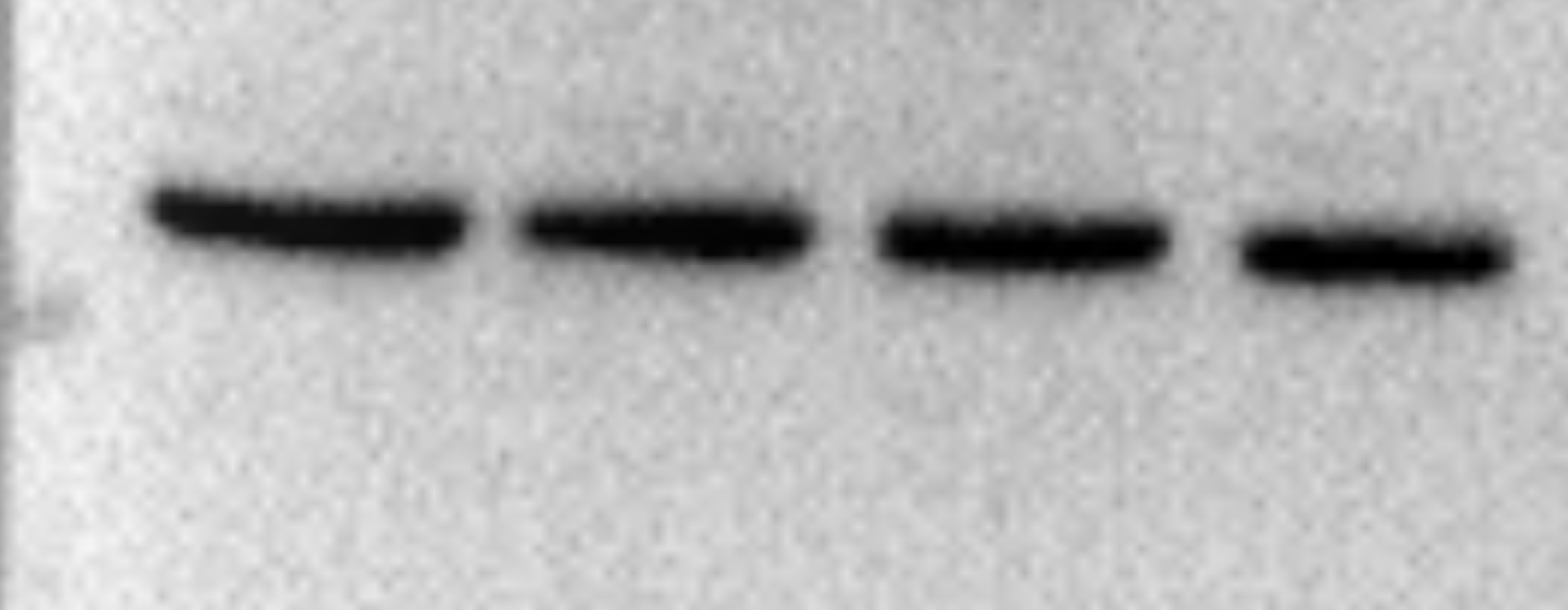

Fig 5B-GAPDH
